# Association mapping for total polyphenol content, total flavonoid content and antioxidant activity in barley

**DOI:** 10.1186/s12864-018-4483-6

**Published:** 2018-01-25

**Authors:** Zhigang Han, Jingjie Zhang, Shengguan Cai, Xiaohui Chen, Xiaoyan Quan, Guoping Zhang

**Affiliations:** 1 Department of Agronomy, Zhejiang Key Laboratory of Crop Germplasm, Zhejiang University, Hangzhou, 310058 China; 2 School of Biological Science and Technology, University of Jinan, Jinan, 250022 China

**Keywords:** Antioxidant activity, Genome-wide association study (GWAS), UDP- glycosyltransferases, Phenolic compounds, Tibetan wild barley (*Hordeum spontaneum* L.)

## Abstract

**Background:**

The interest has been increasing on the phenolic compounds in plants because of their nutritive function as food and the roles regulating plant growth. However, their underlying genetic mechanism in barley is still not clear.

**Results:**

A genome-wide association study (GWAS) was conducted for total phenolic content (TPC), total flavonoid content (FLC) and antioxidant activity (AOA) in 67 cultivated and 156 Tibetan wild barley genotypes. Most markers associated with phenolic content were different in cultivated and wild barleys. The markers bPb-0572 and bPb-4531 were identified as the major QTLs controlling phenolic compounds in Tibetan wild barley. Moreover, the marker bPb-4531 was co-located with the UDP- glycosyltransferase gene (*HvUGT*), which is a homolog to Arabidopsis *UGTs* and involved in biosynthesis of flavonoid glycosides .

**Conclusions:**

GWAS is an efficient tool for exploring the genetic architecture of phenolic compounds in the cultivated and Tibetan wild barleys. The DArT markers applied in this study can be used in barley breeding for developing new barley cultivars with higher phenolics content. The candidate gene (*HvUGT*) provides a potential route for deep understanding of the molecular mechanism of flavonoid synthesis.

**Electronic supplementary material:**

The online version of this article (10.1186/s12864-018-4483-6) contains supplementary material, which is available to authorized users.

## Background

Barley (*Hordeum vulgare* L.) is mainly used as animal feed and raw material for malt and beer production. In recent decades, its use in the production of functional or healthy food has been increasingly focused because of its rich phytochemicals [1, 2]. Some phytochemicals in cereal grains, including phenolic acids, flavonoids and anthocyanins, can reduce risk of human chronic inflammation, cardiovascular diseases, certain concers, and diabetes, and they are also involved in cell wall formation by forming bridges between polysaccharide and lignin [3, 4]. These phytochemicals in barley grains could be extracted as natural antioxidants. In fact, antioxidants have distinct effects on malting and brewing processes, including foam stability and beer bitterness, and flavonoids may affect beer taste, color and haze formation [5–8].

Identification of genes or even QTLs responsible for phenolic metabolism is necessary for the genetic improvement of the trait. Although multiple studies have identified QTLs associated with phenolic compounds in rice and sorghum, there were few studies on total phenolic content (TPC), total flavonoid content (FLC) and antioxidant activity (AOA) in barley [9, 10]. Glycosylation is one of the key steps in flavonoid biosynthesis, as it promotes the solubility, stability and bioactivity of flavonoids [11]. UDP-glycosyltransferases (UGTs) are often characterized by a conserved plant secondary product glycosyltransferase (PSPG) box of 44 amino acids binding UDP-conjugates as their activated sugar donor substrates. In plants, UGTs are involved in the transferring of glycosyl moieties into a wide range of acceptors including flavonoids in the process of glycosylation [12]. A few UGT protein coding genes are associated with biosynthesis of flavonoid glycosides in *Arabidopsis thaliana* and soybean (*Glycine max*) [13, 14]. However, little is known about the relevant genes or QTLs controlling UGTs in barley. Moreover, the genetic diversity of cultivated barley became bottlenecked due to its domestication, posing a limitation for success of barley breeding. Tibetan wild barley, as one of the progenitors of cultivated barley, shows a wider genetic diversity in agronomic traits and abiotic stress tolerance [15–17]. Phenolics might have important functions in developing tolerance to salinity, high radiation and low temperature [18–20]. Wild barley may accumulate high polyphenol content in order to adapt to harsh environments with strong ultraviolet (UV) radiation and other abiotic stresses, including extremely variable temperature [20–23]. Therefore it is meaningful to identify the genetic factors associated with polyphenol synthesis and accumulation in wild barley.

In this study, we measured TPC, FLC and AOA contents in grains of 156 Tibetan wild barley accessions and 67 cultivated barley genotypes, and performed association mappings for these traits with aims at (1) determining the genotypic variation of TPC, FLC and AOA contents in barley grains; (2) identifying the QTLs associated with phenolics and antioxidant activity in cultivated and wild barley grains. (3) evolution analysis of marker-associated gene (*HvUGT*) with *UGTs* in *Arabidopsis thaliana*.

## Methods

### Plant materials

In total 223 barley genotypes (Additional file 1: Table S1), including 156 wild types(planted in two seasons of 2013 and 2014)and 67 cultivars (they were widely planted in southern east China since 1960s, planted in 2014) were planted at Zijingang campus of Zhejiang University (Hangzhou, China, 30°22′N, 119°26′E). Each genotype was sown in a plot consisting of three rows (each row was 2 m length and row distance is 0.25 m). All plots were supplied with 150 kg/ha of N, including 40 kg/ha of N as compound fertilizer applied before seeding and 110 kg/ha of N as urea supplied at two-leaf stage and booting stage with equal amount, respectively. In addition, 180 kg/ha of potassium chloride was applied prior to seeding. The experiment was arranged in a completely randomized block with three replicates. All other field managements, including weed and disease control, were the same as those applied locally. At maturity, barley grains were harvested, dried and then stored in a cool room (4 °C) for further analysis. The grain samples were milled using grinder to pass through a 0.5 mm screen.

### Extraction of phenolic compounds

All samples were defatted by blending hexane and extracted according to Shao et al. [24] with minor modifications. Briefly, the mixture was shaken at 250 rpm for 1 h at room temperature and centrifuged at 5000 g for 15 min. After the residue was dried at 25 °C in a fume hood for 12 h, the samples were mixed with 4 ml of 80% methanol. Then the mixture was centrifuged at 5000 g for 20 min. The supernatant was collected for measurement of total phenolic content (TPC), total flavonoid content (FLC) and antioxidant activity (AOA).

### TPC determination

Total phenolic content in barley extract was determined according to Zhao et al. [24] with minor modifications. Briefly, barley extract of 0.25 mL was mixed with 1.25 mL of 0.2 mol/L Folin-Ciocalteu’s phenol reagent. After 5 min, 2 mL of 7.5% Na_2_CO_3_ solution (*w*/*v*) and 5 mL of deionized water were added. The mixture was incubated at room temperature under dark condition for 1 h on a shaking table. Then the absorbance was measured at 760 nm and a standard curve of gallic acid solution was made. TPC was expressed as micrograms of gallic acid equivalents (GAE) per gram of barley flour (μg GAE/g).

### FLC determination

Total flavonoid content was assayed according to the modified method of Shao et al. [25]. Briefly, 0.5 ml of barley extract was mixed with 3 ml ddH_2_O. Thereafter 150 μL of 5% NaNO_2_ was added and then incubated for 5 min. After adding 150 μL of 10% AlCl_3_·6H_2_O and incubating for another 5 min, 1 ml NaOH (1 M) was added and thoroughly mixed, and reacted for 15 min, and then the absorbance of solution was measured at 510 nm. FLC was expressed as micrograms of catechin equivalent (CAE) per g of barley flour (μg CAE/g).

### AOA determination

Antioxidant activity of barley extract was evaluated according to the procedure described by Saint-Cricq de Gaulejac et al. [26] with some modifications. Briefly, 0.1 ml of barley extract was mixed with 2.9 mL of 6 × 10^− 5^ M methanolic solution of 1, 1-Diphenyl-2-picrylhydrazyl Radical (DPPH). After 60 min under dark condition on a shaking table, the absorbance of solution was measured at 517 nm. Inhibition of free radical DPPH in percent (***I*** %) was calculated by using the following equation: ***I*** % = [(A_0_- A_e_)/A_0_] × 100, where A_0_ is the absorbance of the blank sample and A_e_ is the absorbance of the tested sample.

### Population structure, kinship and linkage disequilibrium (LD) analysis

The DArT markers used were derived from Diversity Arrays Technology Pty Ltd. in Australia (http://www.triticarte.com.au/content/barley_diversity_analysis.html) [27, 28]. Totally, 780 barley DArT markers (MAF > 0.03) (Additional file 2: Table S2), were used for population structure analysis using STRUCTURE software (v2.3.4) [29], setting the number of clusters (k) from 1 to 10 with 100,000 MCMC (Markov Chain Monte Carlo) and eight independent iterations were performed in an admixture model. The largest value of △k was used as the indicator of the most probable number of clusters (k) [30]. Kinship (K) was estimated using SPAGeDi (version 1.3d) software [30]. We calculated genetic distance and developed an UGPDA tree with NTSYSpc (version 2.10e). Tassel 3.0 was used to calculate linkage disequilibrium (LD) according to parameter r^2^, which is a measurement of the correlation between a pair of variables [31]. The genetic distance was derived from LD decay distance in the whole genome, when r^2^ = 0.1 using the fitted equation.

### GWAS for TPC, FLC and AOA

Association analysis of TPC, FLC, AOA was performed by TASSEL version 3.0 (http://www.maizegenetics.net) using 780 DArT markers. In order to acquire positive results in GWAS, four different models were performed [32]. Q model was applied to reduce the confounding caused by the sub-population membership. Q matrix using STRUCTURE software, and this model was expressed as y = Xβ + Qν + e, where X is the DArT marker matrix, Q is the sub-population membership matrix, e is the random error vector, and β and ν are coefficient vectors for the DArT marker and sub-population membership, respectively [29]. K model includes the kinship matrix which contributes to reducing the confounding associated with false positive results. The K model was expressed as y = Xβ + Zμ + e, where Z is the kinship matrix and μ is a vector of random genetic effects [μ ∼ N (0, 2 K)] [33]. The third approach was the Q + K model, including both sub-population membership and kinship, y = Xβ + Qν + Zμ + e [34]. Different models were evaluated for the fitness and efficiency by the quantile-quantile (Q-Q) plot using TASSEL v3.0. In the significance test −log_10_(p) > 2.0 was used as the lowest threshold.

### Putative functions of *HvUGT* (HORVU1Hr1G020560) for phenolic compounds

The nucleotide and amino acid sequence of *HvUGT* was obtained from IPK barley BLAST Server (http://webblast.ipk-gatersleben.de/barley_ibsc/) [35]. SMART (http://smart.embl-heidelberg.de/) was used to predicate functional domain of *HvUGT*. The alignment protein sequences of this gene with *Arabidopsis thaliana* were collected by the BLAST result of EnsemblePlants (http://plants.ensembl.org) and then performed by CLUSTALW with default options [36]. FastTree version 2.1.10 was used Maximus Likelihood (ML) method with 1000 replications [37].

### Statistical analysis

Data analysis for the distribution frequency of TPC, FLC and AOA were performed with the SPSS 20.0 (SPSS, Inc., Chicago, IL). Manhattan plot and Boxplot were made with the R version 3.4.0 (http://www.r-project.org/) and Sigmaplot version 12.5 (http://www.sigmaplot.com/) [38].

## Results

### Genotypic variations of TPC, FLC and AOA

**Fig. 1:**
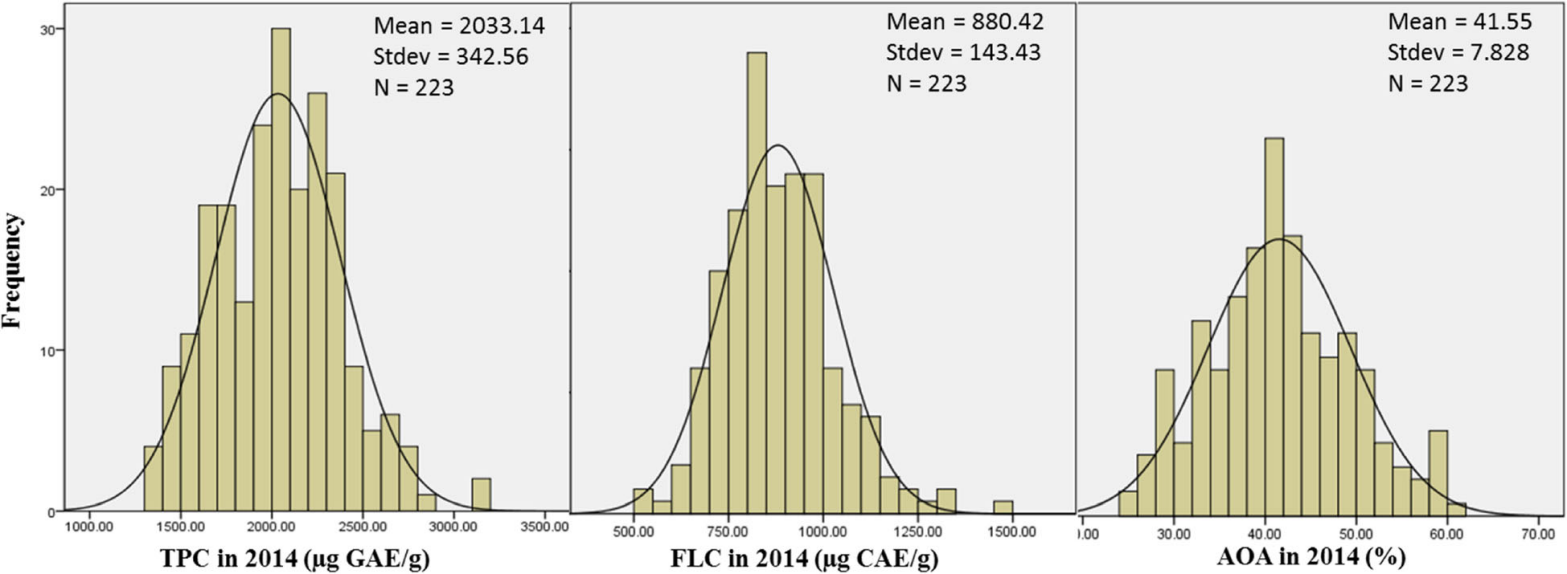
Distribution of TPC, FLC and AOA in 2014. The X-axis shows the TPC, FLC and AOA in 2014, the Y-axis shows the number of barley individuals

**Fig. 2:**
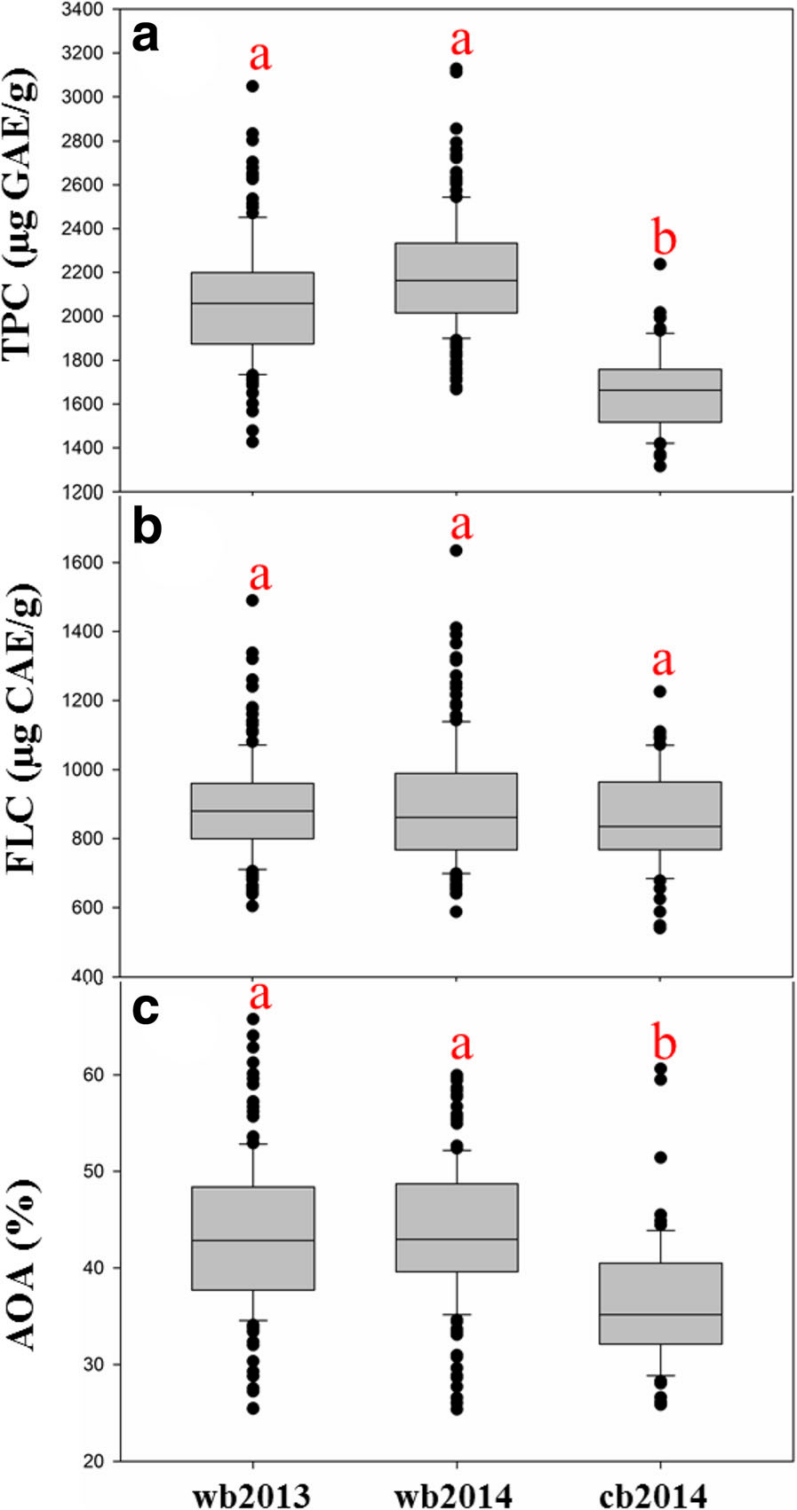
Boxplots of TPC, FLC and AOA in Tibetan wild barley and cultivated barley. **a** mean of TPC; **b** mean of TFC; **c** mean of AOA. wb2013: wild barley grown in 2013. wb2014: wild barley grown in 2014. cb: cultivated barley. For each marker, significant difference (*p*-value< 0.05) was marked by different letters

There were large variations in the concentrations of TPC, FLC and AOA among the 223 barley genotypes in 2014, including 67 cultivated and 156 wild barley genotypes (Fig. 1). The mean value and variation of each parameter in wild barley had little difference in the two experimental years (2013 and 2014) (Fig. 2). There was significantly positive correlation between the 2 years’ data for each trait (*r* = 0.81**, *r* = 0.73** and *r* = 0.70** for TPC, FLC and AOA, respectively), indicating that these traits might be mainly controlled by genetic factors (Additional file 3: Figure S1). In the year of 2014, compared with cultivated barley, wild barley had the much higher TPC and AOA concentrations, while the difference in FLC between wild and cultivated barleys was relatively small (Fig. 2).

### Population structure

Linkage disequilibrium (LD) decay distance (r^2^) was applied to determine the possibility if GWAS could be used in association analysis of phenolic compounds and genetic markers. LD decay of genetic distance in the genome of all the 223 barley genotypes was 4.18 cM (r^2^ = 0.1) (Additional file 4: Figure S2). Therefore, 801 DArT markers used in the present study were distributed over the whole genomic region with the average genetic distance of 0.70 cM and these makers were sufficient for genome wide association analysis.

**Fig. 3:**
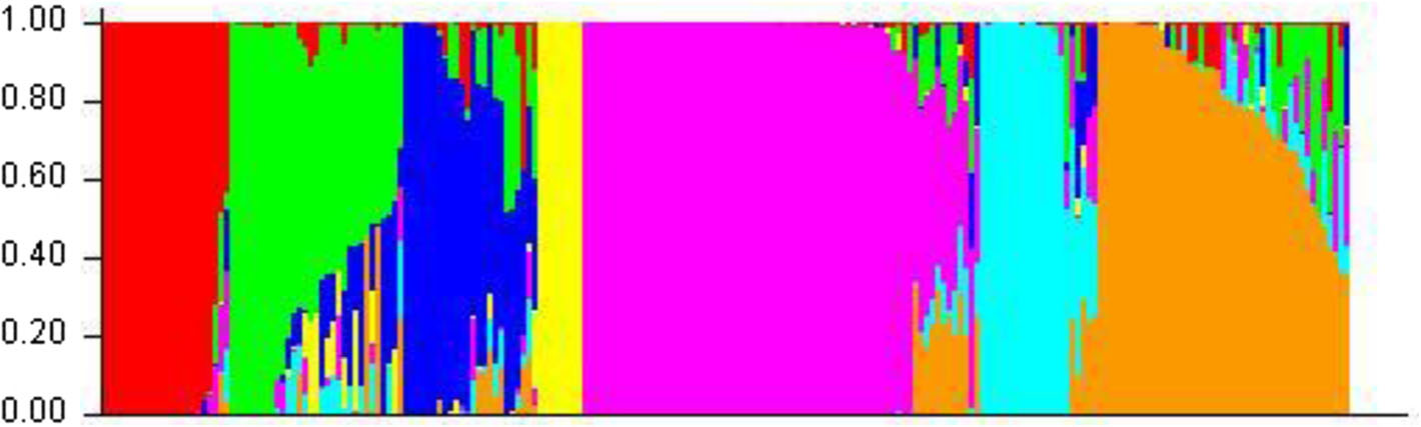
Population structure of 223 barley genotypes. Population structure of all genotypes was divided based on genetic diversity detected by 801 DArT markers with k = 7. Seven subpopulations were represented by different colors

The population structure was utilized in the model development in order to reduce nonfunctional and spurious associations caused by population stratification and an unequal distribution of alleles within these groups [39, 40]. The largest value of statistic index Δk was 64 when likelihood for sub-population (k values) calculated with STRUCTURE software were k = 7 (Additional file 5: Figure S3), indicating seven sub-populations (k = 7) were the most evident level of differentiation. These seven sub-populations consisted of 23, 31, 24, 8, 71, 21, 45 genotypes, respectively (Fig. 3; Additional file 6: Table S3). Interestingly, k7Q7 (the seventh population with k value = 7) consisted of most cultivated barleys and majority of wild barley was within other sub-populations, revealing the existence of considerable genetic diversity between Tibetan wild and cultivated barley. The result was consistent with the data from the cluster analysis (Additional file 7: Figure S4). The population structure of 223 barley genotypes was shown in Additional file 6: Table S3.

### Association mapping of TPC, FLC and AOA

**Fig. 4:**
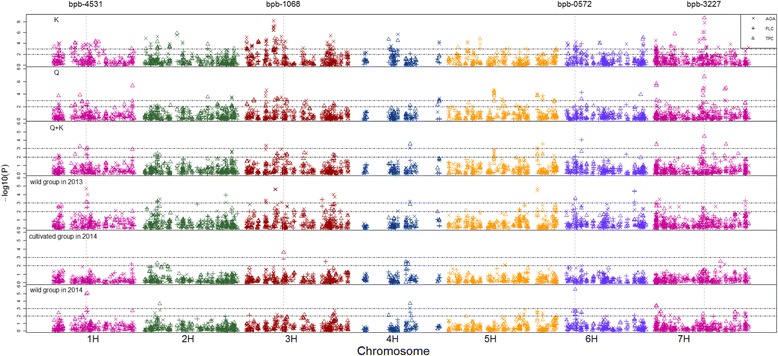
GWAS of phenolic compounds within and across 67 cultivated and 156 Tibetan wild subgroups. Three grain parameters were applied to indicate content of phenolic compounds: TPC (△), FLC (+) and TPC (×). GWA analysis was firstly conducted using three different methods: Q, K and Q + K methods. Then, Q + K method was selected to perform GWA analysis in cultivated and Tibetan wild subgroups individually. Dashed lines presented different *p* value of the four markers (bPb-4531, bPb-1068, bPb-0572 and bPb-3227) associated with these three parameters in wild and cultivated barley genotypes. Significant associations were identified using criterion of -log_10_(P) > 2 or 3

The contents of phenolic compounds in the wild and cultivated barleys were used to perform association mapping using 780 DArT markers. Three models, including Q model, K model and Q + K model, were tested to find the best fit model in association analysis, which was evaluated by Q-Q plot of *P* value distribution (Additional file 8: Figure S5). When Q or K model was applied, the observed P value was deviated from that expected, indicating the presence of abundant false positive results. Instead, the application of Q + K models reduced the number of false positive results. Thus, Q + K model was applied in this study. GWAS identified 13 unique loci for TPC, FLC and AOA in 223 barley genotypes in 2014 with the threshold -log10(p) > 3, and they were located on 6 of 7 chromosomes (except for 2H). Most of the loci were distributed on 3H, 5H and 6H, and each of them accounted for 4.0%- 7.0% of phenotypic variation (Fig. 4; Additional file 9: Table S4).

**Fig. 5:**
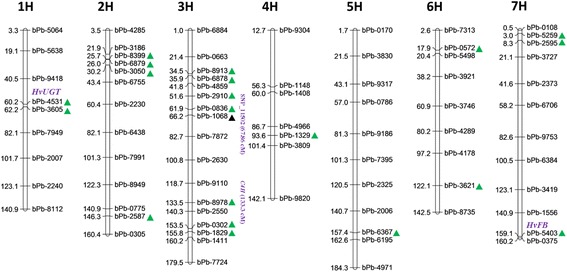
Location of 21 QTLs (*P* < 0.001) associated with TPC, TFC, AOA and candidate genes. Green triangle represented QTLs (*p* < 0.001) in Tibetan wild barley. Black triangle represented QTLs (p < 0.001) in cultivated barley. The location of *C4H*, *HvUGTs* (HORVU1Hr1G020560), *HvFB* (HORVU7Hr1G120390), was predicted by BLAST in the website of the barley genome database (http://webblast.ipk-gatersleben.de/barley/)

Association analysis was also performed in subgroups. When the threshold of significant association was set at *p* < 0.001, 20 loci were detected in the 2 years’ wild groups (Fig. 5; Additional file 9: Table S4). In the wild group, bPb-0572 (6H, 17.9 cM) was considered as the major locus controlling TPC (−log_10_(*p*) = 5.61 and 3.56 in 2 years), and contributed to 13.7 and 7.2% of phenotypic variation, respectively. BPb-4531 on Chr. 1H explained 5.8% in 2013 and 10.7% in 2014 for the variation of TPC. Notably, bPb-4531 was closely associated with all three parameters in the wild genotypes in both years except for FLC in 2013. Furthermore, the locus was significantly associated with AOA (−log_10_(*p*) = 4.72 and 3.15 in 2 years, respectively), accounting for 9.8 and 7.1% of phenotypic variation. In the cultivated barley, 7 loci were associated with these three parameters with -log10(p) > 2.5. With the threshold of -log10(p) > 3, only one marker bPb-1068 on Chr.3H was detected for TPC with -log10(p) = 3.61 and explaining 25.7% of phenotypic variation. BPb-3227 was detected using 223 genotypes with the three models and 156 Tibetan wild accessions with Q + K model in the year of 2014. It may be assumed that this locus is powerful in genetic effect for wild barley but not for cultivated barley, suggesting that Tibetan wild barley have wider genetic diversity than cultivated barley.

### Haplotype analysis of bPb-0572 and bPb-4531 for TPC in cultivated and wild barleys

**Fig. 6:**
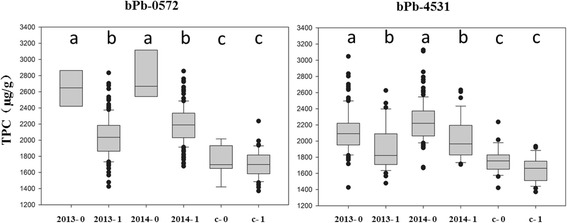
Distribution of TPC based on marker polymorphism in wild and cultivated barley. 2013: wild barley grown in 2013. 2014: wild barley grown in 2014. c: cultivated barley. 0 and 1 are DArT marker polymorphism. The markers and phenolic compounds include: bPb-0572 and bPb-4531 were associated with TPC. For each marker, significant difference (p-value< 0.05) was marked by different letters

Haplotype analysis was performed in cultivated and Tibetan barleys for the markers bPb-0572 and bPb-4531, which showed the closest association with the examined traits (Figs. 5 and 6). For bPb-0572, the marker associated with TPC in wild barley, haplotypes ‘2013-0’ and ‘2014-0’ showed higher TPC than haplotypes ‘2013-1’ and ‘2014-1’, respectively. On the contrary, there was no distinct difference in TPC between haplotypes ‘c-0’ and ‘c-1’ in the cultivated barley. For another marker associated with TPC, bPb-4531, haplotypes ‘2013-0’, ‘2014-0’ and ‘c-0’ showed higher TPC than haplotypes ‘2013-1’, ‘2014-1’ and ‘c-1’, respectively. However, in the cultivated groups, there was no significant difference.

### Candidate genes for phenolic compounds

By BLAST analysis of the sequences of DArT markers using IPK barley Blast Server (http://webblast.ipk-gatersleben.de/barley), we identified some candidate genes as shown in Fig. 5. Marker bPb-4531 was aligned to the location (Chromosome chr1H: 81,294,699-81,295,151) containing a gene (HORVU1Hr1G020560) encoding the UDP-Glycosyltransferase (UGTs) superfamily protein. Other genes were shown in Additional file 10: Table S5. Marker bPb-5403 was associated with the location (Chromosome chr7H: 651,255,911-651,256,221) containing a gene (HORVU7Hr1G120390) encoding F-box protein. In addition, the FLC-associated marker bPb-8978 (133.5 cM, 3H) was co-located with the gene encoding C_4_H (133.3 cM, 3H), which is the first Cyt-dependent mono-oxygenase of phenylpropanoid pathway for flavonoid synthesis [41].

### High similarity of HvUGT with UGT91s family in *Arabidopsis thaliana*

**Fig. 7:**
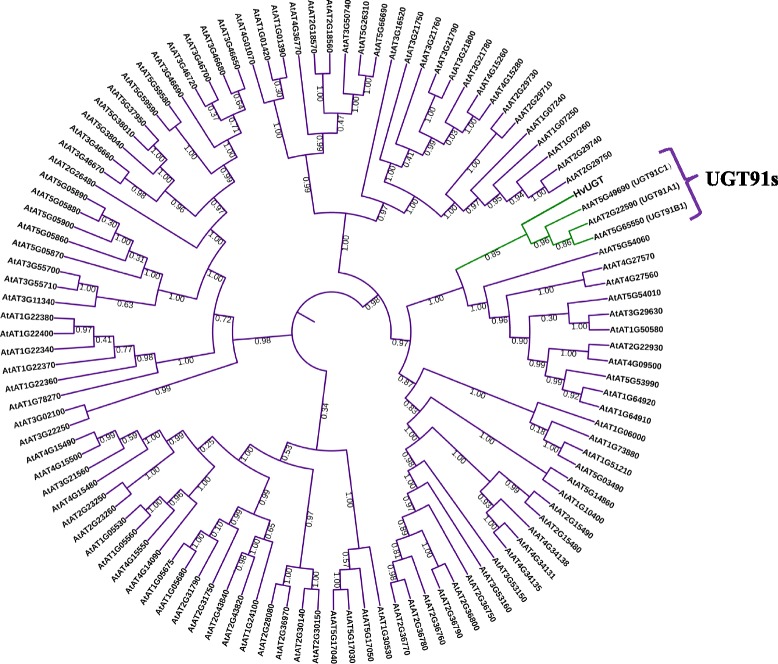
Phylogenetic tree of HvUGT and other UGT proteins in *Arabidopsis thaliana*. The UGT proteins of *Arabidopsis thaliana* was selected according to the E-value (E-value< 1 × 10^− 15^). Phylogenetic tree was constructed by FastTree with Maximus Likelihood (ML) method (Bootstrap:1000)

HvUGT protein sequence based on bPb-4531 was derived from IPK database (http://webblast.ipk-gatersleben.de/barley/) (Additional file 11: Figure S6). Then the amino acid sequence of HvUGT was analyzed by BLAST on genome of *Arabidopsis thaliana* (http://www.arabidopsis.org/) and 113 UGTs proteins (E-value< 1 × 10^− 15^) were found (Additional file 12: Table S6). All these protein sequences were listed in Additional file 10: Figure S6. The phylogenetic analysis with *Arabidopsis thaliana*, showed that HvUGT had the most similarity with UGT91s family (UGT91A1, UGT91B1 and UGT91C1), which might include functional domain catalyzing glycosyl transfer to flavonoid glycosides [42] (Fig. 7). The function domain of HvUGT was also predicted by SMART (http://smart.embl-heidelberg.de/), and the result showed that this gene consists of a similar UDPGT domain with UGT91s, indicating it is one of UDP-glucosyltransferase family members (Additional file 13: Figure S7).

## Discussion

In this study, we determined TPC, FLC and AOA in grains of 156 Tibetan wild barley accessions and 68 cultivated barley genotypes. The results showed Tibetan wild barley has a wider variation in all three traits than cultivated barley. A previous study found that Tibetan wild barley had a significantly higher ferulic acid concentration than cultivated barley [43]. Total phenolics mainly consists of phenolic acids, flavonoids and anthocyanins. The higher TPC concentration in Tibetan wild barley may be partially attributed to more ferulic acid, which is one of the major phenolic acids in barley grain. Flavonoids seem to have little contribution to the higher TPC in wild barley, as the difference in FLC between Tibetan wild and cultivated barleys was quite small. Currently, whether higher TPC in wild barley is also contributed by other phenolic compound is not clear. In this study, we found the high correlation between AOA and TPC (*r* = 0.729**) and in fact AOA,was an important component of phenolic compounds (Additional file 14: Table S7). In addition, Tibetan wild barley contained higher AOA than cultivated barley. Tibetan wild barley has the great potential for use in improving phenolic compounds of cultivated barley.

Currently, we identified 20 unique QTLs (*p* < 0.001) associated with TPC, FLC and AOA in Tibetan wild barley (Fig. 5). Most of the identified QTLs exist differently in the wild and cultivated barleys, suggesting the distinct difference in genetic diversity between the two barley groups. Some QTLs associated significantly with TPC, such as bPb-0572 and bPb-4531 were identified in Tibetan wild barleys, but not in the cultivated barley suggesting some genes or alleles controlling phenolics remain in the Tibetan wild barley and have lost in the cultivated barley [44]. The Tibetan Plateau, so called “the roof of the world” because of its very high altitude, is well known for its extreme environment, such as low and variable temperature and strong UV irradiation [15, 16, 45]. According to previous studies, wild barley might develop unique mechanisms for adapting to such harsh environments [22, 46]. Under these abiotic stresses, excessive reactive oxygen species (ROS) could be formed in plant tissues, causing damage to plant cells [47]. On the other hand, the plants exposed to severe abiotic stress preferentially accumulate phenolic compounds in order to scavenge these ROS, and alleviate peroxidation of lipids [48, 49]. Thus Tibetan wild barley has developed its own mechanism of abiotic stress tolerance by producing more phenolics. In fact, phenolic compounds also play important roles in adjusting other plant growth and metabolisms, such as differentiation, pigmentation formation [50–52].

Although phenolic acids are components of phenolics and may partially contribute to TPC and AOA, only one common locus (bPb-0836) was found in this study (Fig. 5). A possible explanation would be that the individual phenolic acids only account for a small amount of total phenolics. Mohammadi et al. [53] reported three QTLs for total phenolics in barley genotypes collected from eight US breeding programs. One of them (SNP_11502, 3H, 67.86 cM) was located near the locus (bPb-1608, 3H, 66.16 cM), which was found in this study to be associated with TPC in the cultivated barley.

The release of barley genome sequence will facilitate the prediction of possible candidate genes based on the identified markers [35]. The UGT proteins are partly involved in the synthesis of anthocyanins and flavonoids [54]. Moreover, UGTs in plants participate in the formation of 3-O-glucoside and 4’-O-diglucoside, thus promoting flavonoid glycosylation [55]. In the current study, marker bPb-4531 was located on the Chr. 1H, which contains a gene encoding the UDP-glycosyltransferase (UGT) superfamily protein (HvUGT). According to the phylogenetic tree, *HvUGT* is closely related to gene family *UGT91s* in *Arabidopsis thaliana*. *UGT91A1,* one of the *UGT91* family members in *Arabidopsis thaliana* might be involved in the synthesis of flavonol glycosides [42, 56]. Without UGT91A1 activity, there would be no galactose transferring into kaempferitrin, indicating that this protein specifically accepts and transfers galactose [57]. In fact this protein was found to have the similar specificity to flavonoid as UGT73C6 and UGT78D1 did [58]. Thus, HvUGT may be involved in pathway of flavonoids. Markers bPb-5403 was associated with contig_41360 containing a gene (HORVU7Hr1G120390) encoding F-box protein. Kelch domain-containing F-box proteins (KFBs) negatively regulated naringenin chalcone accumulation, thus reducing the production of polyphenols [59, 60]. Kelch domain-containing F-box proteins (KFBs) have also been found to regulate the biosynthesis of phenylpropanoids, such as anthocyanins, flavonoids, phenolic ester and lignin [61]. In addition, the FLC-associated marker bPb-8978 (133.5 cM, 3H) was co-located with the gene encoding C4H (133.3 cM, 3H), which is the first Cyt-dependent mono-oxygenase of phenylpropanoid pathway for flavonoid synthesis, which have significant effect on lignin development [41]. In short, these identified genes are closely associated with phenolic metabolism, although their functions are still unclear in barley. Furthermore, the detected markers in this study should be helpful for better understanding the genetic control of TPC, FLC and AOA in barley.

## Conclusions

The current results showed the wide variation among barley genotypes and obvious difference between Tibetan wild and cultivated barleys in grain TPC, FLC and AOA. Tibetan wild barley had higher concentration and wider genetic diversity of phenolic compounds than cultivated barley. Most QTLs were identified in the Tibetan wild barley and only one was detected in cultivated barley with *p* < 0.001, indicating Tibetan wild barley is potentially useful in barley breeding for improving phenolic compounds. The marker (bPb-4531) was co-located with HvUGT, which is a homolog to Arabidopsis UGT and may be responsible for flavonoid synthesis. This finding may serve as the foundation for further in-depth studies on molecular mechanism of natural variation in phenolic compounds.

## Additional files

Additional file 1: Table S1. Cultivated and wild barley genotypes used in this study. C: cultivated barley; W: wild barley; XZ: Tibetan wild barley. (XLSX 21 kb)

Additional file 2: Table S2. MAF and PIC of 801 DArT markers. MAF, Minor allele frequency; PIC, polymorphic information content. (XLSX 40 kb)

Additional file 3: Figure S1. Correlation analysis of TPC, FLC and AOA in Tibetan wild barley. (a) correlation for TPC between 2013 and 2014; (b) correlation in FLC between 2013 and 2014; (c) correlation in AOA between 2013 and 2014. **, represents significant correlation at *P* < 0.01. (DOCX 80 kb)

Additional file 4: Figure S2. Decay of linkage disequilibrium of the population of 223 genotypes based on 801 DArT markers. The X-axis showed the genetic distance, the Y-axis showed the r2, the squared allele frequency correlations, which is a measurement of the correlation between a pair of variables. (DOCX 66 kb)

Additional file 5: Figure S3. Estimation of the most probable number of clusters (k). (DOCX 27 kb)

Additional file 6: Table S3. The value of population structure of 223 genotypes. Each genotype belongs to the population with the highest value calculated by STRUCTURE software. (XLSX 25 kb)

Additional file 7: Figure S4. Phylogenetic tree (UPGMA) of 223 barley genotypes based on 801 DArT markers. C, cultivated barley; X, Tibetan wild barley. (DOCX 831 kb)

Additional file 8: Figure S5. Quartile-quartile (Q-Q) plots of *P* value for 223 genotypes under three GWAS models. The black line is an expected one under the null distribution. Red symbol represents the observed *P* values for AOA, blue for FLC and green for TPC. (DOCX 425 kb)

Additional file 9: Table S4. List of DArT markers (−log10(p) > 2.5) in 223 genotypes and within subgroups. These markers were identified using Q + K model with significance threshold as –log10(p) > 2.5. R2 (marker) denotes the contribution of the marker for phenotypic variation. (DOCX 35 kb)

Additional file 10: Table S5. Genes around the region of marker bPb-4531. NOTE: Genomic location is showed in physical distance. (XLSX 12 kb)

Additional file 11: Figure S6. Protein sequences of HvUGT (HORVU1Hr1G020560) in barley and UGTs in Arabidopsis thaliana. (PDF 187 kb)

Additional file 12: Table S6. BLAST results of HvUGT with Arabidopsis thaliana. Note: These data are derived from The Arabidopsis Information Resource. (http://plants.ensembl.org). (XLSX 17 kb)

Additional file 13: Figure S7. Functional domain prediction of HvUGT. a: function domain in HvUGT; b: function domain in UGT91C1. Pink color represents low complexity sequence, the number marks amino acid sequence sites. (PPTX 90 kb)

Additional file 14: Table S7. Correlation analysis of TPC, FLC and AOA in 223 accessions. ** represent significant correlation at P < 0.01. (DOCX 15 kb)

## Abbreviations

AOA: Antioxidant activity
C4H: Cinnamic acid 4-hydroxylase
CAE: Catechin equivalent
F3H: Flavanone 3-hydroxylase
FLC: Total flavonoid content
GAE: Gallic acid equivalent
GWAS: Genome-Wide Association Study
KFBs: Kelch domain-containing F-box proteins
TPC: Total phenolic content
UGT: UDP-Glycosyltransferase
