# Supplementary figures and images for: Association mapping for total polyphenol content, total flavonoid content and antioxidant activity in barley
Source: BMC Genomics. 2018 Jan 25;19:81. doi: 10.1186/s12864-018-4483-6 (PMC5784657; doi:10.1186/s12864-018-4483-6)

**Figure S1**

**
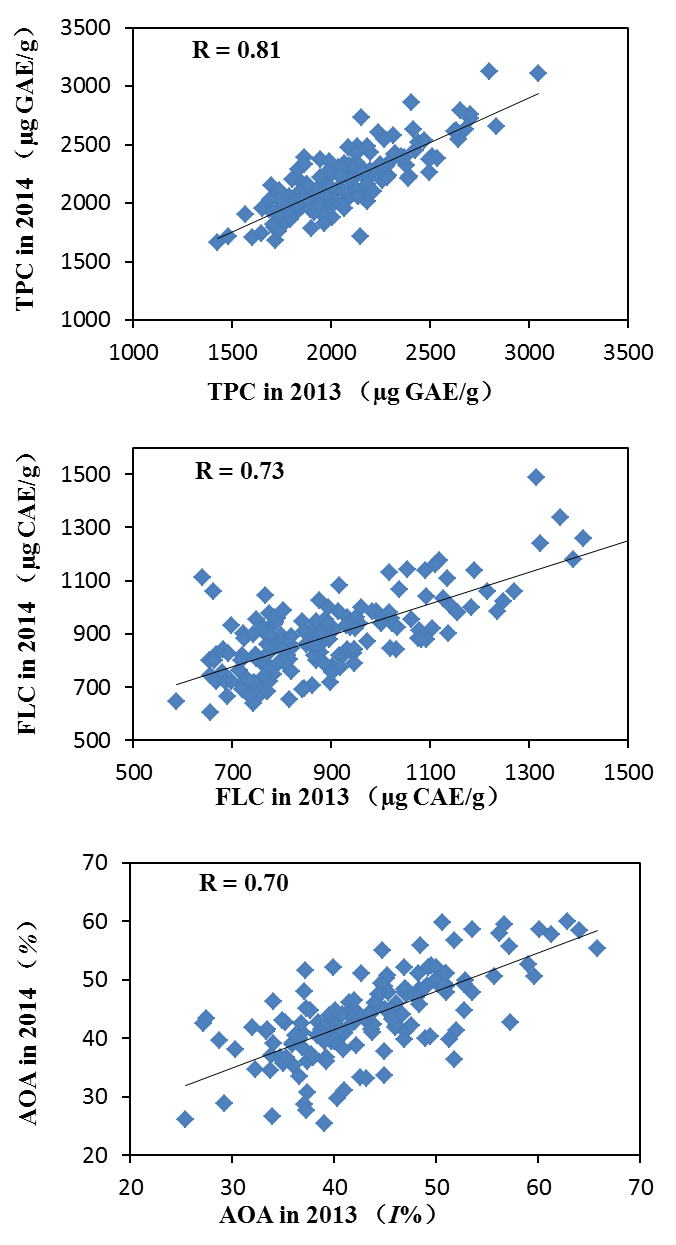
**

Supplement: Supplementary file 3 — Correlation analysis of TPC, FLC and AOA in Tibetan wild barley. (a) correlation for TPC between 2013 and 2014; (b) correlation in FLC between 2013 and 2014; (c) correlation in AOA between 2013 and 2014. **, represents significant correlation at P < 0.01. (DOCX 80 kb) [file 12864_2018_4483_MOESM3_ESM.docx]

**Figure S2**


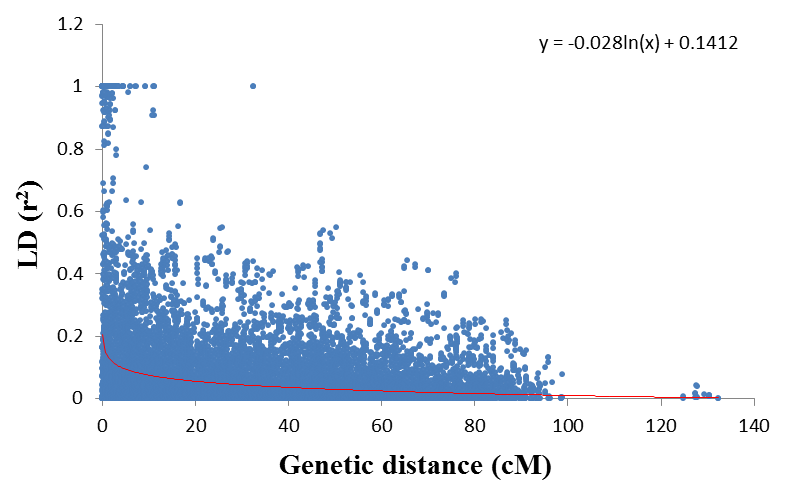

Supplement: Supplementary file 4 — Decay of linkage disequilibrium of the population of 223 genotypes based on 801 DArT markers. The X-axis showed the genetic distance, the Y-axis showed the r2, the squared allele frequency correlations, which is a measurement of the correlation between a pair of variables. (DOCX 66 kb) [file 12864_2018_4483_MOESM4_ESM.docx]

**Figure S3**


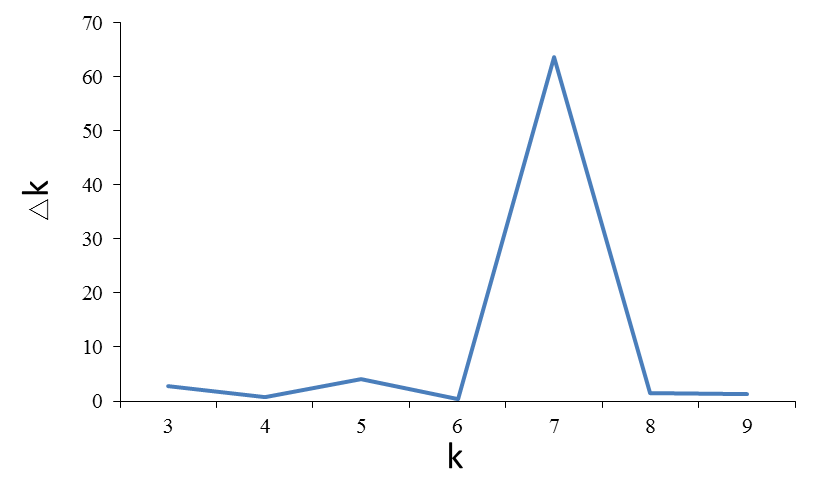

Supplement: Supplementary file 5 — Estimation of the most probable number of clusters (k). (DOCX 27 kb) [file 12864_2018_4483_MOESM5_ESM.docx]

**Figure S4**


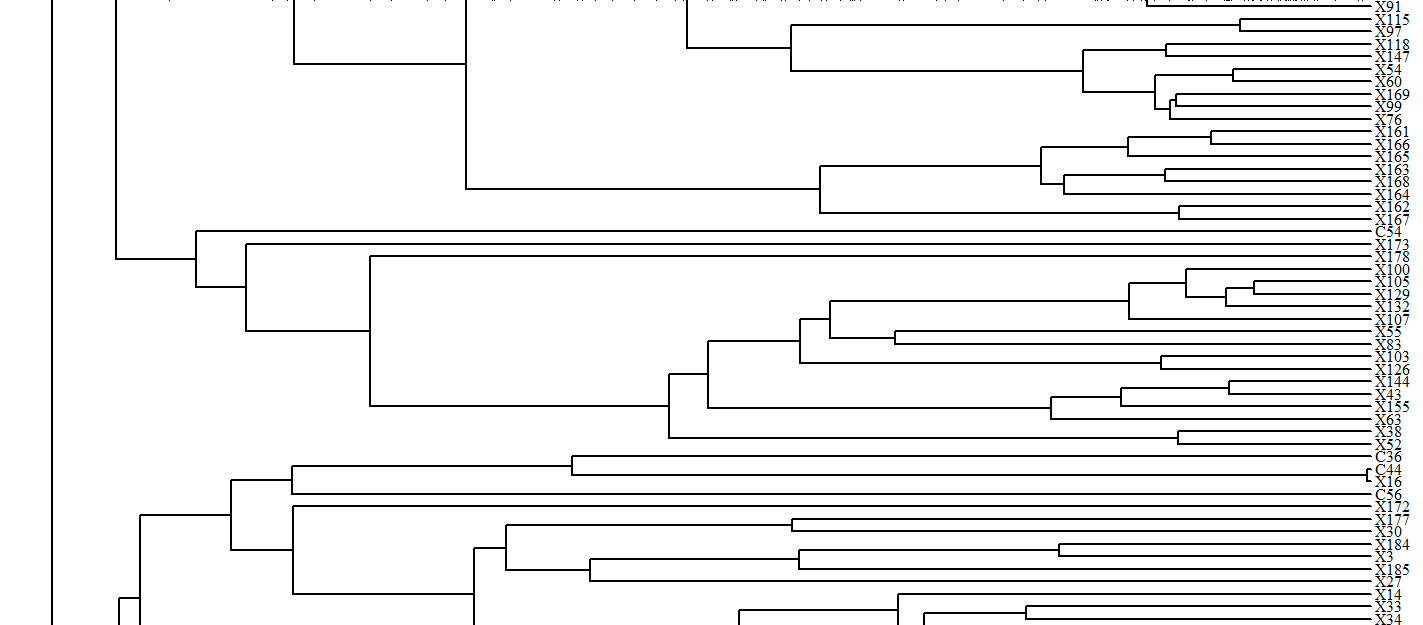

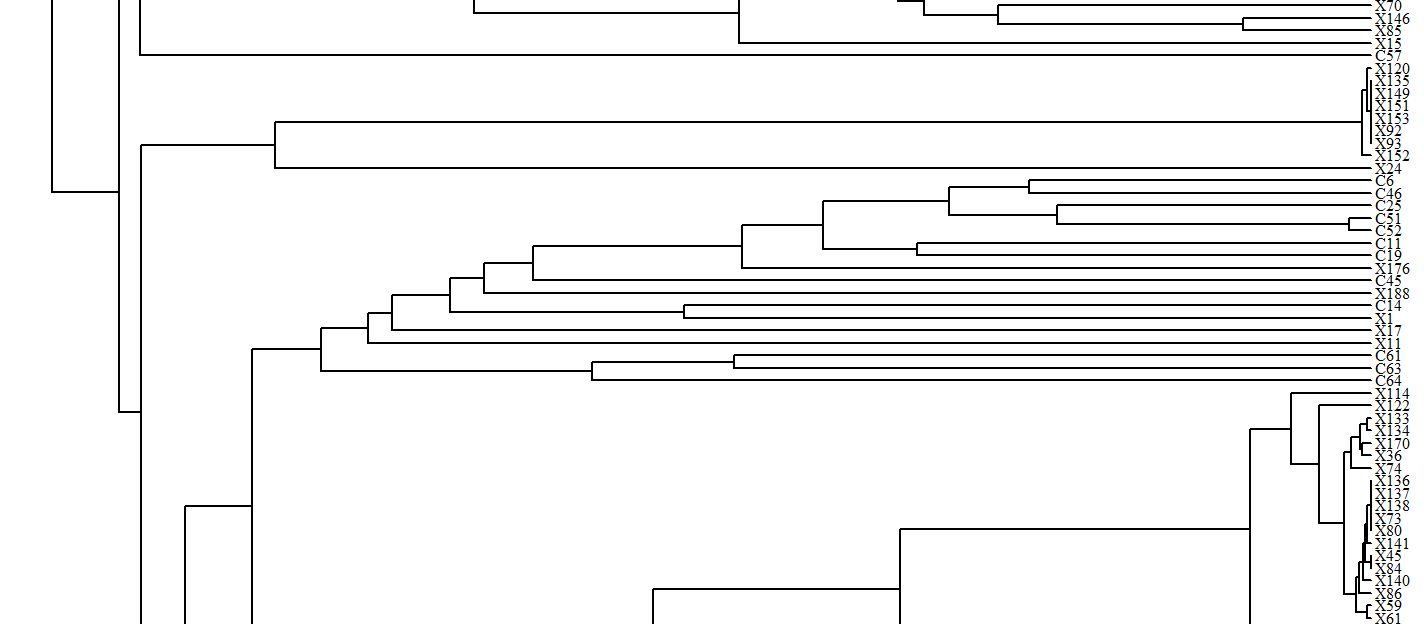

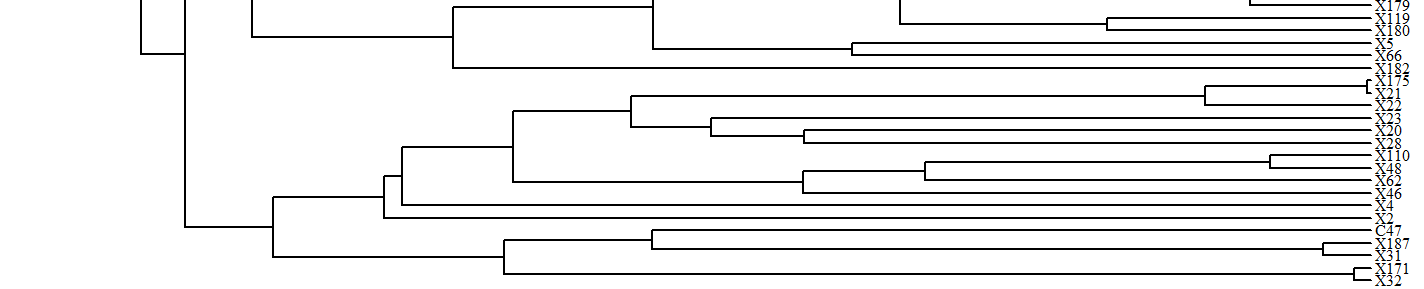

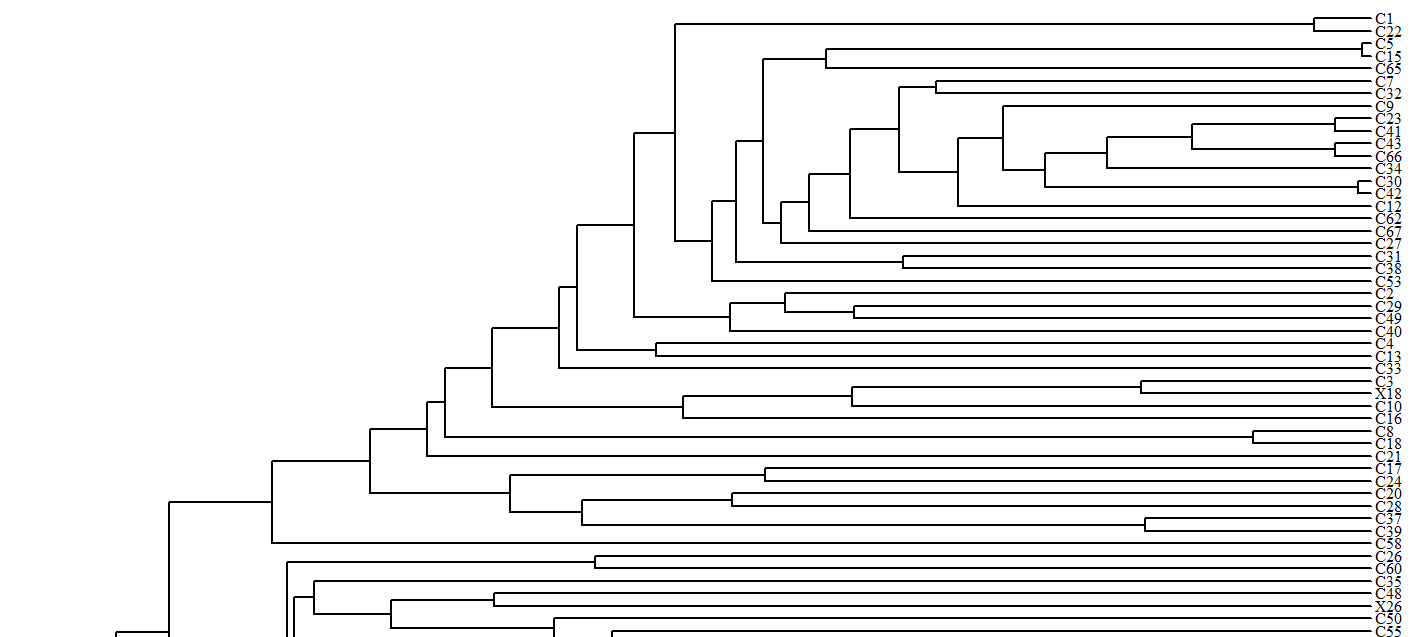

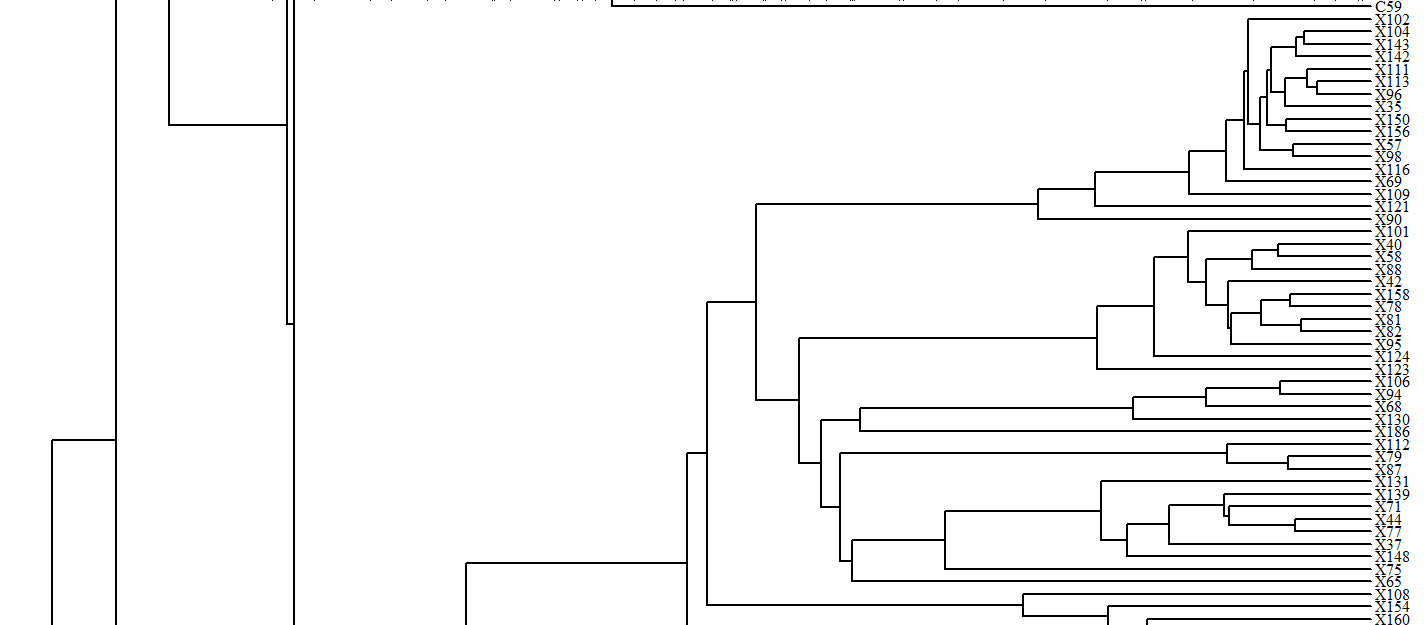


0.60

0.80

0.70

1.00

0.90

**Coefficient**

Supplement: Supplementary file 7 — Phylogenetic tree (UPGMA) of 223 barley genotypes based on 801 DArT markers. C, cultivated barley; X, Tibetan wild barley. (DOCX 831 kb) [file 12864_2018_4483_MOESM7_ESM.docx]

**Figure S5**

**
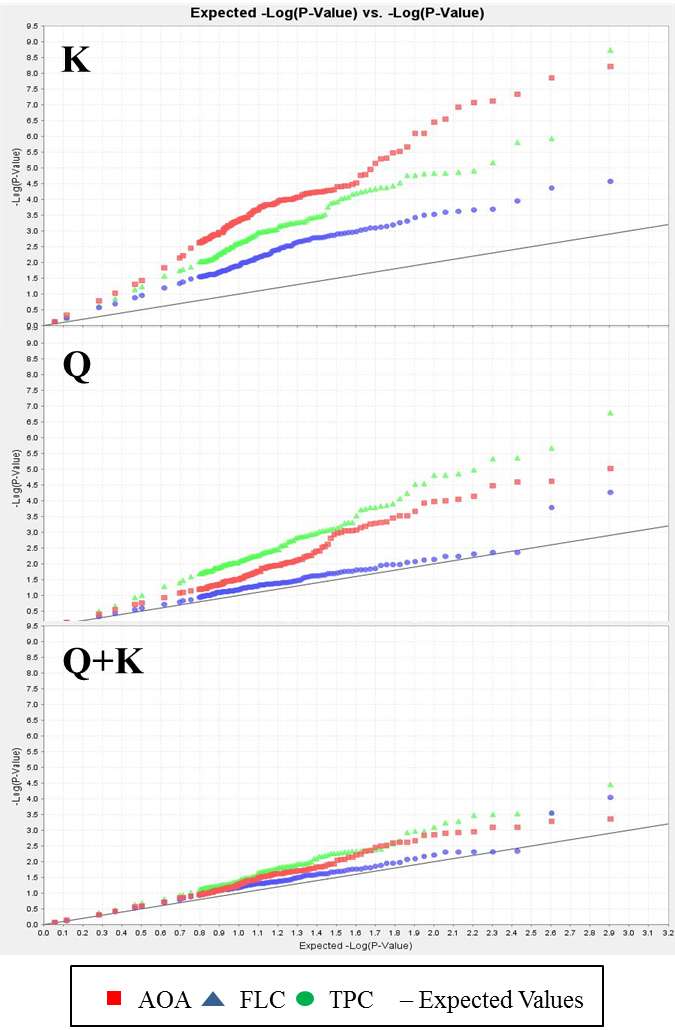
**

Supplement: Supplementary file 8 — Quartile-quartile (Q-Q) plots of P value for 223 genotypes under three GWAS models. The black line is an expected one under the null distribution. Red symbol represents the observed P values for AOA, blue for FLC and green for TPC. (DOCX 425 kb) [file 12864_2018_4483_MOESM8_ESM.docx]

## Slide 1
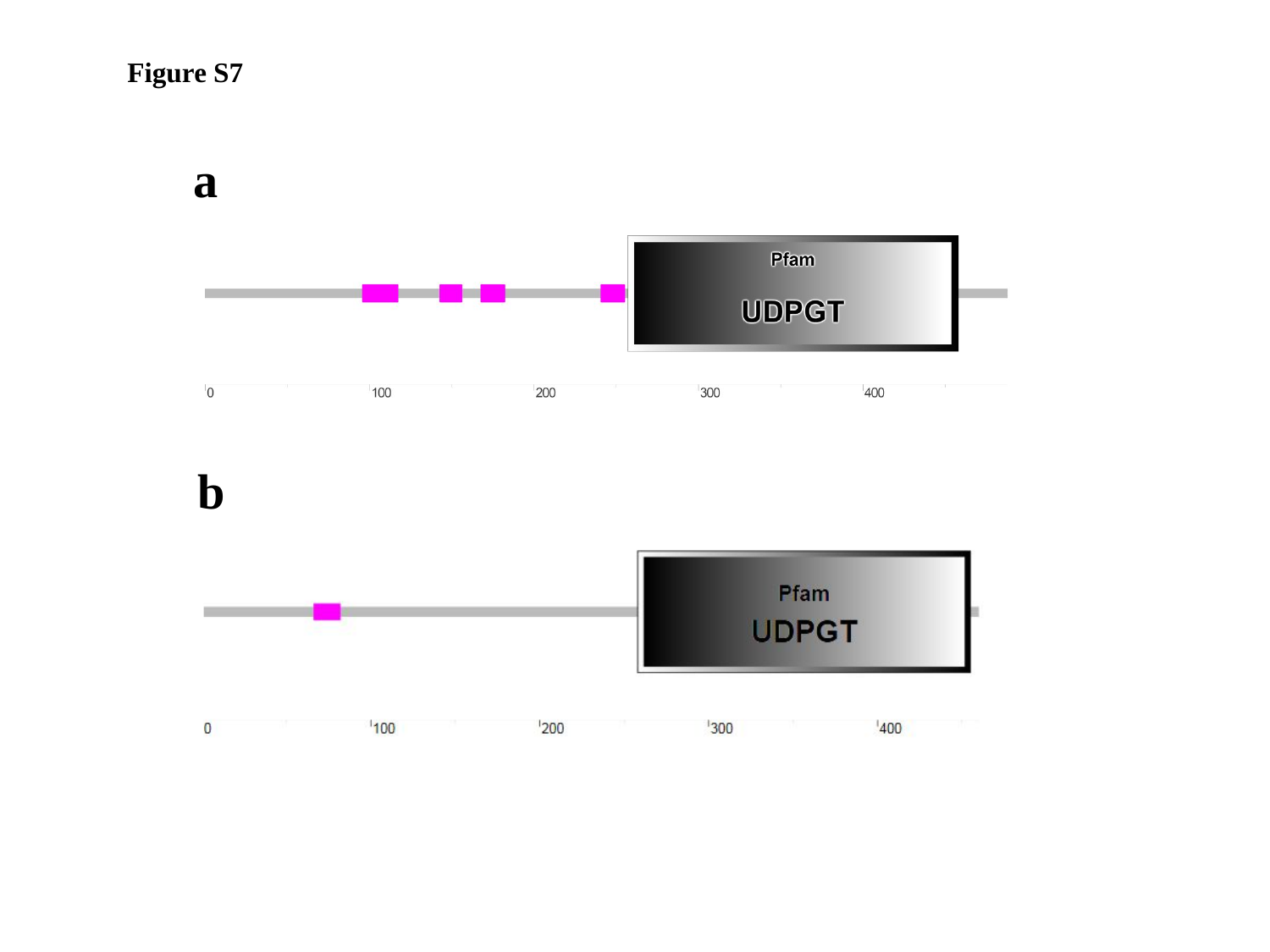

Figure S7
a
b

Supplement: Supplementary file 13 — Functional domain prediction of HvUGT. a: function domain in HvUGT; b: function domain in UGT91C1. Pink color represents low complexity sequence, the number marks amino acid sequence sites. (PPTX 90 kb) [file 12864_2018_4483_MOESM13_ESM.pptx]
