# Supplementary material for: Association mapping for total polyphenol content, total flavonoid content and antioxidant activity in barley
Source: BMC Genomics. 2018 Jan 25;19:81. doi: 10.1186/s12864-018-4483-6 (PMC5784657; doi:10.1186/s12864-018-4483-6)
Supplement: Supplementary file 9 — List of DArT markers (−log10(p) > 2.5) in 223 genotypes and within subgroups. These markers were identified using Q + K model with significance threshold as –log10(p) > 2.5. R2 (marker) denotes the contribution of the marker for phenotypic variation. (DOCX 35 kb) [file 12864_2018_4483_MOESM9_ESM.docx]

**Table S4.** List of DArT markers (–log_10_(p)>2.5) in 223 genotypes and within subgroups.

|  | Trait | Marker | Chr. | Distance (cM) | -log_10_(p) | R^2^ |
| --- | --- | --- | --- | --- | --- | --- |
| Q+K | TPC2014 | bPb-9423 | 1H | 48.95 | 3.28 | 0.047 |
|  | TPC2014 | bPb-3605 | 1H | 62.23 | 3.1 | 0.047 |
|  | TPC2014 | bPb-4531 | 1H | 60.21 | 2.97 | 0.043 |
|  | TPC2014 | bPb-0589 | 1H | 141.24 | 2.92 | 0.042 |
|  | TPC2014 | bPb-5295 | 3H | 102.23 | 2.5 | 0.034 |
|  | TPC2014 | bPb-1329 | 4H | 93.64 | 3.56 | 0.052 |
|  | TPC2014 | bPb-8150 | 6H | 28.84 | 2.67 | 0.038 |
|  | TPC2014 | bPb-3227 | 7H | 87.39 | 4.46 | 0.068 |
|  | TPC2014 | bPb-4389 | 7H | 125.4 | 3.53 | 0.052 |
|  | TPC2014 | bPb-5259 | 7H | 3.02 | 3.51 | 0.052 |
|  | TPC2014 | bPb-7437 | 7H | 84.95 | 3.25 | 0.05 |
|  | FLC2014 | bPb-3138 | 5H | 166.59 | 3.55 | 0.06 |
|  | FLC2014 | bPb-8150 | 6H | 28.84 | 4.04 | 0.073 |
|  | AOA2014 | bPb-8734 | 2H | 156.45 | 2.67 | 0.043 |
|  | AOA2014 | bPb-6222 | 2H | 156.45 | 2.52 | 0.04 |
|  | AOA2014 | bPb-6105 | 2H | 156.75 | 2.62 | 0.043 |
|  | AOA2014 | bPb-8913 | 3H | 34.49 | 3.29 | 0.055 |
|  | AOA2014 | bPb-6878 | 3H | 35.93 | 3.37 | 0.057 |
|  | AOA2014 | bPb-0836 | 3H | 61.87 | 2.93 | 0.047 |
|  | AOA2014 | bPb-1329 | 4H | 93.64 | 3.09 | 0.051 |
|  | AOA2014 | bPb-0602 | 5H | 81.35 | 2.87 | 0.048 |
|  | AOA2014 | bPb-4891 | 5H | 81.35 | 2.62 | 0.043 |
|  | AOA2014 | bPb-6367 | 5H | 157.35 | 3.1 | 0.051 |
|  | AOA2014 | bPb-3227 | 7H | 87.39 | 2.6 | 0.042 |
| wb in 2013 | TPC2013 | bPb-2723 | 1H | 11.54 | 2.55 | 0.046 |
|  | TPC2013 | bPb-9604 | 1H | 16.1 | 2.73 | 0.053 |
|  | TPC2013 | bPb-4531 | 1H | 60.21 | 3.12 | 0.058 |
|  | TPC2013 | bPb-3605 | 1H | 62.23 | 3.1 | 0.06 |
|  | TPC2013 | bPb-3050 | 2H | 30.24 | 2.59 | 0.045 |
|  | TPC2013 | bPb-0302 | 3H | 153.55 | 2.81 | 0.053 |
|  | TPC2013 | bPb-1829 | 3H | 155.84 | 2.9 | 0.052 |
|  | TPC2013 | bPb-1329 | 4H | 93.64 | 2.83 | 0.05 |
|  | TPC2013 | bPb-2419 | 5H | 188.32 | 2.73 | 0.049 |
|  | TPC2013 | bPb-3973 | 5H | 188.8 | 2.73 | 0.049 |
|  | TPC2013 | bPb-0572 | 6H | 17.86 | 3.56 | 0.072 |
|  | TPC2013 | bPb-5259 | 7H | 3.02 | 3.04 | 0.055 |
|  | TPC2013 | bPb-2595 | 7H | 8.26 | 2.66 | 0.048 |
|  | FLC2013 | bPb-3776 | 1H | 10.47 | 2.59 | 0.062 |
|  | FLC2013 | bPb-3605 | 1H | 62.23 | 2.52 | 0.058 |
|  | FLC2013 | bPb-8399 | 2H | 25.73 | 3.25 | 0.041 |
|  | FLC2013 | bPb-6879 | 2H | 26.03 | 3.22 | 0.086 |
|  | FLC2013 | bPb-0858 | 2H | 95.68 | 2.86 | 0.067 |
|  | FLC2013 | bPb-2587 | 2H | 146.34 | 3.92 | 0.095 |
|  | FLC2013 | bPb-8978 | 3H | 133.53 | 3.42 | 0.081 |
|  | FLC2013 | bPb-9599 | 3H | 149.85 | 2.69 | 0.061 |
|  | FLC2013 | bPb-2960 | 5H | 134.93 | 2.66 | 0.059 |
|  | FLC2013 | bPb-8150 | 6H | 28.84 | 2.56 | 0.059 |
|  | FLC2013 | bPb-3621 | 6H | 122.08 | 4.44 | 0.113 |
|  | FLC2013 | bPb-5403 | 7H | 159.05 | 3.24 | 0.075 |
|  | AOA2013 | bPb-4531 | 1H | 60.21 | 4.72 | 0.098 |
|  | AOA2013 | bPb-3605 | 1H | 62.23 | 4 | 0.085 |
|  | AOA2013 | bPb-3050 | 2H | 30.24 | 3.47 | 0.068 |
|  | AOA2013 | bPb-2587 | 2H | 146.34 | 3.1 | 0.058 |
|  | AOA2013 | bPb-2910 | 3H | 51.59 | 4.66 | 0.099 |
|  | AOA2013 | bPb-0302 | 3H | 153.55 | 4.03 | 0.083 |
|  | AOA2013 | bPb-1829 | 3H | 155.84 | 3.71 | 0.073 |
|  | AOA2013 | bPb-8913 | 3H | 34.49 | 3.31 | 0.065 |
|  | AOA2013 | bPb-0836 | 3H | 61.87 | 3.07 | 0.057 |
|  | AOA2013 | bPb-6878 | 3H | 35.93 | 3.05 | 0.059 |
|  | AOA2013 | bPb-1264 | 3H | 5.97 | 2.83 | 0.052 |
|  | AOA2013 | bPb-9746 | 3H | 54.8 | 2.78 | 0.053 |
|  | AOA2013 | bPb-4456 | 3H | 154.16 | 2.71 | 0.052 |
|  | AOA2013 | bPb-0040 | 3H | 72.18 | 2.65 | 0.05 |
|  | AOA2013 | bPb-1329 | 4H | 93.64 | 3.2 | 0.061 |
|  | AOA2013 | bPb-6367 | 5H | 157.35 | 4.71 | 0.096 |
|  | AOA2013 | bPb-2960 | 5H | 134.93 | 2.8 | 0.052 |
|  | AOA2013 | bPb-3138 | 5H | 166.59 | 2.75 | 0.05 |
|  | AOA2013 | bPb-0572 | 6H | 17.86 | 3.37 | 0.068 |
|  | AOA2013 | bPb-8347 | 6H | 73.44 | 2.85 | 0.053 |
|  | AOA2013 | bPb-3621 | 6H | 122.08 | 2.8 | 0.052 |
|  | AOA2013 | bPb-1009 | 6H | 13.83 | 2.8 | 0.051 |
|  | AOA2013 | bPb-8150 | 6H | 28.84 | 2.69 | 0.052 |
|  | AOA2013 | bPb-2595 | 7H | 8.26 | 3.41 | 0.068 |
|  | AOA2013 | bPb-5259 | 7H | 3.02 | 2.94 | 0.057 |
|  | AOA2013 | bPb-3020 | 7H | 159.21 | 2.69 | 0.05 |
|  | AOA2013 | bPb-6214 | 7H | 106.93 | 2.58 | 0.049 |
|  | AOA2013 | bPb-9601 | 7H | 42.67 | 2.56 | 0.046 |
|  | AOA2013 | bPb-1454 | 7H | 84.95 | 2.51 | 0.048 |
| cb in 2014 | TPC2014 | bPb-1068 | 3H | 66.16 | 3.61 | 0.257 |
|  | TPC2014 | bPb-9405 | 4H | 86.69 | 2.51 | 0.161 |
|  | TPC2014 | bPb-0639 | 7H | 115.56 | 2.55 | 0.166 |
|  | FLC2014 | bPb-1068 | 3H | 66.16 | 2.79 | 0.188 |
|  | FLC2014 | bPb-1609 | 3H | 140.29 | 2.51 | 0.169 |
|  | AOA2014 | bPb-4966 | 4H | 86.69 | 2.5 | 0.122 |
|  | AOA2014 | bPb-6576 | 4H | 90.22 | 2.5 | 0.122 |
| wb in 2014 | TPC2014 | bPb-4531 | 1H | 60.21 | 4.98 | 0.107 |
|  | TPC2014 | bPb-3605 | 1H | 62.23 | 5.1 | 0.115 |
|  | TPC2014 | bPb-0589 | 1H | 141.24 | 2.69 | 0.968 |
|  | TPC2014 | bPb-3050 | 2H | 30.24 | 3.64 | 0.072 |
|  | TPC2014 | bPb-1329 | 4H | 93.64 | 3.71 | 0.073 |
|  | TPC2014 | bPb-9299 | 6H | 14.35 | 2.66 | 0.049 |
|  | TPC2014 | bPb-0572 | 6H | 17.86 | 5.61 | 0.137 |
|  | TPC2014 | bPb-4778 | 6H | 17.86 | 2.61 | 0.051 |
|  | TPC2014 | bPb-5498 | 6H | 20.37 | 2.63 | 0.051 |
|  | TPC2014 | bPb-8150 | 6H | 28.84 | 2.52 | 0.048 |
|  | TPC2014 | bPb-5259 | 7H | 3.02 | 3.41 | 0.067 |
|  | TPC2014 | bPb-6752 | 7H | 11.19 | 2.59 | 0.055 |
|  | TPC2014 | bPb-0981 | 7H | 140.94 | 2.54 | 0.046 |
|  | TPC2014 | bPb-1631 | 7H | 140.94 | 2.52 | 0.045 |
|  | TPC2014 | bPb-7983 | 7H | 141.9 | 2.53 | 0.046 |
|  | FLC2014 | bPb-3605 | 1H | 62.23 | 2.65 | 0.07 |
|  | FLC2014 | bPb-4531 | 1H | 60.21 | 2.63 | 0.067 |
|  | FLC2014 | bPb-6367 | 5H | 157.35 | 2.6 | 0.066 |
|  | AOA2014 | bPb-4531 | 1H | 60.21 | 3.15 | 0.071 |
|  | AOA2014 | bPb-3050 | 2H | 30.24 | 2.75 | 0.059 |
|  | AOA2014 | bPb-1829 | 3H | 155.84 | 2.72 | 0.058 |
|  | AOA2014 | bPb-1329 | 4H | 93.64 | 3.06 | 0.067 |
|  | AOA2014 | bPb-2960 | 5H | 134.93 | 2.84 | 0.063 |
|  | AOA2014 | bPb-6367 | 5H | 157.35 | 2.59 | 0.055 |
|  | AOA2014 | bPb-2595 | 7H | 8.26 | 2.57 | 0.056 |
| wild in both years | AOA2013 | bPb-4531 | 1H | 60.21 | 4.72 | 0.098 |
| (P<0.001) | AOA2013 | bPb-3605 | 1H | 62.23 | 4 | 0.085 |
|  | AOA2014 | bPb-4531 | 1H | 60.21 | 3.15 | 0.071 |
|  | TPC2013 | bPb-4531 | 1H | 60.21 | 3.12 | 0.058 |
|  | TPC2013 | bPb-3605 | 1H | 62.23 | 3.1 | 0.06 |
|  | TPC2014 | bPb-3605 | 1H | 62.23 | 5.1 | 0.115 |
|  | TPC2014 | bPb-4531 | 1H | 60.21 | 4.98 | 0.107 |
|  | FLC2013 | bPb-8399 | 2H | 25.73 | 3.25 | 0.041 |
|  | FLC2013 | bPb-6879 | 2H | 26.03 | 3.22 | 0.086 |
|  | AOA2013 | bPb-3050 | 2H | 30.24 | 3.47 | 0.068 |
|  | AOA2013 | bPb-2587 | 2H | 146.34 | 3.1 | 0.058 |
|  | FLC2013 | bPb-2587 | 2H | 146.34 | 3.92 | 0.095 |
|  | TPC2014 | bPb-3050 | 2H | 30.24 | 3.64 | 0.072 |
|  | AOA2013 | bPb-2910 | 3H | 51.59 | 4.66 | 0.099 |
|  | AOA2013 | bPb-0302 | 3H | 153.55 | 4.03 | 0.083 |
|  | AOA2013 | bPb-1829 | 3H | 155.84 | 3.71 | 0.073 |
|  | AOA2013 | bPb-8913 | 3H | 34.49 | 3.31 | 0.065 |
|  | AOA2013 | bPb-0836 | 3H | 61.87 | 3.07 | 0.057 |
|  | AOA2013 | bPb-6878 | 3H | 35.93 | 3.05 | 0.059 |
|  | FLC2013 | bPb-8978 | 3H | 133.53 | 3.42 | 0.081 |
|  | AOA2013 | bPb-1329 | 4H | 93.64 | 3.2 | 0.061 |
|  | AOA2014 | bPb-1329 | 4H | 93.64 | 3.06 | 0.067 |
|  | TPC2014 | bPb-1329 | 4H | 93.64 | 3.71 | 0.073 |
|  | AOA2013 | bPb-6367 | 5H | 157.35 | 4.71 | 0.096 |
|  | FLC2013 | bPb-3621 | 6H | 122.08 | 4.44 | 0.113 |
|  | AOA2013 | bPb-0572 | 6H | 17.86 | 3.37 | 0.068 |
|  | TPC2013 | bPb-0572 | 6H | 17.86 | 3.56 | 0.072 |
|  | TPC2014 | bPb-0572 | 6H | 17.86 | 5.61 | 0.137 |
|  | AOA2013 | bPb-2595 | 7H | 8.26 | 3.41 | 0.068 |
|  | FLC2013 | bPb-5403 | 7H | 159.05 | 3.24 | 0.075 |
|  | TPC2013 | bPb-5259 | 7H | 3.02 | 3.04 | 0.055 |
|  | TPC2014 | bPb-5259 | 7H | 3.02 | 3.41 | 0.067 |

Note: These markers were identified using Q+K model with significance threshold as –log_10_(p)>2.5 or 3. R^2^ (marker) denotes the contribution of the marker for phenotypic variation.
