# Supplementary material for: Association mapping for total polyphenol content, total flavonoid content and antioxidant activity in barley
Source: BMC Genomics. 2018 Jan 25;19:81. doi: 10.1186/s12864-018-4483-6 (PMC5784657; doi:10.1186/s12864-018-4483-6)
Supplement: Supplementary file 11 — Protein sequences of HvUGT (HORVU1Hr1G020560) in barley and UGTs in Arabidopsis thaliana. (PDF 187 kb) [file 12864_2018_4483_MOESM11_ESM.pdf]

Figure S6

>HvUGT (HORVU1Hr1G020560)
MDVGFSTAGPLRIVICPWLAFGHLLPYLELAERLASRGHRVAFVSTPRNLARL
PPPASPCSVDLVALQLPRVDGLPEGAESTNDVPDEKRELHWKAFDGLAAPFAD
FLAAACADDGRRPHWIIADCFHHWAAAAALDHKVPCAVLLPTAAMLAAAPR
QQPLGSKPVEAAAAASVLGQAAAAVRLAVPRYERDDVAPAYADDCASGMSIA
QRWFLAKERCTVLAIRSCVEWEPETFPLVETLLGKPVVPLGLLPPSADGGRRR
AAGSSEDHVTLRWLEEQPPDSVVYIALGSEVPLSIEQVHELALGLELAGTRFL
WALRKPAGAVVGNNDDDTLPPGFRDRTRGHGLVTMGWVPQISILAHAAVGA
FLTHCGRNSLIEGLLFGHPLVMLPIFGDQGPNARQMEAKKVGLQVARDDDDG
SFDRHGVAAAVRAVMVDGEARRGFVANALKMQAIVADKERHERYIDGFVQQ
LRSYLQATDDLTTATPNSS*
>AT5G49690
MVDKREEVMHVAMFPWLAMGHLLPFLRLSKLLAQKGHKISFISTPRNIERLP
KLQSNLASSITFVSFPLPPISGLPPSSESSMDVPYNKQQSLKAAFDLLQPPLKEF
LRRSSPDWIIYDYASHWLPSIAAELGISKAFFSLFNAATLCFMGPSSSLIEEIRST
PEDFTVVPPWVPFKSNIVFRYHEVTRYVEKTEEDVTGVSDSVRFGYSIDESDA
VFVRSCPEFEPEWFGLLKDLYRKPVFPIGFLPPVIEDDDAVDTTWVRIKKWLD
KQRLNSVVYVSLGTEASLRHEEVTELALGLEKSETPFFWVLRNEPKIPDGFKT
RVKGRGMVHVGWVPQVKILSHESVGGFLTHCGWNSVVEGLGFGKVPIFFPVL
NEQGLNTRLLHGKGLGVEVSRDERDGSFDSDSVADSIRLVMIDDAGEEIRAKA
KVMKDLFGNMDENIRYVDELVRFMRSKGSSSSS*
>AT2G22590
MTNFKDNDGDGTKLHVVMFPWLAFGHMVPYLELSKLIAQKGHKVSFISTPR
NIDRLLPRLPENLSSVINFVKLSLPVGDNKLPEDGEATTDVPFELIPYLKIAYDG
LKVPVTEFLESSKPDWVLQDFAGFWLPPISRRLGIKTGFFSAFNGATLGILKPP
GFEEYRTSPADFMKPPKWVPFETSVAFKLFECRFIFKGFMAETTEGNVPDIHRV
GGVIDGCDVIFVRSCYEYEAEWLGLTQELHRKPVIPVGVLPPKPDEKFEDTDT
WLSVKKWLDSRKSKSIVYVAFGSEAKPSQTELNEIALGLELSGLPFFWVLKTR
RGPWDTEPVELPEGFEERTADRGMVWRGWVEQLRTLSHDSIGLVLTHPGWG
TIIEAIRFAKPMAMLVFVYDQGLNARVIEEKKIGYMIPRDETEGFFTKESVANS
LRLVMVEEEGKVYRENVKEMKGVFGDMDRQDRYVDSFLEYLVTNR*
>AT5G65550
MAEPKPKLHVAVFPWLALGHMIPYLQLSKLIARKGHTVSFISTARNISRLPNISS
DLSVNFVSLPLSQTVDHLPENAEATTDVPETHIAYLKKAFDGLSEAFTEFLEAS
KPNWIVYDILHHWVPPIAEKLGVRRAIFCTFNAASIIIIGGPASVMIQGHDPRKT
AEDLIVPPPWVPFETNIVYRLFEAKRIMEYPTAGVTGVELNDNCRLGLAYVGS
EVIVIRSCMELEPEWIQLLSKLQGKPVIPIGLLPATPMDDADDEGTWLDIREWL
DRHQAKSVVYVALGTEVTISNEEIQGLAHGLELCRLPFFWTLRKRTRASMLLP
DGFKERVKERGVIWTEWVPQTKILSHGSVGGFVTHCGWGSAVEGLSFGVPLI
MFPCNLDQPLVARLLSGMNIGLEIPRNERDGLFTSASVAETIRHVVVEEEGKIY
RNNAASQQKKIFGNKRLQDQYADGFIEFLENPIAGV*
>AT5G26310

MHITKPHAAMFSSPGMGHVLPVIELAKRLSANHGFHVTVFVLETDAASVQSK
LLNSTGVDIVNLPSPDISGLVDPNAHVVTKIGVIMREAVPTLRSKIVAMHQNPT
ALIIDLFGTDALCLAAELNMLTYVFIASNARYLGVSIYYPTLDEVIKEEHTVQR
KPLTIPGCEPVRFEDIMDAYLVPDEPVYHDLVRHCLAYPKADGILVNTWEEME
PKSLKSLQDPKLLGRVARVPVYPVGPLCRPIQSSTTDHPVFDWLNKQPNESVL
YISFGSGGSLTAQQLTELAWGLEESQQRFIWVVRPPVDGSSCSDYFSAKGGVT
KDNTPEYLPEGFVTRTCDRGFMIPSWAPQAEILAHQAVGGFLTHCGWSSTLES
VLCGVPMIAWPLFAEQNMNAALLSDELGISVRVDDPKEAISRSKIEAMVRKV
MAEDEGEEMRRKVKKLRDTAEMSLSIHGGGSAHESLCRVTKECQRFLECVG
DLGRGA*
>AT2G18570
MDQPHALLVASPGLGHLIPILELGNRLSSVLNIHVTILAVTSGSSSPTETEAIHA
AAARTICQITEIPSVDVDNLVEPDATIFTKMVVKMRAMKPAVRDAVKLMKRK
PTVMIVDFLGTELMSVADDVGMTAKYVYVPTHAWFLAVMVYLPVLDTVVEG
EYVDIKEPLKIPGCKPVGPKELMETMLDRSGQQYKECVRAGLEVPMSDGVLV
NTWEELQGNTLAALREDEELSRVMKVPVYPIGPIVRTNQHVDKPNSIFEWLDE
QRERSVVFVCLGSGGTLTFEQTVELALGLELSGQRFVWVLRRPASYLGAISSD
DEQVSASLPEGFLDRTRGVGIVVTQWAPQVEILSHRSIGGFLSHCGWSSALESL
TKGVPIIAWPLYAEQWMNATLLTEEIGVAVRTSELPSERVIGREEVASLVRKIMA
EEDEEGQKIRAKAEEVRVSSERAWSKDGSSYNSLFEWAKRCYLVP*
>AT2G18560
MREMKSTVRDAVKSMKQKPTVMIVDFFGTALLSITDVGVTSKYVYIPSHAWF
LALIVYLPVLDKVMEGEYVDIKEPMKIPGCKPVGPKELLDTMLDRSDQQYRD
CVQIGLEIPMSDGVLVNTWGELQGKTLAALREDIDLNRVIKVPVYPIGPIVRTN
VLIEKPNSTFEWLDKQEERSVVYVCLGSGGTLSFEQTMELAWGLELSCQSFL
WVLRKPPSYLGASSKDDDQVSDGLPEGFLDRTRGVGLVVTQWAPQVEILSHR
SIGGFLSHCGWSSVLESLTKGVPIIAWPLYAEQWMNATLLTEEIGMAIRTSELPS
KKVISREEVASLVKKIVAEEDKEGRKIKTKAEEVRVSSERAWTHGGSSHSSLFE
WAKRCGLVS*
>AT4G36770
MELHGALVASPGMGHAVPILELGKHLLNHHGFDRVTVFLVTDDVSRSKSLIGK
TLMEEDPKFVIRFIPLDVSGQDLSGSLLTKLAEMMRKALPEIKSSVMELEPRPR
VFVVDLLGTEALEVAKELGIMRKHVLVTTSAWFLAFTVYMASLDKQELYKQL
SSIGALLIPGCSPVKFERAQDPRKYIRELAESQRIGDEVITADGVFVNTWHSLE
QVTIGSFLDPENLGRVMRGVPVYPVGPLVRPAEPGLKHGVLDWLDLQPKESV
VYVSFGSGGALTFEQTNELAYGLELTGHRFVWVVRPPAEDDPSASMFDKTKN
ETEPLDFLPNGFLDRTKDIGLVVRTWAPQEEILAHKSTGGFVTHCGWNSVLESI
VNGVPMVAWPLYSEQKMNARMVSGELKIALQINVADGIVKKEVIAEMVKRV
MDEEEGKEMRKNVKELKKTAEEALNMTHIPSAYFT*
>AT4G01070
MEESKTPHVAIIPSPGMGHLIPLVEFAKRLVHLHGLTVTFVIAGEGPPSKAQRTV
LDSLPSSISSVFLPPVDLTDLSSSTRIESRISLTVTRSNPELRKVFDSFVEGGRLPT
ALVVDLFGTDAFDVAVEFHVPPYIFYPTTANVLSFFLHLPKLDETVSCEFRELT
EPLMLPGCVPVAGKDFLDPAQDRKDDAYKWLLHNTKRYKEAEGILVNTFFEL

EPNAIKALQEPGLDKPPVYPVGPLVNIGKQEAKQTEESECLKWLDNQPLGSVL
YVSFGSGGTLTCEQLNELALGLADSEQRFLWVIRSPSGIANSSYFDSHSQTDPL
TFLPPGFLERTKKRGFVIPFWAPQAQVLAHPSTGGFLTHCGWNSTLESVVSGIP
LIAWPLYAEQKMNAVLLSEDIRAALRPRAGDDGLVRREEVARVVKGLMEGEE
GKGVRNKMKELKEAACRVLKDDGTSTKALSLVALKWKAHKKELEQNGNH*
>AT5G66690
MHITKPHAAMFSSPGMGHVIPVIELGKRLSANNGFHVTVFVLETDAASAQSK
FLNSTGVDIVKLPSPDIYGLVDPDDHVVTKIGVIMRAAVPALRSKIAAMHQKP
TALIVDLFGTDALCLAKEFNMLSYVFIPTNARFLGVSIYYPNLDKDIKEEHTVQ
RNPLAIPGCEPVRFEDTLDAYLVPDEPVYRDFVRHGLAYPKADGILVNTWEEM
EPKSLKSLLNPKLLGRVARVPVYPIGPLCRPIQSSETDHPVLDWLNEQPNESVL
YISFGSGGCLSAKQLTELAWGLEQSQQRFVWVVRPPVDGSCCSEYVSANGGG
TEDNTPEYLPEGFVSRTSDRGFVVPSWAPQAEILSHRAVGGFLTHCGWSSTLES
VVGGVPMIAWPLFAEQNMNAALLSDELGIAVRLDDPKEDISRWKIEALVRKV
MTEKEGEAMRRKVKKLRDSAEMSLSIDGGGLAHESLCRVTKECQRFLERVV
DLSRGA*
>AT4G34138
MGTPVEVSKLHFLLFPFMAHGHMIPTLDMAKLFATKGAKSTILTTPLNAKLFF
EKPIKSFNQDNPGLEDITIQILNFPCTELGLPDGCENTDFIFSTPDLNVGDLSQK
FLLAMKYFEEPLEELLVTMRPDCLVGNMFFPWSTKVAEKFGVPRLVFHGTGY
FSLCASHCIRLPKNVATSSEPFVIPDLPGDILITEEQVMETEEESVMGRFMKAIR
DSERDSFGVLVNSFYELEQAYSDYFKSFVAKRAWHIGPLSLGNRKFEEKAERG
KKASIDEHECLKWLDSKKCDSVIYMAFGTMSSFKNEQLIEIAAGLDMSGHDF
VWVVNRKGSQVEKEDWLPEGFEEKTKGKGLIIRGWAPQVLILEHKAIGGFLT
HCGWNSLLEGVAAGLPMVTWPVGAEQFYNEKLVTQVLKTGVSVGVKKMM
QVVGDFISREKVEGAVREVMVGEERRKRAKELAEMAKNAVKEGGSSDLEVD
RLMEELTLVKLQKEKV*
>AT3G50740
MKITKPHVAMFASPGMGHIIPVIELGKRLAGSHGFDVTIFVLETDAASAQSQFL
NSPGCDAALVDIVGLPTPDISGLVDPSAFFGIKLLVMMRETIPTIRSKIEEMQHK
PTALIVDLFGLDAIPLGGEFNMLTYIFIASNARFLAVALFFPTLDKDMEEEHIIK
KQPMVMPGCEPVRFEDTLETFLDPNSQLYREFVPFGSVFPTCDGIIVNTWDDM
EPKTLKSLQDPKLLGRIAGVPVYPIGPLSRPVDPSKTNHPVLDWLNKQPDESV
LYISFGSGGSLSAKQLTELAWGLEMSQQRFVWVVRPPVDGSACSAYLSANSG
KIRDGTPDYLPEGFVSRTHERGFMVSSWAPQAEILAHQAVGGFLTHCGWNSIL
ESVVGGVPMIAWPLFAEQMMNATLLNEELGVAVRSKKLPSEGVITRAEIEALV
RKIMVEEEGAEMRKKIKKLKETAAESLSCDGGVAHESLSRIADESEHLLERVR
CMARGA*
>AT4G34131
MSSDPHRKLHVVFFPFMAYGHMIPTLDMAKLFSSRGAKSTILTTPLNSKIFQKP
IERFKNLNPSFEIDIQIFDFPCVDLGLPEGCENVDFFTSNNNDDRQYLTLKFFKS
TRFFKDQLEKLLETTRPDCLIADMFFPWATEAAEKFNVPRLVFHGTGYFSLCS
EYCIRVHNPQNIVASRYEPFVIPDLPGNIVITQEQIADRDEESEMGKFMIEVKES
DVKSSGVIVNSFYELEPDYADFYKSVVLKRAWHIGPLSVYNRGFEEKAERGK

KASINEVECLKWLDSKKPDSVIYISFGSVACFKNEQLFEIAAGLETSGANFIWV
VRKNIGIEKEEWLPEGFEERVKGKGMIIRGWAPQVLILDHQATCGFVTHCGW
NSLLEGVAAGLPMVTWPVAAEQFYNEKLVTQVLRTGVSVGAKKNVRTTGDFI
SREKVVKAVREVLVGEEADERRERAKKLAEMAKAAVEGGSSFNDLNSFIEEF
TS*
>AT1G01420
MADGNTPHVAIIPSPGIGHLIPLVELAKRLLDNHGFTVTFIIPGDSPPSKAQRSV
LNSLPSSIASVFLPPADLSDVPSTARIETRISLTVTRSNPALRELFGSLSAEKRLPA
VLVVDLFGTDAFDVAAEFHVSPYIFYASNANVLTFLLHLPKLDETVSCEFRELT
EPVIIPGCVPITGKDFVDPCQDRKDESYKWLLHNVKRFKEAEGILVNSFVDLEP
NTIKIVQEPAPDKPPVYLIGPLVNSGSHDADVNDEYKCLNWLDNQPFGSVLYV
SFGSGGTLTFEQFIELALGLAESGKRFLWVIRSPSGIASSSYFNPQSRNDPFSFLP
QGFLDRTKEKGLVVGSWAPQAQILTHTSIGGFLTHCGWNSSLESIVNGVPLIA
WPLYAEQKMNALLLVDVGAALRARLGEDGVVGREEVARVVKGLIEGEEGNA
VRKKMKELKEGSVRVLRDDGFSTKSLNEVSLKWKAHQRKIDQEQESFL*
>AT4G27570
MGGLKFHVLMYPWFATGHMTPFLFLANKLAEKGHTVTFLLPKKSLKQLEHF
NLFPHNIVFRSVTVPHVDGLPVGTETASEIPVTSTDLLMSAMDLTRDQVEAVV
RAVEPDLIFFDFAHWIPEVARDFGLKTVKYVVVSASTIASMLVPGGELGVPPPG
YPSSKVLLRKQDAYTMKKLEPTNTIDVGPNLLERVTTSLMNSDVIAIRTAREIE
GNFCDYIEKHCRKKVLLTGPVFPEPDKTRELEERWVKWLSGYEPDSVVFCAL
GSQVILEKDQFQELCLGMELTGSPFLVAVKPPRGSSTIQEALPEGFEERVKGRG
LVWGGWVQQPLILSHPSVGCFVSHCGFGSMWESLLSDCQIVLVPQLGDQVLN
TRLLSDELKVSVEVAREETGWFSKESLCDAVNSVMKRDSELGNLVRKNHTK
WRETVASPGLMTGYVDAFVESLQDLVSGTTHD*
>AT2G29740
MAKQQEAELIFIPFPIPGHILATIELAKRLISHQPSRIHTITILHWSLPFLPQSDTIA
FLKSLIETESRIRLITLPDVQNPPPMELFVKASESYILEYVKKMVPLVRNALSTL
LSSRDESDSVHVAGLVLDFFCVPLIDVGNEFNLPSYIFLTCSASFLGMMKYLLE
RNRETKPELNRSSDEETISVPGFVNSVPVKVLPPGLFTTESYEAWVEMAERFPE
AKGILVNSFESLERNAFDYFDRRPDNYPPVYPIGPILCSNDRPNLDLSERDRILK
WLDDQPESSVVFLCFGSLKSLAASQIKEIAQALELVGIRFLWSIRTDPKEYASP
NEILPDGFMNRVMGLGLVCGWAPQVEILAHKAIGGFVSHCGWNSILESLRFG
VPIATWPMYAEQQLNAFTIVKELGLALEMRLDYVSEYGEIVKADEIAGAVRSL
MDGEDVPRRKLKEIAEAGKEAVMDGGSSFVAVKRFIDGL*
>AT2G15490
MNREQIHILFFPFMAHGHMIPLLDMAKLFARRGAKSTLLTTPINAKILEKPIEA
FKVQNPDLEIGIKILNFPCVELGLPEGCENRDFINSYQKSDSFDLFLKFLFSTKY
MKQQLESFIETTKPSALVADMFFPWATESAEKIGVPRLVFHGTSSFALCCSYNM
RIHKPHKKVASSSTPFVIPGLPGDIVITEDQANVTNEETPFGKFWKEVRESETSS
FGVLVNSFYELESSYADFYRSFVAKKAWHIGPLSLSNRGIAEKAGRGKKANID
EQECLKWLDSKTPGSVVYLSFGSGTGLPNEQLLEIAFGLEGSGQNFIWVVSKN
ENQVGTGENEDWLPKGFEERNKGKGLIIRGWAPQVLILDHKAIGGFVTHCGW
NSTLEGIAAGLPMVTWPMGAEQFYNEKLLTKVLRIGVNVGATELVKKGKLIS

RAQVEKAVREVIGGEKAEERRLRAKELGEMAKAAVEEGGSSYNDVNKFMEE
LNGRK*
>AT2G29730
MRNVELIFIPTPTVGHLVPFLEFARRLIEQDDRIRITILLMKLQGQSHLDTYVKS
IASSQPFVRFIDVPELEEKPTLGSTQSVEAYVYDVIERNIPLVRNIVMDILTSLAL
DGVKVKGLVVDFFCLPMIDVAKDISLPFYVFLTTNSGFLAMMQYLADRHSRD
TSVFVRNSEEMLSIPGFVNPVPANVLPSALFVEDGYDAYVKLAILFTKANGILV
NSSFDIEPYSVNHFLQEQNYPSVYAVGPIFDLKAQPHPEQDLTRRDELMKWLD
DQPEASVVFLCFGSMARLRGSLVKEIAHGLELCQYRFLWSLRKEEVTKDDLP
EGFLDRVDGRGMICGWSPQVEILAHKAVGGFVSHCGWNSIVESLWFGVPIVT
WPMYAEQQLNAFLMVKELKLAVELKLDYRVHSDEIVNANEIETAIRYVMDTD
NNVVRKRVMDISQMIQRATKNGGSSFAAIEKFIYDVIGIKP*
>AT1G07260
MKAEAEIIFVTYPSPGHLLVSIEFAKSLIKRDDRIHTITILYWALPLAPQAHLFAK
SLVASQPRIRLLALPDVQNPPPLELFFKAPEAYILESTKKTVPLVRDALSTLVSS
RKESGSVRVVGLVIDFFCVPMIEVANELNLPSYIFLTCNAGFLSMMKYLPERHR
ITTSELDLSSGNVEHPIPGYVCSVPTKVLPPGLFVRESYEAWVEIAEKFPGAKGI
LVNSVTCLEQNAFDYFARLDENYPPVYPVGPVLSLKDRPSPNLDASDRDRIMR
WLEDQPESSIVYICFGSLGIIGKLQIEEIAEALELTGHRFLWSIRTNPTEKASPYD
LLPEGFLDRTASKGLVCDWAPQVEVLAHKALGGFVSHCGWNSVLESLWFGV
PIATWPMYAEQQLNAFSMVKELGLAVELRLDYVSAYGEIVKAEEIAGAIRSLM
DGEDTPRKRVKEMAEAARNALMDGGSSFVAVKRFLDELIGGDV*
>AT1G07250
MVKETELIFIPVPSTGHILVHIEFAKRLINLDHRIHTITILNLSSPSSPHASVFARS
LIASQPKIRLHDLPPIQDPPPFDLYQRAPEAYIVKLIKKNTPLIKDAVSSIVASRR
GGSDSVQVAGLVLDLFCNSLVKDVGNELNLPSYIYLTCNARYLGMMKYIPDR
HRKIASEFDLSSGDEELPVPGFINAIPTKFMPPGLFNKEAYEAYVELAPRFADA
KGILVNSFTELEPHPFDYFSHLEKFPPVYPVGPILSLKDRASPNEEAVDRDQIVG
WLDDQPESSVVFLCFGSRGSVDEPQVKEIARALELVGCRFLWSIRTSGDVETN
PNDVLPEGFMGRVAGRGLVCGWAPQVEVLAHKAIGGFVSHCGWNSTLESLW
FGVPVATWPMYAEQQLNAFTLVKELGLAVDLRMDYVSSRGGLVTCDEIARAV
RSLMDGGDEKRKKVKEMADAARKALMDGGSSSLATARFIAELFEDGSSC*
>AT3G21790
MKFELVFIPYPGIGHLRSTVEMAKLLVDRETRLSISVIILPFISEGEVGASDYIAA
LSASSNNRLRYEVISAVDQPTIEMTTIEIHMKNQEPKVRSTVAKLLEDYSSKPD
SPKIAGFVLDMFCTSMVDVANEFGFPSYMFYTSSAGILSVTYHVQMLCDENK
YDVSENDYADSEAVLNFPSLSRPYPVKCLPHALAANMWLPVFVNQARKFRE
MKGILVNTVAELEPYVLKFLSSSDTPPVYPVGPLLHLENQRDDSKDEKRLEIIR
WLDQQPPSSVVFLCFGSMGGFGEEQVREIAIALERSGHRFLWSLRRASPNIFKE
LPGEFTNLEEVLPEGFFDRTKDIGKVIGWAPQVAVLANPAIGGFVTHCGWNST
LESLWFGVPTAAWPLYAEQKFNAFLMVEELGLAVEIRKYWRGEHLAGLPTAT
VTAEEIEKAIMCLMEQDSDVRKRVKDMSEKCHVALMDGGSSRTALQKFIEEV
AKNIVSLDKEFEHVALK*
>AT3G21800

MNKFALVFVPFPILGHLKSTAEMAKLLVEQETRLSISIIILPLLSGDDVSASAYIS
ALSAASNDRLHYEVISDGDQPTVGLHVDNHIPMVKRTVAKLVDDYSRRPDSP
RLAGLVVDMFCISVIDVANEVSVPCYLFYTSNVGILALGLHIQMLFDKKEYSV
SETDFEDSEVVLDVPSLTCPYPVKCLPYGLATKEWLPMYLNQGRRFREMKGIL
VNTFAELEPYALESLHSSGDTPRAYPVGPLLHLENHVDGSKDEKGSDILRWLD
EQPPKSVVFLCFGSIGGFNEEQAREMAIALERSGHRFLWSLRRASRDIDKELPG
EFKNLEEILPEGFFDRTKDKGKVIGWAPQVAVLAKPAIGGFVTHCGWNSILESL
WFGVPIAPWPLYAEQKFNAFVMVEELGLAVKIRKYWRGDQLVGTATVIVTAE
EIERGIRCLMEQDSDVRNRVKEMSKKCHMALKDGGSSQSALKLFIQDVTKYI
A*
>AT2G15480
MNREVSERIHILFFPFMAQGHMIPILDMAKLFSRRGAKSTLLTTPINAKIFEKPI
EAFKNQNPDLEIGIKIFNFPCVELGLPEGCENADFINSYQKSDSGDLFLKFLFST
KYMKQQLESFIETTKPSALVADMFFPWATESAEKLGVPRLVFHGTSFFSLCCSY
NMRIHKPHKKVATSSTPFVIPGLPGDIVITEDQANVAKEETPMGKFMKEVRESE
TNSFGVLVNSFYELESAYADFYRSFVAKRAWHIGPLSLSNRELGEKARRGKKA
NIDEQECLKWLDSKTPGSVVYLSFGSGTNFTNDQLLEIAFGLEGSGQSFIWVV
RKNENQGDNEEWLPEGFKERTTGKGLIIPGWAPQVLILDHKAIGGFVTHCGW
NSAIEGIAAGLPMVTWPMGAEQFYNEKLLTKVLRIGVNVGATELVKKGKLISR
AQVEKAVREVIGGEKAEERRLWAKKLGEMAKAAVEEGGSSYNDVNKFMEEL
NGRK*
>AT5G53990
MGQNFHAFMFPWFAFGHMTPYLHLANKLAAKGHRVTFLLPKKAQKQLEHH
NLFPDRIIFHSLTIPHVDGLPAGAETASDIPISLGKFLTAAMDLTRDQVEAAVRA
LRPDLIFFDTAYWVPEMAKEHRVKSVIYFVISANSIAHELVPGGELGVPPPGYP
SSKVLYRGHDAHALLTFSIFYERLHYRITTGLKNCDFISIRTCKEIEGKFCDYIE
RQYQRKVLLTGPMLPEPDNSRPLEDRWNHWLNQFKPGSVIYCALGSQITLEK
DQFQELCLGMELTGLPFLVAVKPPKGAKTIQEALPEGFEERVKNHGVVWGEW
VQQPLILAHPSVGCFVTHCGFGSMWESLVSDCQIVLLPYLCDQILNTRLMSEE
LEVSVEVKREETGWFSKESLSVAITSVMDKDSELGNLVRRNHAKLKEVLVSP
GLLTGYTDEFVETLQNIVNDTNLE* >AT5G54010
MGSKFHAFMFPWFGFGHMTAFLHLANKLAEKDHKITFLLPKKARKQLESLNL
FPDCIVFQTLTIPSVDGLPDGAETTSDIPISLGSFLASAMDRTRIQVKEAVSVGK
PDLIFFDFAHWIPEIAREYGVKSVNFITISAACVAISFVPGRSQDDLGSTPPGYPS
SKVLLRGHETNSLSFLSYPFGDGTSFYERIMIGLKNCDVISIRTCQEMEGKFCD
FIENQFQRKVLLTGPMLPEPDNSKPLEDQWRQWLSKFDPGSVIYCALGSQIILE
KDQFQELCLGMELTGLPFLVAVKPPKGSSTIQEALPKGFEERVKARGVVWGG
WVQQPLILAHPSIGCFVSHCGFGSMWEALVNDCQIVFIPHLGEQILNTRLMSE
ELKVSVEVKREETGWFSKESLSGAVRSVMDRDSELGNWARRNHVKWKESLL
RHGLMSGYLNKFVEALEKLVQNINLE*
>AT4G15260
MFCSSMIDIANEFGVPCYMIYTSNATFLGITLHVQEMYDDKKYDVSDLDESV
NELEFPCLTRPYPVKCLPHILSSKDWLPFFAAQGRSFRKMKGILVNTVAELEPH
ALKMFNNVDLPQAYPVGPVLHLDNGDDDDEKRLEVLRWLDDQPPKSVLFLC

FGSMGGFTEEQTREVAVALNRSGHRFLWSLRRASPNIMMERPGDYKNLEEVL
PDGFLERTLDRGKVIGWAPQVAVLEKPAIGGFVTHCGWNSMLESLWFGVPMV
TWPLYAEQKVNAFEMVEELGLAVEIRKCISGDLLLIGEMEIVTAEDIERAIRCV
MEQDSDVRSRVKEMAEKCHVALMDGGSSKTALQKFIQDVIENVA*
>AT2G29710
MRNAELIFIPTPTVGHLVPFLEFARRLIEQDDRIRITFLLMKQQGQSHLDSYVK
TISSSLPFVRFIDVPELEEKPTLGTQSVEAYVYDFIETNVPLVQNIIMGILSSPAF
DGVTVKGFVADFFCLPMIDVAKDASLPFYVFLTSNSGFLAMMQYLAYGHKKD
TSVFARNSEEMLSIPGFVNPVPAKVLPSALFIEDGYDADVKLAILFTKANGILV
NTSFDIEPTSLNHFLGEENYPSVYAVGPIFNPKAHPHPDQDLACCDESMKWLD
AQPEASVVFLCFGSMGSLRGPLVKEIAHGLELCQYRFLWSLRTEEVTNDDLLP
EGFMDRVSGRGMICGWSPQVEILAHKAVGGFVSHCGWNSIVESLWFGVPIVT
WPMYAEQQLNAFLMVKELKLAVELKLDYSVHSGEIVSANEIETAISCVMNKD
NNVVRKRVMDISQMIQRATKNGGSSFAAIEKFIHDVIGTRT*
>AT4G34135
MGSDHHHRKLHVMFFPFMAYGHMIPTLDMAKLFSSRGAKSTILTTSLNSKILQ
KPIDTFKNLNPGLEIDIQIFNFPCVELGLPEGCENVDFFTSNNNDDKNEMIVKF
FFSTRFFKDQLEKLLGTTRPDCLIADMFFPWATEAAGKFNVPRLVFHGTGYFS
LCAGYCIGVHKPQKRVASSSEPFVIPELPGNIVITEEQIIDGDGESDMGKFMTEV
RESEVKSSGVVLNSFYELEHDYADFYKSCVQKRAWHIGPLSVYNRGFEEKAE
RGKKANIDEAECLKWLDSKKPNSVIYVSFGSVAFFKNEQLFEIAAGLEASGTS
FIWVVRKTKDDREEWLPEGFEERVKGKGMIIRGWAPQVLILDHQATGGFVTH
CGWNSLLEGVAAGLPMVTWPVGAEQFYNEKLVTQVLRTGVSVGASKHMKV
MMGDFISREKVDKAVREVLAGEAAEERRRRAKKLAAMAKAAVEEGGSSFND
LNSFMEEFSS*
>AT4G27560
MGGLKFHVLMYPWFATGHMTPFLFLANKLAEKGHTVTFLIPKKALKQLENL
NLFPHNIVFRSVTVPHVDGLPVGTETVSEIPVTSADLLMSAMDLTRDQVEGVV
RAVEPDLIFFDFAHWIPEVARDFGLKTVKYVVVSASTIASMLVPGGELGVPPPG
YPSSKVLLRKQDAYTMKNLESTNTINVGPNLLERVTTSLMNSDVIAIRTAREIE
GNFCDYIEKHCRKKVLLTGPVFPEPDKTRELEERWVKWLSGYEPDSVVFCAL
GSQVILEKDQFQELCLGMELTGSPFLVAVKPPRGSSTIQEALPEGFEERVKGRG
VVWGEWVQQPLLLSHPSVGCFVSHCGFGSMWESLLSDCQIVLVPQLGDQVL
NTRLLSDELKVSVEVAREETGWFSKESLFDAINSVMKRDSEIGNLVKKNHTK
WRETLTSPGLVTGYVDNFIESLQDLVSGTNHVSK*
>AT1G01390
MAEANTPHIAIMPSPGMGHLIPFVELAKRLVQHDCFTVTMIISGETSPSKAQRS
VLNSLPSSIASVFLPPADLSDVPSTARIETRAMLTMTRSNPALRELFGSLSTKKS
LPAVLVVDMFGADAFDVAVDFHVSPYIFYASNANVLSFFLHLPKLDKTVSCEF
RYLTEPLKIPGCVPITGKDFLDTVQDRNDDAYKLLLHNTKRYKEAKGILVNSF
VDLESNAIKALQEPAPDKPTVYPIGPLVNTSSSNVNLEDKFGCLSWLDNQPFG
SVLYISFGSGGTLTCEQFNELAIGLAESGKRFIWVIRSPSEIVSSSYFNPHSETDP
FSFLPIGFLDRTKEKGLVVPSWAPQVQILAHPSTCGFLTHCGWNSTLESIVNGV
PLIAWPLFAEQKMNTLLLVEDVGAALRIHAGEDGIVRREEVVRVVKALMEGE

EGKAIGNKVKELKEGVVRVLGDDGLSSKSFGEVLLKWKTHQRDINQETSH*
>AT4G15280
MKIELVFIPLPGIGHLRPTVKLAKQLIGSENRLSITIIIIPSRFDAGDASACIASLTT
LSQDDRLHYESISVAKQPPTSDPDPVPAQVVYIEKQKTKVRDAVAARIVDPTRKL
AGFVVDMFCSSMIDVANEFGVPCYMVYTSNATFLGTMLHVQQMYDQKKYD
VSELENSVTELEFPSLTRPYPVKCLPHILTSKEWLPLSLAQARCFRKMKGILVN
TVAELEPHALKMFNINGDDLPQVYPVGPVLHLENGNDDDEKQSEILRWLDEQ
PSKSVVFLCFGSLGGFTEEQTRETAVALDRSGQRFLWCLRHASPNIKTDRPRD
YTNLEEVLPEGFLERTLDRGKVIGWAPQVAVLEKPAIGGFVTHCGWNSILESL
WFGVPMVTWPLYAEQKVNAFEMVEELGLAVEIRKYLKGDLFAGEMETVTAE
DIERAIRRVMEQDSDVRNNVKEMAEKCHFALMDGGSSKAALEKFIQDVIENM
D*
>AT3G21780
MKIELVFIPSPAISHLMATVEMAEQLVDKNDNLSITVIIISFSSKNTSMITSLTSN
NRLRYEIISGGDQQPTELKATDSHIQSLKPLVRDAVAKLVDSTLPDAPRLAGFV
VDMYCTSMIDVANEFGVPSYLFYTSNAGFLGLLLHIQFMYDAEDIYDMSELE
DSDVELVVPSLTSPYPLKCLPYIFKSKEWLTFFVTQARRFRETKGILVNTVPDL
EPQALTFLSNGNIPRAYPVGPLLHLKNVNCDYVDKKQSEILRWLDEQPPRSVV
FLCFGSMGGFSEEQVRETALALDRSGHRFLWSLRRASPNILREPPGEFTNLEEI
LPEGFFDRTANRGKVIGWAEQVAILAKPAIGGFVSHGGWNSTLESLWFGVPM
AIWPLYAEQKFNAFEMVEELGLAVEIKKHWRGDLLLGRSEIVTAEEIEKGIICL
MEQDSDVRKRVNEISEKCHVALMDGGSSETALKRFIQDVTENIAWSETES*
>AT1G07240
MKTAELIFVPLPETGHLLSTIEFGKRLLNLDRRISMITILSMNLPYAPHADASLA
SLTASEPGIRIISLPEIHDPPPIKLLDTSSETYILDFIHKNIPCLRKTIQDLVSSSSSS
GGGSSHVAGLILDFFCVGLIDIGREVNLPSYIFMTSNFGFLGVLQYLPERQRLTP
SEFDESSGEEELHIPAFVNRVPAKVLPPGVFDKLSYGSLVKIGERLHEAKGILVN
SFTQVEPYAAEHFSQGRDYPHVYPVGPVLNLTGRTNPGLASAQYKEMMKWL
DEQPDSSVLFLCFGSMGVFPAPQITEIAHALELIGCRFIWAIRTNMAGDGDPQE
PLPEGFVDRTMGRGIVCSWAPQVDILAHKATGGFVSHCGWNSVQESLWYGVP
IATWPMYAEQQLNAFEMVKELGLAVEIRLDYVADGDRVTLEIVSADEIATAVR
SLMDSDNPVRKKVIEKSSVARKAVGDGGSSTVATCNFIKDILGDHF*
>AT3G16520
MGEEAIVLYPAPPIGHLVSMVELGKTILSKNPSLSIHIILVPPPYQPESTATYISSV
SSSFPSITFHHLPAVTPYSSSSTSRHHHESLLLEILCFSNPSVHRTLFSLSRNFNVR
AMIIDFFCTAVLDITADFTFPVYFFYTSGAACLAFSFYLPTIDETTPGKNLKDIPT
VHIPGVPPMKGSDMPKAVLERDDEVYDVFIMFGKQLSKSSGIIINTFDALENR
AIKAITEELCFRNIYPIGPLIVNGRIEDRNDNKAVSCLNWLDSQPEKSVVFLCFG
SLGLFSKEQVIEIAVGLEKSGQRFLWVVRNPPELEKTELDLKSLLPEGFLSRTE
DKGMVVKSWAPQVPVLNHKAVGGFVTHCGWNSILEAVCAGVPMVAWPLYA
EQRFNRVMIVDEIKIAISMNESETGFVSSTEVEKRVQEIIGECPVRERTMAMKN
AAELALTETDCNSDG*
>AT2G29750
MGKQEDAELVIIPFPFSGHILATIELAKRLISQDNPRIHTITILYWGLPFIPQADTI

AFLRSLVKNEPRIRLVTLPEVQDPPPMELFVEFAESYILEYVKKMVPIIREALST
LLSSRDESGSVRVAGLVLDFFCVPMIDVGNEFNLPSYIFLTCSAGFLGMMKYLP
ERHREIKSEFNRSFNEELNLIPGYVNSVPTKVLPSGLFMKETYEPWVELAERFP
EAKGILVNSYTALEPNGFKYFDRCPDNYPTIYPIGPILCSNDRPNLDSSERDRIIT
WLDDQPESSVVFLCFGSLKNLSATQINEIAQALEIVDCKFIWSFRTNPKEYASP
YEALPHGFMDRVMDQGIVCGWAPQVEILAHKAVGGFVSHCGWNSILESLGFG
VPIATWPMYAEQQLNAFTMVKELGLALEMRLDYVSEDGDIVKADEIAGTVRS
LMDGVDVPKSKVKEIAEAGKEAVDGGSSFLAVKRFIGDLIDGVSISK*
>AT3G46670
MEEKPAGRRVVLVAVPAQGHISPIMQLAKTLHLKGFSITIAQTKFNYFSPSDDF
TDFQFVTIPESLPESDFEDLGPIEFLHKLNKECQVSFKDCLGQLLLQQGNEIAC
VVYDEFMYFAEAAAKEFKLPNVIFSTTSATAFVCRSAFDKLYANSILTPLKEPK
GQQNELVPEFHPLRCKDFPVSHWASLESMMELYRNTVDKRTASSVIINTASCL
ESSSLSRLQQQLQIPVYPIGPLHLVASASTSLLEENKSCIEWLNKQKKNSVIFVS
LGSLALMEINEVIETALGLDSSKQQFLWVIRPGSVRGSEWIENLPKEFSKIISGR
GYIVKWAPQKEVLSHPAVGGFWSHCGWNSTLESIGEGVPMICKPFSSDQMVN
ARYLECVWKIGIQVEGDLDRGAVERAVRRLMVEEEGEGMRKRAISLKEQLRA
SVISGGSSHNSLEEFVHYMRTL* >AT3G21750
MKVELVFIPSPGVGHIRATTALAKLLVASDNRLSVTLIVIPSRVSDDASSSVYTN
SEDRLRYILLPARDQTTDLVSYIDSQKPQVRAVVSKVAGDVSTRSDSRLAGIVV
DMFCTSMIDIADEFNLSAYIFYTSNASYLGLQFHVQSLYDEKELDVSEFKDTE
MKFDVPTLTQPFPAKCLPSVMLNKKWFPYVLGRARSFRATKGILVNSVADME
PQALSFFSGGNGNTNIPPVYAVGPIMDLESSGDEEKRKEILHWLKEQPTKSVVF
LCFGSMGGFSEEQAREIAVALERSGHRFLWSLRRASPVGNKSNPPPGEFTNLEE
ILPKGFLDRTVEIGKIISWAPQVDVLNSPAIGAFVTHCGWNSILESLWFGVPMA
AWPIYAEQQFNAFHMVDELGLAAEVKKEYRRDFLVEEPEIVTADEIERGIKCA
MEQDSKMRKRVMEMKDKLHVALVDGGSSNCALKKFVQDVVDNVP*
>AT2G22930
MEPTFHAFMFPWFAFGHMIPFLHLANKLAEKGHQITFLLPKKAQKQLEHHNL
FPDSIVFHPLTIPHVNGLPAGAETTSDISISMDNLLSEALDLTRDQVEAAVRALR
PDLIFFDFAHWIPEIAKEHMIKSVSYMIVSATTIAYTFAPGGVLGVPPPGYPSSK
VLYRENDAHALATLSIFYKRLYHQITTGFKSCDIIALRTCNEIEGKFCDYISSQY
HKKVLLTGPMLPEQDTSKPLEEQLSHFLSRFPPRSVVFCALGSQIVLEKDQFQE
LCLGMELTGLPFLIAVKPPRGSSTVEEGLPEGFQERVKGRGVVWGGWVQQPLI
LDHPSIGCFVNHCGPGTIWECLMTDCQMVLLPFLGDQVLFTRLMTEEFKVSV
EVSREKTGWFSKESLSDAIKSVMDKDSDLGKLVRSNHAKLKETLGSHGLLTG
YVDKFVEELQEYLI*
>AT3G53160
MCSHDPLHFVVIPFMAQGHMIPLVDISRLLSQRQGVTVCIITTTQNVAKIKTSL
SFSSLFATINIVEVKFLSQQTGLPEGCESLDMLASMGDMVKFFDAANSLEEQV
EKAMEEMVQPRPSCIIGDMSLPFTSRLAKKFKIPKLIFHGFSCFSLMSIQVVRES
GILKMIESNDEYFDLPGLPDKVEFTKPQVSVLQPVEGNMKESTAKIIEADNDS
YGVIVNTFEELEVDYAREYRKARAGKVWCVGPVSLCNRLGLDKAKRGDKAS
IGQDQCLQWLDSQETGSVLYVCLGSLCNLPLAQLKELGLGLEASNKPFIWVIR

EWGKYGDLANWMQQSGFEERIKDRGLVIKGWAPQVFILSHASIGGFLTHCGW
NSTLEGITAGVPLLTWPLFAEQFLNEKLVVQILKAGLKIGVEKLMKYGKEEEIG
AMVSRECVRKAVDELMGDSEEAEERRRKVTELSDLANKALEKGGSSDSNITL
LIQDIMEQSQNQF*
>AT2G36760
MAFEKTRQFLPPLHFVLFPFMAQGHMIPMVDIARILAQRGVTITIVTTPHNAA
RFKDVLNRAIQSGLHIRVEHVKFPFQEAGLQEGQENVDFLDSMELMVHFFKA
VNMLENPVMKLMEEMKPKPSCLISDFCLPYTSKIAKRFNIPKIVFHGVSCFCLL
SMHILHRNHNILHALKSDKEYFLVPSFPDRVEFTKLQVTVKTNFSGDWKEIMD
EQVDADDTSYGVIVNTFQDLESAYVKNYTEARAGKVWSIGPVSLCNKVGED
KAERGNKAAIDQDECIKWLDSKDVESVLYVCLGSICNLPLAQLRELGLGLEAT
KRPFIWVIRGGGKYHELAEWILESGFEERTKERSLLIKGWSPQMLILSHPAVGG
FLTHCGWNSTLEGITSGVPLITWPLFGDQFCNQKLIVQVLKAGVSVGVEEVM
KWGEEESIGVLVDKEGVKKAVDEIMGESDEAKERRKRVRELGELAHKAVEEG
GSSHSNIIFLLQDIMQQVESKS*
>AT2G43840
MEKMRGHVLAVPFPSQGHITPIRQFCKRLHSKGFKTTHTLTTFIFNTIHLDPSSP
ISIATISDGYDQGGFSSAGSVPEYLQNFKTFGSKTVADIIRKHQSTDNPITCIVYD
SFMPWALDLAMDFGLAAAPFFTQSCAVNYINYLSYINNGSLTLPIKDLPLLELQ
DLPTFVTPTGSHLAYFEMVLQQFTNFDKADFVLVNSFHDLDLHEEELLSKVCP
VLTIGPTVPSMYLDQQIKSDNDYDLNLFDLKEAALCTDWLDKRPEGSVVYIA
FGSMAKLSSEQMEEIASAISNFSYLWVVRASEESKLPPGFLETVDKDKSLVLK
WSPQLQVLSNKAIGCFMTHCGWNSTMEGLSLGVPMVAMPQWTDQPMNAKY
IQDVWKVGVRVKAEKESGICKREEIEFSIKEVMEGEKSKEMKENAGKWRDLA
VKSLSEGGSTDININEFVSKIQIK*
>AT2G36780
MATEKTHQFHPSLHFVLFPFMAQGHMIPMIDIARLLAQRGVTITIVTTPHNAA
RFKNVLNRAIESGLAINILHVKFPYQEFGLPEGKENIDSLDSTELMVPFFKAVN
LLEDPVMKLMEEMKPRPSCLISDWCLPYTSIIAKNFNIPKIVFHGMGCFNLLC
MHVLRRNLEILENVKSDEEYFLVPSFPDRVEFTKLQLPVKANASGDWKEIMD
EMVKAEYTSYGVIVNTFQELEPPYVKDYKEAMDGKVWSIGPVSLCNKAGAD
KAERGSKAAIDQDECLQWLDSKEEGSVLYVCLGSICNLPLSQLKELGLGLEES
RRSFIWVIRGSEKYKELFEWMLESGFEERIKERGLLIKGWAPQVLILSHPSVGG
FLTHCGWNSTLEGITSGIPLITWPLFGDQFCNQKLVVQVLKAGVSAGVEEVM
KWGEEDKIGVLVDKEGVKKAVEELMGDSDDAKERRRRVKELGELAHKAVEK
GGSSHSNITLLLQDIMQLAQFKN*
>AT2G31790
MSEAKKGHVLFFPYPLQGHINPMIQLAKRLSKKGITSTLIIASKDHREPYTSDD
YSITVHTIHDGFFPHEHPHAKFVDLDRFHNSTSRSLTDFISSAKLSDNPPKALIY
DPFMPFALDIAKDLDLYVVAYFTQPWLASLVYYHINEGTYDVPVDRHENPTL
ASFPGFPLLSQDDLPSFACEKGSYPLLHEFVVRQFSNLLQADCILCNTFDQLEP
KVVKWMNDQWPVKNIGPVVPSKFLDNRLPEDKDYELENSKTEPDESVLKWL
GNRPAKSVVYVAFGTLVALSEKQMKEIAMAISQTGYHFLWSVRESERSKLPSG
FIEEAEEKDSGLVAKWVPQLEVLAHESIGCFVSHCGWNSTLEALCLGVPMVG

VPQWTDQPTNAKFIEDVWKIGVRVRTDGEGLSSKEEIARCIVEVMEGERGKEI
RKNVEKLKVLAREAISEGGSSDKKIDEFVALLT*
>AT3G46660
MQVLGMEEKPARRSVVLVPFPAQGHISPMMQLAKTLHLKGFSITVVQTKFNY
FSPSDDFTHDFQFVTIPESLPESDFKNLGPIQFLFKLNKECKVSFKDCLGQLVLQ
QSNEISCVIYDEFMYFAEAAAKECKLPNIIFSTTSATAFACRSVFDKLYANNVQ
APLKETKGQQEELVPEFYPLRYKDFPVSRFASLESIMEVYRNTVDKRTASSVII
NTASCLESSSLSFLQQQQLQIPVYPIGPLHMVASAPTSLLEENKSCIEWLNKQK
VNSVIYISMGSIALMEINEIMEVASGLAASNQHFLWVIRPGSIPGSEWIESMPEE
FSKMVLDRGYIVKWAPQKEVLSHPAVGGFWSHCGWNSTLESIGQGVPMICRP
FSGDQKVNARYLECVWKIGIQVEGELDRGVVERAVKRLMVDEEGEEMRKRA
FSLKEQLRASVKSGGSSHNSLEEFVHFIRTL*
>AT1G05680
MREGSHLIVLPFPGQGHITPMSQFCKRLASKGLKLTLVLVSDKPSPPYKTEHDS
ITVFPISNGFQEGEEPLQDLDDYMERVETSIKNTLPKLVEDMKLSGNPPRAIVY
DSTMPWLLDVAHSYGLSGAVFFTQPWLVTAIYYHVFKGSFSVPSTKYGHSTL
ASFPSFPMLTANDLPSFLCESSSYPNILRIVVDQLSNIDRVDIVLCNTFDKLEEK
LLKWVQSLWPVLNIGPTVPSMYLDKRLSEDKNYGFSLFNAKVAECMEWLNS
KEPNSVVYLSFGSLVILKEDQMLELAAGLKQSGRFFLWVVRETETHKLPRNY
VEEIGEKGLIVSWSPQLDVLAHKSIGCFLTHCGWNSTLEGLSLGVPMIGMPHW
TDQPTNAKFMQDVWKVGVRVKAEGDGFVRREEIMRSVEEVMEGEKGKEIR
KNAEKWKVLAQEAVSEGGSSDKSINEFVSMFC*
>AT2G43820
MEHKRGHVLAVPYPTQGHITPFRQFCKRLHFKGLKTTLALTTFVFNSINPDLS
GPISIATISDGYDHGGFETADSIDDYLKDFKTSGSKTIADIIQKHQTSDNPITCIV
YDAFLPWALDVAREFGLVATPFFTQPCAVNYVYYLSYINNGSLQLPIEELPFLE
LQDLPSFFSVSGSYPAYFEMVLQQFINFEKADFVLVNSFQELELHENELWSKA
CPVLTIGPTIPSIYLDQRIKSDTGYDLNLFESKDDSFCINWLDTRPQGSVVYVAF
GSMAQLTNVQMEELASAVSNFSFLWVVRSSEEEKLPSGFLETVNKEKSLVLK
WSPQLQVLSNKAIGCFLTHCGWNSTMEALTFGVPMVAMPQWTDQPMNAKYI
QDVWKAGVRVKTEKESGIAKREEIEFSIKEVMEGERSKEMKKNVKKWRDLA
VKSLNEGGSTDTNIDTFVSRVQSK*>AT3G46680
MEKMEEKKRIVLVPVPAQRHVTPMMQLGTALNMKGFSITVVEGQFNKVSSS
QNFPGFQFVTIPDTESLPESVLERLGPVEFLFEINKTSEASFKDCIRQSLLQQGN
DIACIIYDEYMYFCGAAAKEFNLPSVIFSTQSATNQVSRCVLRKLSAEKFLVD
MEDPEVQETLVENLHPLRYKDLPTSGVGPLDRLFELCREIVNKRTASAVIINTV
RCLESSSLKRLQHELGIPVYALGPLHITVSAASSLLEEDRSCVEWLNKQKPRSV
VYISLGSVVQMETKEVLEMARGLFNSNQPFLWVIRPGSIAGSEWIESLPEEVIK
MVSERGYIVKWAPQIEVLGHPAVGGFWSHCGWNSTLESIVEGVPMICRPFHG
EQKLNALCLESIWRIGFQVQGKVERGGVERAVKRLIVDEEGADMRERALVLK
ENLKASVRNGGSSYNALEEIVNLM*>AT3G21760
MKLELVFIPSPGDGHLRPLVEVAKLHVDRDDHLSITIIIIPQMHGFSSSNSSSYIA
SLSSDSEERLSYNVLSVPDKPDSDDTKPHFFDYIDNFKPQVKATVEKLTDPGPP
DSPSRLAGFVVDMFCMMMIDVANEFGVPSYMFYTSNATFLGLQVHVEYLYD

VKNYDVSDLKDSDTTELEVPCLTRPLPVKCFPSVLLTKEWLPVMFRQTRRFRE
TKGILVNTFAELEPQAMKFFSGVDSPLPTVYTVGPVMNLKINGPNSSDDKQSE
ILRWLDEQPRKSVVFLCFGSMGGFREGQAKEIAIALERSGHRFVWSLRRAQPK
GSIGPPEEFTNLEEILPEGFLERTAEIGKIVGWAPQSAILANPAIGGFVSHCGWN
STLESLWFGVPMATWPLYAEQQVNAFEMVEELGLAVEVRNSFRGDFMAADD
ELMTAEEIERGIRCLMEQDSDVRSRVKEMSEKSHVALMDGGSSHVALLKFIQD
VTKNIS*
>AT2G36770
MASEKSHKVHPPLHFILFPFMAQGHMIPMIDIARLLAQRGATVTIVTTRYNAG
RFENVLSRAMESGLPINIVHVNFPYQEFGLPEGKENIDSYDSMELMVPFFQAV
NMLEDPVMKLMEEMKPRPSCIISDLLLPYTSKIARKFSIPKIVFHGTGCFNLLC
MHVLRRNLEILKNLKSDKDYFLVPSFPDRVEFTKPQVPVETTASGDWKAFLDE
MVEAEYTSYGVIVNTFQELEPAYVKDYTKARAGKVWSIGPVSLCNKAGADK
AERGNQAAIDQDECLQWLDSKEDGSVLYVCLGSICNLPLSQLKELGLGLEKS
QRSFIWVIRGWEKYNELYEWMMESGFEERIKERGLLIKGWSPQVLILSHPSVG
GFLTHCGWNSTLEGITSGIPLITWPLFGDQFCNQKLVVQVLKAGVSAGVEEV
MKWGEEEKIGVLVDKEGVKKAVEELMGASDDAKERRRRVKELGESAHKAVE
EGGSSHSNITYLLQDIMQQVKSKN*
>AT5G38040
MEEKLSRRRRVVLVPVPAQGHITPMIQLAKALHSKGFSITVVQTKFNYLNPSN
DLSDFQFVTIPENLPVSDLKNLGPGRFLIKLANECYVSFKDLLGQLLVNEEEEI
ACVIYDEFMYFVEVAVKEFKLRNVILSTTSATAFVCRFVMCELYAKDGLAQLK
EGGEREVELVPELYPIRYKDLPSSVFASVESSVELFKNTCYKGTASSVIINTVRC
LEMSSLEWLQQELEIPVYSIGPLHMVVSAPPTSLLEENESCIEWLNKQKPSSVI
YISLGSFTLMETKEMLEMAYGFVSSNQHFLWVIRPGSICGSEISEEELLKKMVI
TDRGYIVKWAPQKQVLAHSAVGAFWSHCGWNSTLESLGEGVPLICRPFTTDQ
KGNARYLECVWKVGIQVEGELERGAIERAVKRLMVDEEGEEMKRRALSLKE
KLKASVLAQGSSHKSLDDFIKTL*
>AT1G64910
MGQTFHAFMFPWFAFGHMTPYLHLANKLAERGHRITFLIPKKAQKQLEHLNL
FPDSIVFHSLTIPHVDGLPAGAETFSDIPMPLWKFLPPAIDLTRDQVEAAVSALSP
DLILFDIASWVPEVAKEYRVKSMLYNIISATSIAHDFVPGGELGVPPPGYPSSKL
LYRKHDAHALLSFSVYYKRFSHRLITGLMNCDFISIRTCKEIEGKFCEYLERQY
HKKVFLTGPMLPEPNKGKPLEDRWSHWLNGFEQGSVVFCALGSQVTLEKDQ
FQELCLGIELTGLPFFVAVTPPKGAKTIQDALPEGFEERVKDRGVVLGEWVQQ
PLLLAHPSVGCFLSHCGFGSMWESIMSDCQIVLLPFLADQVLNTRLMTEELKV
SVEVQREETGWFSKESLSVAITSVMDQASEIGNLVRRNHSKLKEVLVSDGLLT
GYTDKFVDTLENLVSETKRE* >AT1G51210
MNRAMPQPRPETIRGSLKPHIMVFPYPAQGHLLPLLDLTHQLCLRGLTVSIIVT
PKNLPYLSPLLSAHPSAVSVVTLPFPHHPLIPSGVENVKDLGGYGNPLIMASLR
QLREPIVNWLSSHPNPPVALISDFFLGWTKDLGIPRFAFFSSGAFLASILHFVSD
KPHLFESTEPVCLSDLPRSPVFKTEHLPSLIPQSPLSQDLESVKDSTMNFSSYGC
IFNTCECLEEDYMEYVKQKVSENRVFGVGPLSSVGLSKEDSVSNVDAKALLS
WLDGCPDDSVLYICFGSQKVLTKEQCDDLALGLEKSMTRFVWVVKKDPIPDG

FEDRVAGRGMIVRGWAPQVAMLSHVAVGGFLIHCGWNSVLEAMASGTMILA
WPMEADQFVDARLVVEHMGVAVSVCEGGKTVPDPYEMGRIIADTMGESGGE
ARARAKEMG*
>AT1G05675
MREGSHVIVLPFPAQGHITPMSQFCKRLASKSLKITLVLVSDKPSPPYKTEHDTI
TVVPISNGFQEGQERSEDLDEYMERVESSIKNRLPKLIEDMKLSGNPPRALVY
DSTMPWLLDVAHSYGLSGAVFFTQPWLVSAIYYHVFKGSFSVPSTKYGHSTL
ASFPSLPILNANDLPSFLCESSSYPYILRTVIDQLSNIDRVDIVLCNTFDKLEEKL
LKWIKSVWPVLNIGPTVPSMYLDKRLAEDKNYGFSLFGAKIAECMEWLNSK
QPSSVVYVSFGSLVVLKKDQLIELAAGLKQSGHFFLWVVRETERRKLPENYIE
EIGEKGLTVSWSPQLEVLTHKSIGCFVTHCGWNSTLEGLSLGVPMIGMPHWA
DQPTNAKFMEDVWKVGVRVKADSDGFVRREEFVRRVEEVMEAEQGKEIRK
NAEKWKVLAQEAVSEGGSSDKNINEFVSMFC*
>AT5G03490
MTEVLLLPGTKSENSKPPHIVVFPFPAQGHLLPLLDLTHQLCLRGFNVSVIVTP
GNLTYLSPLLSAHPSSVTSVVFPFPPHPSLSPGVENVKDVGNSGNLPIMASLRQ
LREPIINWFQSHPNPPIALISDFFLGWTHDLCNQIGIPRFAFFSISFFLVSVLQFCF
ENIDLIKSTDPIHLLDLPRAPIFKEEHLPSIVRRSLQTPSPDLESIKDFSMNLLSY
GSVFNSSEILEDDYLQYVKQRMGHDRVYVIGPLCSIGSGLKSNSGSVDPSLLS
WLDGSPNGSVLYVCFGSQKALTKDQCDALALGLEKSMTRFVWVVKKDPIPD
GFEDRVSGRGLVVRGWVSQLAVLRHVAVGGFLSHCGWNSVLEGITSGAVILG
WPMEADQFVNARLLVEHLGVAVRVCEGGETVPDSDELGRVIAETMGEGGREV
AARAEEIRRKTEAAVTEANGSSVENVQRLVKEFEKV*
>AT3G29630
MGSKFHAFLYPWFGFGHMIPYLHLANKLAEKGHRVTFLAPKKAQKQLEPLNL
FPNSIHFENVTLPHVDGLPVGAETTADLPNSSKRVLADAMDLLREQIEVKIRSL
KPDLIFFDFVDWIPQMAKELGIKSVSYQIISAAFIAMFFAPRAELGSPPPGFPSS
KVALRGHDANIYSLFANTRKFLFDRVTTGLKNCDVIAIRTCAEIEGNLCDFIER
QCQRKVLLTGPMFLDPQGKSGKPLEDRWNNWLNGFEPSSVVYCAFGTHFFFE
IDQFQELCLGMELTGLPFLVAVMPPRGSSTIQEALPEGFEERIKGRGIVWGGWV
EQPLILSHPSIGCFVNHCGFGSMWESLVSDCQIVFIPQLVDQVLTTRLLTEELEV
SVKVKRDEITGWFSKESLRDTVKSVMDKNSEIGNLVRRNHKKLKETLVSPGL
LSSYADKFVDELENHIHSKN* >AT1G22400
MGSQIIHNSQKPHVVCVPYPAQGHINPMMRVAKLLHARGFYVTFVNTVYNH
NRFLRSRGSNALDGLPSFRFESIADGLPETDMDATQDITALCESTMKNCLAPFR
ELLQRINAGDNVPPVSCIVSDGCMSFTLDVAEELGVPEVLFWTTSGCAFLAYL
HFYLFIEKGLCPLKDESYLTKEYLEDTVIDFIPTMKNVKLKDIPSFIRTTNPDDV
MISFALRETERAKRASAIILNTFDDLEHDVVHAMQSILPPVYSVGPLHLLANRE
IEEGSEIGMMSSNLWKEEMECLDWLDTKTQNSVIYINFGSITVLSVKQLVEFA
WGLAGSGKEFLWVIRPDLVAGEEAMVPPDFLMETKDRSMLASWCPQEKVLS
HPAIGGFLTHCGWNSILESLSCGVPMVCWPFFADQQMNCKFCCDEWDVGIEI
GGDVKREEVEAVVRELMDGEKGKKMREKAVEWQRLAEKATEHKLGSSVMN
FETVVSKFLLGQKSQD*
>AT1G78270

MEQHGGSSSQKPHAMCIPYPAQGHINPMLKLAKLLHARGFHVTFVNTDYNH
RRILQSRGPHALNGLPSFRFETIPDGLPWTDVDAKQDMLKLIDSTINNCLAPFK
DLILRLNSGSDIPPVSCIISDASMSFTIDAAEELKIPVVLLWTNSATALILYLHYQ
KLIEKEIIPLKDSSDLKKHLETEIDWIPSMKKIKLKDFPDFVTTTNPQDPMISFIL
HVTGRIKRASAIFINTFEKLEHNVLLSLRSLLPQIYSVGPFQILENREIDKNSEIR
KLGLNLWEEETESLDWLDTKAEKAVIYVNFGSLTVLTSEQILEFAWGLARSGK
EFLWVVRSGMVDGDDSILPAEFLSETKNRGMLIKGWCSQEKVLSHPAIGGFLT
HCGWNSTLESLYAGVPMICWPFFADQLTNRKFCCEDWGIGMEIGEEVKRERV
ETVVKELMDGEKGKRLREKVVEWRRLAEEASAPPLGSSYVNFETVVNKVLT
CHTIRST*
>AT5G59590
MEEKQVKETRIVLVPVPAQGHVTPMMQLGKALHSKGFSITVVLTQSNRVSSS
KDFSDFHFLTIPGSLTESDLQNLGPQKFVLKLNQICEASFKQCIGQLLHEQCNN
DIACVVYDEYMYFSHAAVKEFQLPSVVFSTTSATAFVCRSVLSRVNAESFLID
MKDPETQDKVFPGLHPLRYKDLPTSVFGPIESTLKVYSETVNTRTASAVIINSA
SCLESSSLARLQQQLQVPVYPIGPLHITASAPSSLLEEDRSCVEWLNKQKSNSV
IYISLGSLALMDTKDMLEMAWGLSNSNQPFLWVVRPGSIPGSEWTESLPEEFN
RLVSERGYIVKWAPQMEVLRHPAVGGFWSHCGWNSTVESIGEGVPMICRPFT
GDQKVNARYLERVWRIGVQLEGDLDKETVERAVEWLLVDEEGAEMRKRAID
LKEKIETSVRSGGSSCSSLDDFVNSM*
>AT5G59580
MEELGVKRRIVLVPVPAQGHVTPIMQLGKALYSKGFSITVVLTQYNRVSSSKD
FSDFHFLTIPGSLTESDLKNLGPFKFLFKLNQICEASFKQCIGQLLQEQGNDIAC
VVYDEYMYFSQAAVKEFQLPSVLFSTTSATAFVCRSVLSRVNAESFLLDMKDP
KVSDKEFPGLHPLRYKDLPTSAFGPLESILKVYSETVNIRTASAVIINSTSCLESS
SLAWLQKQLQVPVYPIGPLHIAASAPSSLLEEDRSCLEWLNKQKIGSVIYISLG
SLALMETKDMLEMAWGLRNSNQPFLWVIRPGSIPGSEWTESLPEEFSRLVSER
GYIVKWAPQIEVLRHPAVGGFWSHCGWNSTLESIGEGVPMICRPFTGDQKVN
ARYLERVWRIGVQLEGELDKGTVERAVERLIMDEEGAEMRKRVINLKEKLQA
SVKSRGSSFSSLDNFVNSLKMMNFM*
>AT1G73880
MKVNEENNKPTKTHVLIFPFPAQGHMIPLLDFTHRLALRGGAALKITVLVTPK
NLPFLSPLLSAVVNIEPLILPFPSHPSIPSGVENVQDLPPSGFPLMIHALGNLHAP
LISWITSHPSPPVAIVSDFFLGWTKNLGIPRFDFSPSAAITCCILNTLWIEMPTKI
NEDDDNEILHFPKIPNCPKYRFDQISSLYRSYVHGDPAWEFIRDSFRDNVASWG
LVVNSFTAMEGVYLEHLKREMGHDRVWAVGPIIPLSGDNRGGPTSVSVDHVM
SWLDAREDNHVVYVCFGSQVVLTKEQTLALASGLEKSGVHFIWAVKEPVEK
DSTRGNILDGFDDRVAGRGLVIRGWAPQVAVLRHRAVGAFLTHCGWNSVVEA
VVAGVLMLTWPMRADQYTDASLVVDELKVGVRACEGPDTVPDPDELARVFA
DSVTGNQTERIKAVELRKAALDAIQERGSSVNDLDGFIQHVVSLGLNK*
>AT3G46700
MEKRVEKRRIVLVPLPLLGHFTPMMQLGQALILKGFSIIVPQGEFNRVNSSQKF
PGFQFITIPDSELEANGPVGSLTQLNKIMEASFKDCIRQLLKQQGNDIACIIYDE
FMYFCGAVAEELKLPNFIFSTQTATHKVCCNVLSKLNAKKYLIDMEEHDVQN

KVVENMHPLRYKDLPTATFGELEPFLELCRDVVNKRTASAVIINTVTCLESSSL
TRLQQELQIPVYPLGPLHITDSSTGFTVLQEDRSCVEWLNKQKPRSVIYISLGS
MVLMETKEMLEMAWGMLNSNQPFLWVIRPGSVSGSEGIESLPEEVSKMVLE
KGYIVKWAPQIEVLGHPSVGGFWSHCGWNSTLESIVEGVPMICRPYQGEQML
NAIYLESVWRIGIQVGGELERGAVERAVKRLIVDKEGASMRERTLVLKEKLKA
SIRGGGSSCNALDELVKHLKTE* >AT3G11340
METRETKPVIFLFPFPLQGHLNPMFQLANIFFNRGFSITVIHTEFNSPNSSNFPHF
TFVSIPDSLSEPESYPDVIEILHDLNSKCVAPFGDCLKKLISEEPTAACVIVDAL
WYFTHDLTEKFNFPRIVLRTVNLSAFVAFSKFHVLREKGYLSLQETKADSPVP
ELPYLRMKDLPWFQTEDPRSGDKLQIGVMKSLKSSSGIIFNAIEDLETDQLDE
ARIEFPVPLFCIGPFHRYVSASSSSLLAHDMTCLSWLDKQATNSVIYASLGSIAS
IDESEFLEIAWGLRNSNQPFLWVVRPGLIHGKEWIEILPKGFIENLEGRGKIVK
WAPQPEVLAHRATGGFLTHCGWNSTLEGICEAIPMICRPSFGDQRVNARYIND
VWKIGLHLENKVERLVIENAVRTLMTSSEGEEIRKRIMPMKETVEQCLKLGGS
SFRNLENLIAYILSF*
>AT3G55710
MEERKVKRIIMFPLPFTGHFNPMIELAGIFHNRGFSVTILHTSFNFPDPSRHPQF
TFRTITHKNEGEEDPLSQSETSSGKDLVVLISLLKQYYTEPSLAEEVGEGGTVC
CLVSDALWGRNTEIVAKEIGVCTMVMRTSGAATFCAYTAFPLLIDKGYLPIQGS
RLDELVTELPPLKVKDLPVIKTKEPEGLNRILNDMVEGAKLSSGVVWNTFEDL
ERHSLMDCRSKLQVPLFPIGPFHKHRTDLPPKPKNKDKDDDEILTDWLNKQAP
QSVVYVSFGSLAAIEENEFFEIAWGLRNSELPFLWVVRPGMVRGTEWLESLPC
GFLENIGHQGKIVKWVNQLETLAHPAVGAFWTHCGWNSTIESICEGVPMICTP
CFSDQHVNARYIVDVWRVGMMLERCKMERTEIEKVVTSVMMENGAGLTEM
CLELKEKANVCLSEDGSSSKYLDKLVSHVLSFDSSAFAS*
>AT3G46650
MEKKMEAKRRIVLVPIPAQGHVTPLMQLGKVLNSKGFSITVVEGHFNQVSSSS
QHFPGFQFVTIKESLPESEFEKLGGIESMITLNKTSEASFKDCISQLLLQQGNDI
ACIIYDEYMYFCGAAAKEFSIPSVIFSTQSAANYVSHPDMQDKVVENLYPLRY
KDLPTSGMGPLDRFFELCREVANKRTASAVIINTVSCLESSSLSWLEQKVGISV
YPLGPLHMTDSSPSSLLEEDRSCIEWLNKQKPKSVIYISIGTLGQMETKEVLEM
SWGLCNSNQPFLWVIRAGSILGTNGIESLPEDVNKMVSERGYIVKRAPQIEVL
GHPAVGGFWSHCGWNSILESIGEGVPMICKPFHGEQKLNAMYLECVWKIGIQ
VEGDLERGAVERAVKRLTVFEEGEEMRKRAVTLKEELRASVRGGGSLHNSLK
EFEHFMMTL*
>AT2G31750
MGEKAKANVLVFSFPIQGHINPLLQFSKRLLSKNVNVTFLTTSSTHNSILRRAIT
GGATALPLSFVPIDDGFEEDHPSTDTSPDYFAKFQENVSRSLSELISSMDPKPNA
VVYDSCLPYVLDVCRKHPGVAAASFFTQSSTVNATYIHFLRGEFKEFQNDVVL
PAMPPLKGNDLPVFLYDNNLCRPLFELISSQFVNVDDIDFFLVNSFDELEVEVL
QWMKNQWPVKNIGPMIPSMYLDKRLAGDKDYGINLFNAQVNECLDWLDSK
PPGSVIYVSFGSLAVLKDDQMIEVAAGLKQTGHNFLWVVRETETKKLPSNYIE
DICDKGLIVNWSPQLQVLAHKSIGCFMTHCGWNSTLEALSLGVALIGMPAYSD
QPTNAKFIEDVWKVGVRVKADQNGFVPKEEIVRCVGEVMEDMSEKGKEIRK

NARRLMEFAREALSDGGNSDKNIDEFVAKIVR*
>AT5G38010
MEEKQERRRRIVLIPAPAQGHISPMMQLARALHLKGFSITVAQTKFNYLKPSK
DLADFQFITIPESLPASDLKNLGPVWFLLKLNKECEFSFKECLGQLLLQKQLIPE
EEIACVIYDEFMYFAEAAAKEFNLPKVIFSTENATAFACRSAMCKLYAKDGLA
PLKEGCGREEELVPKLHPLRYKDLPTSAFAPVEASVEVFKSSCDKGTASAMIIN
TVRCLEISSLEWLQQELKIPIYPIGPLHMVSSAPPTSLLDENESCIDWLNKQKPS
SVIYISLGSFTLLETKEVLEMASGLVSSNQHFLWVIRPGSILGSELTNEELLSMM
EIPDRGYIVKWAPQKQVLAHSAVGAFWSHCGWNSTLESMGEGVPMICRPFTT
DQKVNARYVECVWRVGVQVEGELKRGVVERAVKRLLVDEEGEEMKLRALS
LKEKLKVSVLPGGSSHSSLDDLIKTL* >AT2G36750
MASEFRPPLHFVLFPFMAQGHMIPMVDIARLLAQRGVTITIVTTPQNAGRFKN
VLSRAIQSGLPINLVQVKFPSQESGSPEGQENLDLLDSLGASLTFFKAFSLLEEP
VEKLLKEIQPRPNCIIADMCLPYTNRIAKNLGIPKIIFHGMCCFNLLCTHIMHQ
NHEFLETIESDKEYFPIPNFPDRVEFTKSQLPMVLVAGDWKDFLDGMTEGDNT
SYGVIVNTFEELEPAYVRDYKKVKAGKIWSIGPVSLCNKLGEDQAERGNKADI
DQDECIKWLDSKEEGSVLYVCLGSICNLPLSQLKELGLGLEESQRPFIWVIRG
WEKYNELLEWISESGYKERIKERGLLITGWSPQMLILTHPAVGGFLTHCGWNS
TLEGITSGVPLLTWPLFGDQFCNEKLAVQILKAGVRAGVEESMRWGEEEKIGV
LVDKEGVKKAVEELMGDSNDAKERRKRVKELGELAHKAVEEGGSSHSNITFL
LQDIMQLEQPKK*
>AT5G54060
MGVFGSNESSSMSIVMYPWLAFGHMTPFLHLSNKLAEKGHKIVFLLPKKALN
QLEPLNLYPNLITFHTISIPQVKGLPPGAETNSDVPFFLTHLLAVAMDQTRPEVE
TIFRTIKPDLVFYDSAHWIPEIAKPIGAKTVCFNIVSAASIALSLVPSAEREVIDG
KEMSGEELAKTPLGYPSSKVVLRPHEAKSLSFVWRKHEAIGSFFDGKVTAMR
NCDAIAIRTCRETEGKFCDYISRQYSKPVYLTGPVLPGSQPNQPSLDPQWAEW
LAKFNHGSVVFCAFGSQPVVNKIDQFQELCLGLESTGFPFLVAIKPPSGVSTVE
EALPEGFKERVQGRGVVFGGWIQQPLVLNHPSVGCFVSHCGFGSMWESLMS
DCQIVLVPQHGEQILNARLMTEEMEVAVEVEREKKGWFSRQSLENAVKSVME
EGSEIGEKVRKNHDKWRCVLTDSGFSDGYIDKFEQNLIELVKS*
>AT2G36800
MVSETTKSSPLHFVLFPFMAQGHMIPMVDIARLLAQRGVIITIVTTPHNAARF
KNVLNRAIESGLPINLVQVKFPYLEAGLQEGQENIDSLDTMERMIPFFKAVNFL
EEPVQKLIEEMNPRPSCLISDFCLPYTSKIAKKFNIPKILFHGMGCFCLLCMHV
LRKNREILDNLKSDKELFTVPDFPDRVEFTRTQVPVETYVPAGDWKDIFDGMV
EANETSYGVIVNSFQELEPAYAKDYKEVRSGKAWTIGPVSLCNKVGADKAER
GNKSDIDQDECLKWLDSKKHGSVLYVCLGSICNLPLSQLKELGLGLEESQRPF
IWVIRGWEKYKELVEWFSESGFEDRIQDRGLLIKGWSPQMLILSHPSVGGFLT
HCGWNSTLEGITAGLPLLTWPLFADQFCNEKLVVEVLKAGVRSGVEQPMKW
GEEEKIGVLVDKEGVKKAVEELMGESDDAKERRRRAKELGDSAHKAVEEGG
SSHSNISFLLQDIMELAEPNN*
>AT1G64920
MGQKIHAFMFPWFAFGHMTPYLHLGNKLAEKGHRVTFLLPKKAQKQLEHQN

LFPHGIVFHPLVIPHVDGLPAGAETASDIPISLVKFLSIAMDLTRDQIEAAIGALR
PDLILFDLAHWVPEMAKALKVKSMLYNVMSATSIAHDLVPGGELGVAPPGYP
SSKALYREHDAHALLTFSGFYKRFYHRFTTGLMNCDFISIRTCEEIEGKFCDYIE
SQYKKKVLLTGPMLPEPDKSKPLEDQWSHWLSGFGQGSVVFCALGSQTILEK
NQFQELCLGIELTGLPFLVAVKPPKGANTIHEALPEGFEERVKGRGIVWGEWV
QQPSWQPLILAHPSVGCFVSHCGFGSMWESLMSDCQIVFIPVLNDQVLTTRV
MTEELEVSVEVQREETGWFSKENLSGAIMSLMDQDSEIGNQVRRNHSKLKET
LASPGLLTGYTDKFVDTLENLVNEQGYIS*
>AT3G53150
MESKIVSKAKRLHFVLIPLMAQGHLIPMVDISKILARQGNIVTIVTTPQNASRF
AKTVDRARLESGLEINVVKFPIPYKEFGLPKDCETLDTLPSKDLLRRFYDAVD
KLQEPMERFLEQQDIPPSCIISDKCLFWTSRTAKRFKIPRIVFHGMCCFSLLSSH
NIHLHSPHLSVSSAVEPFPIPGMPHRIEIARAQLPGAFEKLANMDDVREKMRES
ESEAFGVIVNSFQELEPGYAEAYAEAINKKVWFVGPVSLCNDRMADLFDRGS
NGNIAISETECLQFLDSMRPRSVLYVSLGSLCRLIPNQLIELGLGLEESGKPFIW
VIKTEEKHMIELDEWLKRENFEERVRGRGIVIKGWSPQAMILSHGSTGGFLTH
CGWNSTIEAICFGVPMITWPLFAEQFLNEKLIVEVLNIGVRVGVEIPVRWGDEE
RLGVLVKKPSVVKAIKLLMDQDCQRVDENDDDNEFVRRRRRIQELAVMAKK
AVEEKGSSSINVSILIQDVLEQLSLV*
>AT4G09500
MEPKFHAFMFPWFAFGHMIPFLHLANKLAEKGHRVTFLLPKKAQKQLEHHN
LFPDSIVFHPLTVPPVNGLPAGAETTSDIPISLDNLLSKALDLTRDQVEAAVRAL
RPDLIFFDFAQWIPDMAKEHMIKSVSYIIVSATTIAHTHVPGGKLGVRPPGYPS
SKVMFRENDVHALATLSIFYKRLYHQITTGLKSCDVIALRTCKEVEDTSKPLEE
RWNHFLSGFAPKSVVFCSPGSQVILEKDQFQELCLGMELTGLPFLLAVKPPRGS
STVQEGLPEGFEERVKDRGVVWGGWVQQPLILAHPSIGCFVNHCGPGTIWES
LVSDCQMVLIPFLSDQVLFTRLMTEEFEVSVEVPREKTGWFSKESLSNAIKSV
MDKDSDIGKLVRSNHTKLKEILVSPGLLTGYVDHFVEGLQENLI*
>AT5G17050
MTKPSDPTRDSHVAVLAFPFGTHAAPLLTVTRRLASASPSTVFSFFNTAQSNSS
LFSSGDEADRPANIRVYDIADGVPEGYVFSGRPQEAIELFLQAAPENFRREIAK
AETEVGTEVKCLMTDAFFWFAADMATEINASWIAFWTAGANSLSAHLYTDLI
RETIGVKEVGERMEETIGVISGMEKIRVKDTPEGVVFGNLDSVFSKMLHQMG
LALPRATAVFINSFEDLDPTLTNNLRSRFKRYLNIGPLGLLSSTLQQLVQDPHGC
LAWMEKRSSGSVAYISFGTVMTPPPGELAAIAEGLESSKVPFVWSLKEKSLVQ
LPKGFLDRTREQGIVVPWAPQVELLKHEATGVFVTHCGWNSVLESVSGGVPM
ICRPFFGDQRLNGRAVEVVWEIGMTIINGVFTKDGFEKCLDKVLVQDDGKKM
KCNAKKLKELAYEAVSSKGRSSENFRGLLDAVVNII*
>AT5G14860
MAVSSSHHAVLFPYMSKGHTIPLLQFARLLLRHRRIVSVDDEEPTISVTVFTTP
KNQPFVSNFLSDVASSIKVISLPFPENIAGIPPGVESTDMLPSISLYVPFTRATKSL
QPFFEAELKNLEKVSFMVSDGFLWWTSESAAKFEIPRLAFYGMNSYASAMCS
AISVHELFTKPESVKSDTEPVTVPDFPWICVKKCEFDPVLTEPDQSDPAFELLID
HLMSTKKSRGVIVNSFYELESTFVDYRLRDNDEPKPWCVGPLCLVNPPKPESD

KPDWIHWLDRKLEERCPVMYVAFGTQAEISNEQLKEIALGLEDSKVNFLWVT
RKDLEEVTGGLGFEKRVKEHGMIVRDWVDQWEILSHKSVKGFLSHCGWNSA
QESICAGVPLLAWPMMAEQPLNAKLVVEELKIGVRIETEDVSVKGFVTREELS
RKVKQLMEGEMGKTTMKNVKEYAKMAKKAMAQGTGSSWKSLDSLLEELC
KSREPDGVNKLSSSDA*
>AT2G36970
MERAKSRKPHIMMIPYPLQGHVIPFVHLAIKLASHGFTITFVNTDSIHHHISTAH
QDDAGDIFSAARSSGQHDIRYTTVSDGFPLDFDRSLNHDQFFEGILHVFSAHV
DDLIAKLSRRDDPPVTCLIADTFYVWSSMICDKHNLVNVSFWTEPALVLNLYY
HMDLLISNGHFKSLDNRKDVIDYVPGVKAIEPKDLMSYLQVSDKDVDTNTV
VYRILFKAFKDVKRADFVVCNTVQELEPDSLSALQAKQPVYAIGPVFSTDSVV
PTSLWAESDCTEWLKGRPTGSVLYVSFGSYAHVGKKEIVEIAHGLLLSGISFIW
VLRPDIVGSNVPDFLPAGFVDQAQDRGLVVQWCCQMEVISNPAVGGFFTHCG
WNSILESVWCGLPLLCYPLLTDQFTNRKLVVDDWCIGINLCEKKTITRDQVSA
NVKRLMNGETSSELRNNVEKVKRHLKDAVTTVGSSETNFNLFVSEVRNRIET
KLCNVNGLEISPSN*
>AT5G12890
MAEAKPRNLRIVMFPFMGQGHIIPFVALALRLEKIMIMNRANKTTISMINTPSN
IPKIRSNLPPESSISLIELPFNSSDHGLPHDGENFDSLPYSLVISLLEASRSLREPFR
DFMTKILKEEGQSSVIVIGDFFLGWIGKVCKEVGVYSVIFSASGAFGLGCYRSI
WLNLPHKETKQDQFLLDDFPEAGEIEKTQLNSFMLEADGTDDWSVFMKKIIP
GWSDFDGFLFNTVAEIDQMGLSYFRRITGVPVWPVGPVLKSPDKKVGSRSTE
EAVKSWLDSKPDHSVVYVCFGSMNSILQTHMLELAMALESSEKNFIWVVRPP
IGVEVKSEFDVKGYLPEGFEERITRSERGLLVKKWAPQVDILSHKATCVFLSHC
GWNSILESLSHGVPLLGWPMAAEQFFNSILMEKHIGVSVEVARGKRCEIKCDD
IVSKIKLVMEETEVGKEIRKKAREVKELVRRAMVDGVKGSSVIGLEEFLDQA
MVKKVEN*
>AT1G22360
MGSHVAQKQHVVCVPYPAQGHINPMMKVAKLLYAKGFHITFVNTVYNHNRL
LRSRGPNAVDGLPSFRFESIPDGLPETDVDVTQDIPTLCESTMKHCLAPFKELL
RQINARDDVPPVSCIVSDGCMSFTLDAAEELGVPEVLFWTTSACGFLAYLYYY
RFIEKGLSPIKDESYLTKEHLDTKIDWIPSMKNLRLKDIPSFIRTTNPDDIMLNFI
IREADRAKRASAIILNTFDDLEHDVIQSMKSIVPPVYSIGPLHLLEKQESGEYSE
IGRTGSNLWREETECLDWLNTKARNSVVYVNFGSITVLSAKQLVEFAWGLAA
TGKEFLWVIRPDLVAGDEAMVPPEFLTATADRRMLASWCPQEKVLSHPAIGGF
LTHCGWNSTLESLCGGVPMVCWPFFAEQQTNCKFSRDEWEVGIEIGGDVKRE
EVEAVVRELMDEEKGKNMREKAEEWRRLANEATEHKHGSSKLNFEMLVNK
VLLGE*
>AT1G50580
MGSKFHAFMYPWFGFGHMIPYLHLANKLAEKGHRVTFFLPKKAHKQLQPLN
LFPDSIVFEPLTLPPVDGLPFGAETASDLPNSTKKPIFVAMDLLRDQIEAKVRAL
KPDLIFFDFVHWVPEMAEEFGIKSVNYQIISAACVAMVLAPRAELGFPPPDYPL
SKVALRGHEANVCSLFANSHELFGLITKGLKNCDVVSIRTCVELEGKLCGFIEK
ECQKKLLLTGPMLPEPQNKSGKFLEDRWNHWLNGFEPGSVVFCAFGTQFFFE

KDQFQEFCLGMELMGLPFLISVMPPKGSPTVQEALPKGFEERVKKHGIVWEG
WLEQPLILSHPSVGCFVNHCGFGSMWESLVSDCQIVFIPQLADQVLITRLLTEE
LEVSVKVQREDSGWFSKEDLRDTVKSVMDIDSEIGNLVKRNHKKLKETLVSP
GLLSGYADKFVEALEIEVNNTKFS*
>AT5G05870
MEKRNERQVILFPLPLQGCINPMLQLAKILYSRGFSITIIHTRFNAPKSSDHPLFT
FLQIRDGLSESQTQSRDLLLQLTLLNNNCQIPFRECLAKLIKPSSDSGTEDRKIS
CVIDDSGWVFTQSVAESFNLPRFVLCAYKFSFFLGHFLVPQIRREGFLPVPDSE
ADDLVPEFPPLRKKDLSRIMGTSAQSKPLDAYLLKILDATKPASGIIVMSCKEL
DHDSLAESNKVFSIPIFPIGPFHIHDVPASSSSLLEPDQSCIPWLDMRETRSVVY
VSLGSIASLNESDFLEIACGLRNTNQSFLWVVRPGSVHGRDWIESLPSGFMESL
DGKGKIVRWAPQLDVLAHRATGGFLTHNGWNSTLESICEGVPMICLPCKWDQ
FVNARFISEVWRVGIHLEGRIERREIERAVIRLMVESKGEEIRGRIKVLRDEVRR
SVKQGGSSYRSLDELVDRISIIIEPLVPT*
>AT1G22380
MGSRFVSNEQKPHVVCVPYPAQGHINPMMKVAKLLHVKGFHVTFVNTVYNH
NRLLRSRGANALDGLPSFQFESIPDGLPETGVDATQDIPALSESTTKNCLVPFK
KLLQRIVTREDVPPVSCIVSDGSMSFTLDVAEELGVPEIHFWTTSACGFMAYL
HFYLFIEKGLCPVKDASCLTKEYLDTVIDWIPSMNNVKLKDIPSFIRTTNPNDI
MLNFVVREACRTKRASAIILNTFDDLEHDIIQSMQSILPPVYPIGPLHLLVNREI
EEDSEIGRMGSNLWKEETECLGWLNTKSRNSVVYVNFGSITIMTTAQLLEFAW
GLAATGKEFLWVMRPDSVAGEEAVIPKEFLAETADRRMLTSWCPQEKVLSHPA
VGGFLTHCGWNSTLESLSCGVPMVCWPFFAEQQTNCKFSCDEWEVGIEIGGD
VKRGEVEAVVRELMDGEKGKKMREKAVEWRRLAEKATKLPCGSSVINFETIV
NKVLLGKIPNT*
>AT3G22250
MKVTQKPKIIFIPYPAQGHVTPMLHLASAFLSRGFSPVVMTPESIHRRISATNED
LGITFLALSDGQDRPDAPPSDFFSIENSMENIMPPQLERLLLEEDLDVACVVVD
LLASWAIGVADRCGVPVAGFWPVMFAAYRLIQAIPELVRTGLVSQKGCPRQLE
KTIVQPEQPLLSAEDLPWLIGTPKAQKKRFKFWQRTLERTKSLRWILTSSFKDE
YEDVDNHKASYKKSNDLNKENNGQNPQILHLGPLHNQEATNNITITKTSFWE
EDMSCLGWLQEQNPNSVIYISFGSWVSPIGESNIQTLALALEASGRPFLWALNR
VWQEGLPPGFVHRVTITKNQGRIVSWAPQLEVLRNDSVGCYVTHCGWNSTM
EAVASSRRLLCYPVAGDQFVNCKYIVDVWKIGVRLSGFGEKEVEDGLRKVME
DQDMGERLRKLRDRAMGNEARLSSEMNFTFLKNELN*
>AT1G05560
MAPPHFLLVTFPAQGHVNPSLRFARRLIKRTGARVTFVTCVSVFHNSMIANHN
KVENLSFLTFSDGFDDGGISTYEDRQKRSVNLKVNGDKALSDFIEATKNGDSP
VTCLIYTILLNWAPKVARRFQLPSALLWIQPALVFNIYYTHFMGNKSVFELPNL
SSLEIRDLPSFLTPSNTNKGAYDAFQEMMEFLIKETKPKILINTFDSLEPEALTAF
PNIDMVAVGPLLPTEIFSGSTNKSVKDQSSSYTLWLDSKTESSVIYVSFGTMVE
LSKKQIEELARALIEGKRPFLWVITDKSNRETKTEGEEETEIEKIAGFRHELEEV
GMIVSWCSQIEVLSHRAVGCFVTHCGWSSTLESLVLGVPVVAFPMWSDQPTN
AKLLEESWKTGVRVRENKDGLVERGEIRRCLEAVMEEKSVELRENAKKWKR

LAMEAGREGGSSDKNMEAFVEDICGESLIQNLCEAEEVKVK*
>AT1G05530
MAQPHFLLVTFPAQGHVNPSLRFARRLIKTTGARVTFATCLSVIHRSMIPNHNN
VENLSFLTFSDGFDDGVISNTDDVQNRLVHFERNGDKALSDFIEANQNGDSPV
SCLIYTILPNWVPKVARRFHLPSVHLWIQPAFAFDIYYNYSTGNNSVFEFPNLPS
LEIRDLPSFLSPSNTNKAAQAVYQELMDFLKEESNPKILVNTFDSLEPEFLTAIP
NIEMVAVGPLLPAEIFTGSESGKDLSRDHQSSSYTLWLDSKTESSVIYVSFGTM
VELSKKQIEELARALIEGGRPFLWVITDKLNREAKIEGEEETEIEKIAGFRHELE
EVGMIVSWCSQIEVLRHRAIGCFLTHCGWSSSLESLVLGVPVVAFPMWSDQPA
NAKLLEEIWKTGVRVRENSEGLVERGEIMRCLEAVMEAKSVELRENAEKWK
RLATEAGREGGSSDKNVEAFVKSLF*
>AT4G15480
MGSISEMVFETCPSPNPIHVMLVSFQGQGHVNPLLRLGKLIASKGLLVTFVTTE
LWGKKMRQANKIVDGELKPVGSGSIRFEFFDEEWAEDDDRRADFSLYIAHLES
VGIREVSKLVRRYEEANEPVSCLINNPFIPWVCHVAEEFNIPCAVLWVQSCACF
SAYYHYQDGSVSFPTETEPELDVKLPCVPVLKNDEIPSFLHPSSRFTGFRQAIL
GQFKNLSKSFCVLIDSFDSLEQEVIDYMSSLCPVKTVGPLFKVARTVTSDVSGD
ICKSTDKCLEWLDSRPKSSVVYISFGTVAYLKQEQIEEIAHGVLKSGLSFLWVI
RPPPHDLKVETHVLPQELKESSAKGKGMIVDWCPQEQVLSHPSVACFVTHCG
WNSTMESLSSGVPVVCCPQWGDQVTDAVYLIDVFKTGVRLGRGATEERVVP
REEVAEKLLEATVGEKAEELRKNALKWKAEAEAAVAPGGSSDKNFREFVEKL
GAGVTKTKDNGY*
>AT5G17030
MAKPSQPTRDSHVAVLVFPFGTHAAPLLAVTCRLATAAPSTVFSFFSTARSNSS
LLSSDIPTNIRVHNVDDGVPEGFVLTGNPQHAVELFLEAAPEIFRREIKAAETEV
GRKFKCILTDAFLWLAAETAAAEMKASWVAYYGGGATSLTAHLYTDAIRENV
GVKEVGERMEETIGFISGMEKIRVKDTQEGVVFGNLDSVFSKTLHQMGLALP
RATAVFINSFEELDPTFTNDFRSEFKRYLNIGPLALLSSPSQTSTLVHDPHGCLA
WIEKRSTASVAYIAFGRVATPPPVELVAIAQGLESSKVPFVWSLQEMKMTHLPE
GFLDRTREQGMVVPWAPQVELLNHEAMGVFVSHGGWNSVLESVSAGVPMIC
RPIFGDHAINARSVEAVWEIGVTISSGVFTKDGFEESLDRVLVQDDGKKMKVN
AKKLEELAQEAVSTKGSSFENFGGLLDEVVNFG*
>AT3G46690
MEKRVEKRRIVLVPVAAQGHVTPMMQLGKALQSKGFLITVAQRQFNQIGSSL
QHFPGFDFVTIPESLPQSESKKLGPAEYLMNLNKTSEASFKECISQLSMQQGND
IACIIYDKLMYFCEAAAKEFKIPSVIFSTSSATIQVCYCVLSELSAEKFLIDMKD
PEKQDKVLEGLHPLRYKDLPTSGFGPLEPLLEMCREVVNKRTASAVIINTASCL
ESLSLSWLQQELGIPVYPLGPLHITASSPGPSLLQEDMSCIEWLNKQKPRSVIYI
SLGTKAHMETKEMLEMAWGLLNSNQPFLWVIRPGSVAGFEWIELLPEEVIKM
VTERGYIAKWAPQIEVLGHPAVGGFWSHCGWNSTLESIVEGVPMICRPLQGEQ
KLNAMYIESVWKIGIQLEGEVEREGVERAVKRLIIDEEGAAMRERALDLKEKL
NASVRSGGSSYNALDELVKFLNTE*
>AT3G55700
MEERKGRRIIMFPLPFPGHFNPMIELAGIFHHRGFSVTILHTSYNFPDPSRHPHF

TFRTISHNKEGEEDPLSQSETSSMDLIVLVRRLKQRYAEPFRKSVAAEVGGGET
VCCLVSDAIWGKNTEVVAEEIGVRRVVLRTGGASSFCAFAAFPLLRDKGYLPI
QDSRLDEPVTELPPLKVKDLPVMETNEPEELYRVVNDMVEGAKSSSGVIWNT
FEDLERLSLMNCSSKLQVPFFPIGPFHKYSEDPTPKTENKEDTDWLDKQDPQS
VVYASFGSLAAIEEKEFLEIAWGLRNSERPFLWVVRPGSVRGTEWLESLPLGF
MENIGDKGKIVKWANQLEVLAHPAIGAFWTHCGWNSTLESICEGVPMICTSC
FTDQHVNARYIVDVWRVGMLLERSKMEKKEIEKVLRSVMMEKGDGLRERSL
KLKERADFCLSKDGSSSKYLDKLVSHVLSFDSYAFAS*
>AT2G30150
MPWPGRGHINPMLNLCKSLVRRDPNLTVTFVVTEEWLGFIGSDPKPNRIHFAT
LPNIIPSELVRANDFIAFIDAVLTRLEEPFEQLLDRLNSPPTAIIADTYIIWAVRVG
TKRNIPVASFWTTSATILSLFINSDLLASHGHFPIEPSESKLDEIVDYIPGLSPTRL
SDLQILHGYSHQVFNIFKKSFGELYKAKYLLFPSAYELEPKAIDFFTSKFDFPVY
STGPLIPLEELSVGNENRELDYFKWLDEQPESSVLYISQGSFLSVSEAQMEEIV
VGVREAGVKFFWVARGGELKLKEALEGSLGVVVSWCDQLRVLCHAAIGGF
WTHCGYNSTLEGICSGVPLLTFPVFWDQFLNAKMIVEEWRVGMGIERKKQM
ELLIVSDEIKELVKRFMDGESEEGKEMRRRTCDLSEICRGAVAKGGSSDANIDA
FIKDITKIV*
>AT2G30140
MDPNESPPNQFRHVVAMPYPGRGHINPMMNLCKRLVRRYPNLHVTFVVTEE
WLGFIGPDPKPDRIHFSTLPNLIPSELVRAKDFIGFIDAVYTRLEEPFEKLLDSLN
SPPPSVIFADTYVIWAVRVGRKRNIPVVSLWTMSATILSFFLHSDLLISHGHALF
EPSEEEVVDYVPGLSPTKLRDLPPIFDGYSDRVFKTAKLCFDELPGARSLLFTT
AYELEHKAIDAFTSKLDIPVYAIGPLIPFEELSVQNDNKEPNYIQWLEEQPEGSV
LYISQGSFLSVSEAQMEEIVKGLRESGVRFLWVARGGELKLKEALEGSLGVVV
SWCDQLRVLCHKAVGGFWTHCGFNSTLEGIYSGVPMLAFPLFWDQILNAKMI
VEDWRVGMRIERTKKNELLIGREEIKEVVKRFMDRESEEGKEMRRRACDLSEI
SRGAVAKSGSSNVNIDEFVRHITNTN*
>AT2G36790
MAFEKNNEPFPLHFVLFPFMAQGHMIPMVDIARLLAQRGVLITIVTTPHNAAR
FKNVLNRAIESGLPINLVQVKFPYQEAGLQEGQENMDLLTTMEQITSFFKAVN
LLKEPVQNLIEEMSPRPSCLISDMCLSYTSEIAKKFKIPKILFHGMGCFCLLCVN
VLRKNREILDNLKSDKEYFIVPYFPDRVEFTRPQVPVETYVPAGWKEILEDMV
EADKTSYGVIVNSFQELEPAYAKDFKEARSGKAWTIGPVSLCNKVGVDKAER
GNKSDIDQDECLEWLDSKEPGSVLYVCLGSICNLPLSQLLELGLGLEESQRPFI
WVIRGWEKYKELVEWFSESGFEDRIQDRGLLIKGWSPQMLILSHPSVGGFLTH
CGWNSTLEGITAGLPMLTWPLFADQFCNEKLVVQILKVGVSAEVKEVMKWG
EEEKIGVLVDKEGVKKAVEELMGESDDAKERRRRAKELGESAHKAVEEGGSS
HSNITFLLQDIMQLAQSNN*
>AT1G22370
MADESSLDTKINWIPSMKNLGLKDIPSFIRATNTEDIMLNFFVHEADRAKRAS
AIILNTFDSLEHDVVRSIQSIIPQVYTIGPLHLFVNRDIDEESDIGQIGTNMWREE
MECLDWLDTKSPNSVVYVNFGSITVMSAKQLVEFAWGLAATKKDFLWVIRP
DLVAGDVPMLPPDFLIETANRRMLASWCPQEKVLSHPAVGGFLTHSGWNSTL

ESLSGGVPMVCWPFFAEQQTNCKYCCDEWEVGMEIGGDVRREEVEELVREL
MDGDKGKKMRQKAEEWQRLAEEATKPIYGSSELNFQMVVDKVLLGE*
>AT2G26480
MAEIRQRRVLMVPAPFQGHLPSMMNLASYLSSQGFSITIVRNEFNFKDISHNFP
GIKFFTIKDGLSESDVKSLGLLEFVLELNSVCEPLLKEFLTNHDDVVDFIIYDEF
VYFPRRVAEDMNLPKMVFSPSSAATSISRCVLMENQSNGLLPPQDARSQLEET
VPEFHPFRFKDLPFTAYGSMERLMILYENVSNRASSSGIIHNSSDCLENSFITTA
QEKWGVPVYPVGPLHMTNSAMSCPSLFEEERNCLEWLEKQETSSVIYISMGS
LAMTQDIEAVEMAMGFVQSNQPFLWVIRPGSINGQESLDFLPEQFNQTVTDG
RGFVVKWAPQKEVLRHRAVGGFWNHGGWNSCLESISSGVPMICRPYSGDQR
VNTRLMSHVWQTAYEIEGELERGAVEMAVRRLIVDQEGQEMRMRATILKEEV
EASVTTEGSSHNSLNNLVHAIMMQIDEQ*
>AT5G17040
MANSHVAVLAFPFGSHGQAILAVTRRLATAAPSTVFSFLNTSQSNFSLLSSDLPP
NIRVHDVSDGVPEGYVLSRNPQEAVELFLEAAPEIFRRELAVAETEVGRKVTC
MLTDAFIWFAGDMAAEMKVSWVAFWTSGTRSLLISTQISSEKQSLSKETLGCI
SGMEKIRVKDTPEGVVFGNLDSVFSKMLHQMGLALPRATTVYMNSFEELDPT
LTDNLRLKFKRYLSIGPLALLFSTSQRETPLHDPHGCLAWIKKRSTASVVYIAF
GRVMTPPPGELVVVAQGLESSKVPFVWSLQEKNMVHLPKGFLDGTREQGMV
VPWAPQVELLNHEAMGVFVSHGGWNSVLESVSAGVPMICRPIFGDHALNAR
SVEAVWEIGMTISSGVFTKDGFEESLDRVLVQDDGKKMKFNAKKLKELAQEA
VSTEGSSFENFKGLLDEVMKV*
>AT1G30530
MTKFSEPIRDSHVAVLAFFPVGAHAGPLLAVTRRLAAASPSTIFSFFNTARSNAS
LFSSDHPENIKVHDVSDGVPEGTMLGNPLEMVELFLEAAPRIFRSEIAAAEIEV
GKKVTCMLTDAFFWFAADIAAELNATWVAFWAGGANSLCAHLYTDLIRETIG
LKDVSMEETLGFIPGMENYRVKDIPEEVVFEDLDSVFPKALYQMSLALPRASA
VFISSFEELEPTLNYNLRSKLKRFLNIAPLTLLSSTSEKEMRDPHGCFAWMGKR
SAASVAYISFGTVMEPPPEELVAIAQGLESSKVPFVWSLKEKNMVHLPKGFLD
RTREQGIVVPWAPQVELLKHEAMGVNVTHCGWNSVLESVSAGVPMIGRPILA
DNRLNGRAVEVVWKVGVMMDNGVFTKEGFEKCLNDVFVHDDGKTMKANA
KKLKEKLQEDFSMKGSSLENFKILLDEIVKV*
>AT5G05880
MEKSNGLRVILFPLPLQGCINPMIQLAKILHSRGFSITVIHTCFNAPKASSHPLFT
FIQIQDGLSETETRTRDVKLLITLLNQNCESPVRECLRKLLQSAKEEKQRISCLI
NDSGWIFTQHLAKSLNLMRLAFNTYKISFFRSHFVLPQLRREMFLPLQDSEQD
DPVEKFPPLRKKDLLRILEADSVQGDSYSDMILEKTKASSGLIFMSCEELDQDS
LSQSREDFKVPIFAIGPSHSHFPASSSSLFTPDETCIPWLDRQEDKSVIYVSIGSL
VTINETELMEIAWGLSNSDQPFLWVVRVGSVNGTEWIEAIPEYFIKRLNEKGKI
VKWAPQQEVLKHRAIGGFLTHNGWNSTVESVCEGVPMICLPFRWDQLLNAR
FVSDVWMVGIHLEGRIERDEIERAIRRLLLETEGEAIRERIQLLKEKVGRSVKQ
NGSAYQSLQNLINYISSF*
>AT2G28080
MADVRNPTKNHHGHHHLHALLIPYPFQGHVNPFVHLAIKLASQGITVTFVNT

HYIHHQITNGSDGDIFAGVRSESGLDIRYATVSDGLPVGFDRSLNHDTYQSSLL
HVFYAHVEELVASLVGGDGGVNVMIADTFFVWPSVVARKFGLVCVSFWTEA
ALVFSLYYHMDLLRIHGHFGAQETRSDLIDYIPGVAAINPKDTASYLQETDTSS
VVHQIIFKAFEDVKKVDFVLCNTIQQFEDKTIKALNTKIPFYAIGPIIPFNNQTG
SVTTSLWSESDCTQWLNTKPKSSVLYISFGSYAHVTKKDLVEIAHGILLSKVNF
VWVVRPDIVSSDETNPLPEGFETEAGDRGIVIPWCCQMTVLSHESVGGFLTHC
GWNSILETIWCEVPVLCFPLLTDQVTNRKLVVDDWEIGINLCEDKSDFGRDEV
GRNINRLMCGVSKEKIGRVKMSLEGAVRNSGSSSEMNLGLFIDGLLSKVGLSN
GKA*
>AT3G21560
MELESSPPLPPHVMLVSFPGQGHVNPLLRLGKLLASKGLLITFVTTESWGKKM
RISNKIQDRVLKPVGKGYLRYDFFDDGLPEDDEASRTNLTILRPHLELVGKREI
KNLVKRYKEVTKQPVTCLINNPFVSWVCDVAEDLQIPCAVLWVQSCACLAAY
YYYHHNLVDFPTKTEPEIDVQISGMPLLKHDEIPSFIHPSSPHSALREVIIDQIKR
LHKTFSIFIDTFNSLEKDIIDHMSTLSLPGVIRPLGPLYKMAKTVAYDVVKVNIS
EPTDPCMEWLDSQPVSSVVYISFGTVAYLKQEQIDEIAYGVLNADVTFLWVIR
QQELGFNKEKHVLPEEVKGKGKIVEWCSQEKVLSHPSVACFVTHCGWNSTM
EAVSSGVPTVCFPQWGDQVTDAVYMIDVWKTGVRLSRGEAEERLVPREEVAE
RLREVTKGEKAIELKKNALKWKEEAEAAVARGGSSDRNLEKFVEKLGAKPV
GKVQNGSHNHVLAGSIKSF*
>AT5G05860
MEEKRNGLRVILFPLPLQGCINPMLQLANILHVRGFSITVIHTRFNAPKASSHPL
FTFLQIPDGLSETEIQDGVMSLLAQINLNAESPFRDCLRKVLLESKESERVTCLI
DDCGWLFTQSVSESLKLPRLVLCTFKATFFNAYPSLPLIRTKGYLPVSESEAED
SVPEFPPLQKRDLSKVFGEFGEKLDPFLHAVVETTIRSSGLIYMSCEELEKDSLT
LSNEIFKVPVFAIGPFHSYFSASSSSLFTQDETCILWLDDQEDKSVIYVSLGSVV
NITETEFLEIACGLSNSKQPFLWVVRPGSVLGAKWIEPLSEGLVSSLEEKGKIV
KWAPQQEVLAHRATGGFLTHNGWNSTLESICEGVPMICLPGGWDQMLNSRF
VSDIWKIGIHLEGRIEKKEIEKAVRVLMEESEGNKIRERMKVLKDEVEKSVKQ
GGSSFQSIETLANHILLL*
>AT1G22340
MESHVVHNAQKPHVVCVPYPAQGHINPMLKVAKLLYAKGFHVTFVNTLYNH
NRLLRSRGPNALDGFPSFRFESIPDGLPETDGDRTQHTPTVCMSIEKNCLAPFK
EILRRINDKDDVPPVSCIVSDGVMSFTLDAAEELGVPEVIFWTNSACGFMTILH
FYLFIEKGLSPFKDESYMSKEHLDTVIDWIPSMKNLRLKDIPSYIRTTNPDNIM
LNFLIREVERSKRASAIILNTFDELEHDVIQSMQSILPPVYSIGPLHLLVKEEINE
ASEIGQMGLNLWREEMECLDWLDTKTPNSVLFVNFGCITVMSAKQLEEFAW
GLAASRKEFLWVIRPNLVVGEAMVVLPQEFLAETIDRRMLASWCPQEKVLSH
PAIGGFLTHCGWNSTLESLAGGVPMICWPCFSEQPTNCKFCCDEWGVGIEIGK
DVKREEVETVVRELMDGEKGKKLREKAEEWRRLAEEATRYKHGSSVMNLET
LIHKVFLENLR*
>AT4G14090
MATSVNGSHRRPHYLLVTFPAQGHINPALQLANRLIHHGATVTYSTAVSAHRR
MGEPPSTKGLSFAWFTDGFDDGLKSFEDQKIYMSELKRCGSNALRDIIKANLD

ATTETEPITGVIYSVLVPWVSTVAREFHLPTTLLWIEPATVLDIYYYYFNTSYKH
LFDVEPIKLPKLPLITTGDLPSFLQPSKALPSALVTLREHIEALETESNPKILVNT
FSALEHDALTSVEKLKMIPIGPLVSSSEGKTDLFKSSDEDYTKWLDSKLERSVI
YISLGTHADDLPEKHMEALTHGVLATNRPFLWIVREKNPEEKKKNRFLELIRG
SDRGLVVGWCSQTAVLAHCAVGCFVTHCGWNSTLESLESGVPVVAFPQFADQ
CTTAKLVEDTWRIGVKVKVGEEGDVDGEEIRRCLEKVMSGGEEAEEMRENA
EKWKAMAVDAAAEGGPSDLNLKGFVDEDE*
>AT1G24100
MAETTPKVKGHVVILPYPVQGHLNPMVQFAKRLVSKNVKVTIATTTYTASSIT
TPSLSVEPISDGFDFIPIGIPGFSVDTYSESFKLNGSETLTLLIEKFKSTDSPIDCLI
YDSFLPWGLEVARSMELSAASFFTNNLTVCSVLRKFSNGDFPLPADPNSAPFRI
RGLPSLSYDELPSFVGRHWLTHPEHGRVLLNQFPNHENADWLFVNGFEGLEE
TQDCENGESDAMKATLIGPMIPSAYLDDRMEDDKDYGASLLKPISKECMEWL
ETKQAQSVAFVSFGSFGILFEKQLAEVAIALQESDLNFLWVIKEAHIAKLPEGF
VESTKDRALLVSWCNQLEVLAHESIGCFLTHCGWNSTLEGLSLGVPMVGVPQ
WSDQMNDAKFVEEVWKVGYRAKEEAGEVIVKSEELVRCLKGVMEGESSVKI
RESSKKWKDLAVKAMSEGGSSDRSINEFIESLGK*
>AT4G15500
MEMESSLPHVMLVSFPGQGHISPLLRLGKIIASKGLIVTFVTTEEPLGKKMRQA
NNIQDGVLKPVGLGFLRFEFFEDGFVYKEDFDLLQKSLEVSGKREIKNLVKKY
EKQPVRCLINNAFVPWVCDIAEELQIPSAVLWVQSCACLAAYYYYHHQLVKF
PTETEPEITVDVPFKPLTLKHDEIPSFLHPSSPLSSIGGTILEQIKRLHKPFSVLIET
FQELEKDTIDHMSQLCPQVNFNPIGPLFTMAKTIRSDIKGDISKPDSDCIEWLD
SREPSSVVYISFGTLAFLKQNQIDEIAHGILNSGLSCLWVLRPPLEGLAIEPHVL
PLELEEKGKIVEWCQQEKVLAHPAVACFLSHCGWNSTMEALTSGVPVICFPQ
WGDQVTNAVYMIDVFKTGLRLSRGASDERIVPREEVAERLLEATVGEKAVEL
RENARRWKEEAESAVAYGGTSERNFQEFVDKLVDVKTMTNINNVV*
>AT3G46720
MEKNAEKKRIVLVPFPLQGHITPMMQLGQALNLKGFSITVALGDSNRVSSTQH
FPGFQFVTIPETIPLSQHEALGVVEFVVTLNKTSETSFKDCIAHLLLQHGNDIAC
IIYDELMYFSEATAKDLRIPSVIFTTGSATNHVCSCILSKLNAEKFLIDMKDPEV
QNMVVENLHPLKYKDLPTSGMGPLERFLEICAEVVNKRTASAVIINTSSCLESS
SLSWLKQELSIPVYPLGPLHITTSANFSLLEEDRSCIEWLNKQKLRSVIYISVGSI
AHMETKEVLEMAWGLYNSNQPFLWVIRPGTESMPVEVSKIVSERGCIVKWAP
QNEVLVHPAVGGFWSHCGWNSTLESIVEGVPMICRPFNGEQKLNAMYIESVW
RVGVLLQGEVERGCVERAVKRLIVDDEGVGMRERALVLKEKLNASVRSGGSS
YNALDELVHYLEAEYRNT*
>AT4G15490
MDPSRHTHVMLVSFPGQGHVNPLLRLGKLIASKGLLVTFVTTEKPWGKKMR
QANKIQDGVLKPVGLGFIRFEFFSDGFADDDEKRFDFDAFRPHLEAVGKQEIK
NLVKRYNKEPVTCLINNAFVPWVCDVAEELHIPSAVLWVQSCACLTAYYYYH
HRLVKFPTKTEPDISVEIPCLPLLKHDEIPSFLHPSSPYTAFGDIILDQLKRFENH
KSFYLFIDTFRELEKDIMDHMSQLCPQAIISPVGPLFKMAQTLSSDVKGDISEP
ASDCMEWLDSREPSSVVYISFGTIANLKQEQMEEIAHGVLSSGLSVLWVVRPP

MEGTFVEPHVLPRELEEKGKIVEWCPQERVLAHPAIACFLSHCGWNSTMEALT
AGVPVVCFPQWGDQVTDAVYLADVFKTGVRLGRGAAEEMIVSREVVAEKLL
EATVGEKAVELRENARRWKAEAEAAVADGGSSDMNFKEFVDKLVTKHVTRE
DNGEH*
>AT3G02100
MDNNSNKRMGRPHVVVIPYPAQGHVLPLISFSRYLAKQGIQITFINTEFNHNRI
ISSLPNSPHEDYVGDQINLVSIPDGLEDSPEERNIPGKLSESVLRFMPKKVEELIE
RMMAETSGGTIISCVVADQSLGWAIEVAAKFGIRRTAFCPAAAASMVLGFSIQ
KLIDDGLIDSDGTVRVNKTIQLSPGMPKMETDKFVWVCLKNKESQKNIFQLM
LQNNNSIESTDWLLCNSVHELETAAFGLGPNIVPIGPIGWAHSLEEGSTSLGSFL
PHDRDCLDWLDRQIPGSVIYVAFGSFGVMGNPQLEELAIGLELTKRPVLWVTG
DQQPIKLGSDRVKVVRWAPQREVLSSGAIGCFVSHCGWNSTLEGAQNGIPFLC
IPYFADQFINKAYICDVWKIGLGLERDARGVVPRLEVKKKIDEIMRDGGEYEE
RAMKVKEIVMKSVAKDGISCENLNKFVNWIKSQVN*
>AT1G06000
MTTTTTKKPHVLVIPFPQSGHMVPHLDLTHQILLRGATVTVLVTPKNSSYLDA
LRSLHSPEHFKTLILPFPSHPCIPSGVESLQQLPLEAIVHMFDALSRLHDPLVDF
LSRQPPSDLPDAILGSSFLSPWINKVADAFSIKSISFLPINAHSISVMWAQEDRSF
FNDLETATTESYGLVINSFYDLEPEFVETVKTRFLNHHRIWTVGPLLPFKAGVD
RGGQSSIPPAKVSAWLDSCPEDNSVVYVGFGSQIRLTAEQTAALAAALEKSSV
RFIWAVRDAAKKVNSSDNSVEEDVIPAGFEERVKEKGLVIRGWAPQTMILEHR
AVGSYLTHLGWGSVLEGMVGGVMLLAWPMQADHFFNTTLIVDKLRAAVRV
GENRDSVPDSDKLARILAESAREDLPERVTLMKLREKAMEAIKEGGSSYKNL
DELVAEMCL*
>AT5G37950
MMQLGRAHSLKGFSITVAQTKFNYLNPSKDLADFQFITIPESLPASDLKTLGPI
WFIIKLNKECEISFKKCLGQFLLQQQEEIACVIYDEFMYFAEAAAKEFNLPKVI
FSTENATAFACRSAMCKLYAKDGIAPLTEGCGREEELVPELHPLRYKDLPTSAF
APVEASVEVFKSSCEKGTASSMIINTVSCLEISSLEWLQQELKIPIYPIGPLYMVS
SAPPTSLLDENESCIDWLNKQKPSSVIYISLGSFTLLETKEVLEMASGLVSSNQ
YFLWAIRPGSILGSELSNEELFSMMEIPDRGYIVKWATQKQVLAHAAVGAFWS
HCGWNSTLESIGEGIPIVGLLLLIKR*
>AT2G23260
MGSSEGQETHVLMVTLPFQGHINPMLKLAKHLSLSSKNLHINLATIESARDLL
STVEKPRYPVDLVFFSDGLPKEDPKAPETLLKSLNKVGAMNLSKIIEEKRYSCII
SSPFTPWVPAVAASHNISCAILWIQACGAYSVYYRYYMKTNSFPDLEDLNQTV
ELPALPLLEVRDLPSFMLPSGGAHFYNLMAEFADCLRYVKWVLVNSFYELESE
IIESMADLKPVIPIGPLVSPFLLGDGEEETLDGKNLDFCKSDDCCMEWLDKQA
RSSVVYISFGSMLETLENQVETIAKALKNRGLPFLWVIRPKEKAQNVAVLQEM
VKEGQGVVLEWSPQEKILSHEAISCFVTHCGWNSTMETVVAGVPVVAYPSWT
DQPIDARLLVDVFGIGVRMRNDSVDGELKVEEVERCIEAVTEGPAAVDIRRRA
AELKRVARLALAPGGSSTRNLDLFISDITIA*
>AT5G05890
MEKSNGLRVILFPLPLQGCINPMIQLAKILHSRGFSITVIHTCFNAPKASSHPLFT

FLEIPDGLSETEKRTNNTKLLLTLLNRNCESPFRECLSKLLQSADSETGEEKQRI
SCLIADSGWMFTQPIAQSLKLPILVLSVFTVSFFRCQFVLPKLRREVYLPLQDS
EQEDLVQEFPPLRKKDIVRILDVETDILDPFLDKVLQMTKASSGLIFMSCEELD
HDSVSQAREDFKIPIFGIGPSHSHFPATSSSLSTPDETCIPWLDKQEDKSVIYVSY
GSIVTISESDLIEIAWGLRNSDQPFLLVVRVGSVRGREWIETIPEEIMEKLNEKG
KIVKWAPQQDVLKHRAIGGFLTHNGWSSTVESVCEAVPMICLPFRWDQMLNA
RFVSDVWMVGINLEDRVERNEIEGAIRRLLVEPEGEAIRERIEHLKEKVGRSFQ
QNGSAYQSLQNLIDYISSF*
>AT1G10400
MELEKVHVVLFPYLSKGHMIPMLQLARLLLSHSFAGDISVTVFTTPLNRPFIVD
SLSGTKATIVDVPFPDNVPEIPPGVECTDKLPALSSSLFVPFTRATKSMQADFER
ELMSLPRVSFMVSDGFLWWTQESARKLGFPRLVFFGMNCASTVICDSVFQNQ
LLSNVKSETEPVSVPEFPWIKVRKCDFVKDMFDPKTTTDPGFKLILDQVTSMN
QSQGIIFNTFDDLEPVFIDFYKRKRKLKLWAVGPLCYVNNFLDDEVEEKVKPS
WMKWLDEKRDKGCNVLYVAFGSQAEISREQLEEIALGLEESKVNFLWVVKG
NEIGKGFEERVGERGMMVRDEWVDQRKILEHESVRGFLSHCGWNSLTESICS
EVPILAFPLAAEQPLNAILVVEELRVAERVVAASEGVVRREEIAEKVKELMEGE
KGKELRRNVEAYGKMAKKALEEGIGSSRKNLDNLINEFCNNGT*
>AT2G23250
MVALAFQGHLNPMLKFAKHLARTNLHFTLATTEQARDLLSSTADEPHRPVDL
AFFSDGLPKDDPRDPDTLAKSLKKDGAKNLSKIIEEKRFDCIISVPFTPWVPAV
AAAHNIPCAILWIQACGAFSVYYRYYMKTNPFPDLEDLNQTVELPALPLLEVR
DLPSLMLPSQGANVNTLMAEFADCLKDVKWVLVNSFYELESEIIESMSDLKPII
PIGPLVSPFLLGNDEEKTLDMWKVDDYCMEWLDKQARSSVVYISFGSILKSLE
NQVETIATALKNRGVPFLWVIRPKEKGENVQVLQEMVKEGKGVVTEWGQQE
KILSHMAISCFITHCGWNSTIETVVTGVPVVAYPTWIDQPLDARLLVDVFGIGV
RMKNDAIDGELKVAEVERCIEAVTEGPAAADMRRRATELKHAARSAMSPGGS
SAQNLDSFISDIPIT*
>AT4G15550
MANNNSNSPTGPHFLFVTFPAQGHINPSLELAKRLAGTISGARVTFAASISAYN
RRMFSTENVPETLIFATYSDGHDDGFKSSAYSDKSRQDATGNFMSEMRRRGK
ETLTELIEDNRKQNRPFTCVVYTILLTWVAELAREFHLPSALLWVQPVTVFSIF
YHYFNGYEDAISEMANTPSSSIKLPSLPLLTVRDIPSFIVSSNVYAFLLPAFREQI
DSLKEEINPKILINTFQELEPEAMSSVPDNFKIVPVGPLLTLRTDFSSRGEYIEWL
DTKADSSVLYVSFGTLAVLSKKQLVELCKALIQSRRPFLWVITDKSYRNKEDE
QEKEEDCISSFREELDEIGMVVSWCDQFRVLNHRSIGCFVTHCGWNSTLESLV
SGVPVVAFPQWNDQMMNAKLLEDCWKTGVRVMEKKEEEGVVVVDSEEIRR
CIEEVMEDKAEEFRGNATRWKDLAAEAVREGGSSFNHLKAFVDEHM*
>AT5G05900
MDKSNGLRVILFPLPLQGCINPMIQLAKILHSRGFSITVIHTRFNAPKASNHPLF
TFLQIPDGLSETETRTHDITLLLTLLNRSCESPFRECLTKLLQSADSETGEEKQRI
SCLIDDSGWIFTQPVAQSFNLPRLVLNTYKVSFFRDHFVLPQLRREMYLPLQDS
EQGDDPVEEFPPLRKKDLLQILDQESEQLDSYSNMILETTKASSGLIFVSTCEE
LDQDSLSQAREDYQVPIFTIGPSHSYFPGSSSSLFTVDETCIPWLDKQEDKSVIY

VSFGSISTIGEAEFMEIAWALRNSDQPFLWVVRGGSVVHGAEWIEQLHEKGKI
VNWAPQQEVLKHQAIGGFLTHNGWNSTVESVFEGVPMICMPFVWDQLLNAR
FVSDVWMVGLHLEGRIERNVIEGMIRRLFSETEGKAIRERMEILKENVGRSVK
PKGSAYRSLQHLIDYITYF*
