# Supplementary material for: Association mapping for total polyphenol content, total flavonoid content and antioxidant activity in barley
Source: BMC Genomics. 2018 Jan 25;19:81. doi: 10.1186/s12864-018-4483-6 (PMC5784657; doi:10.1186/s12864-018-4483-6)
Supplement: Supplementary file 14 — Correlation analysis of TPC, FLC and AOA in 223 accessions. ** represent significant correlation at P < 0.01. (DOCX 15 kb) [file 12864_2018_4483_MOESM14_ESM.docx]

**Table S7.** Correlation analysis of TPC, FLC and AOA in 223 accessions.

|  | TPC | FLC | AOA |
| --- | --- | --- | --- |
| TPC | 1 |  |  |
| FLC | 0.44** | 1 |  |
| AOA | 0.729** | 0.424** | 1 |

Note: ** represent significant correlation at P<0.01.
